# Supplementary figures and images for: The acetylase activity of Cdu1 regulates bacterial exit from infected cells by protecting Chlamydia effectors from degradation (part 1 of 2)
Source: eLife. 2024 Feb 15;12:RP87386. doi: 10.7554/eLife.87386 (PMC10942603; doi:10.7554/eLife.87386)

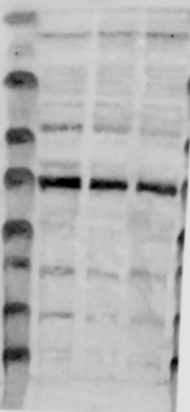

Supplement: Figure 1—figure supplement 2—source data 1. [file elife-87386-fig1-figsupp2-data1.zip › 02_Figure 1-figure supplement 2-source data 1/alpha Tubulin wb-original.png]

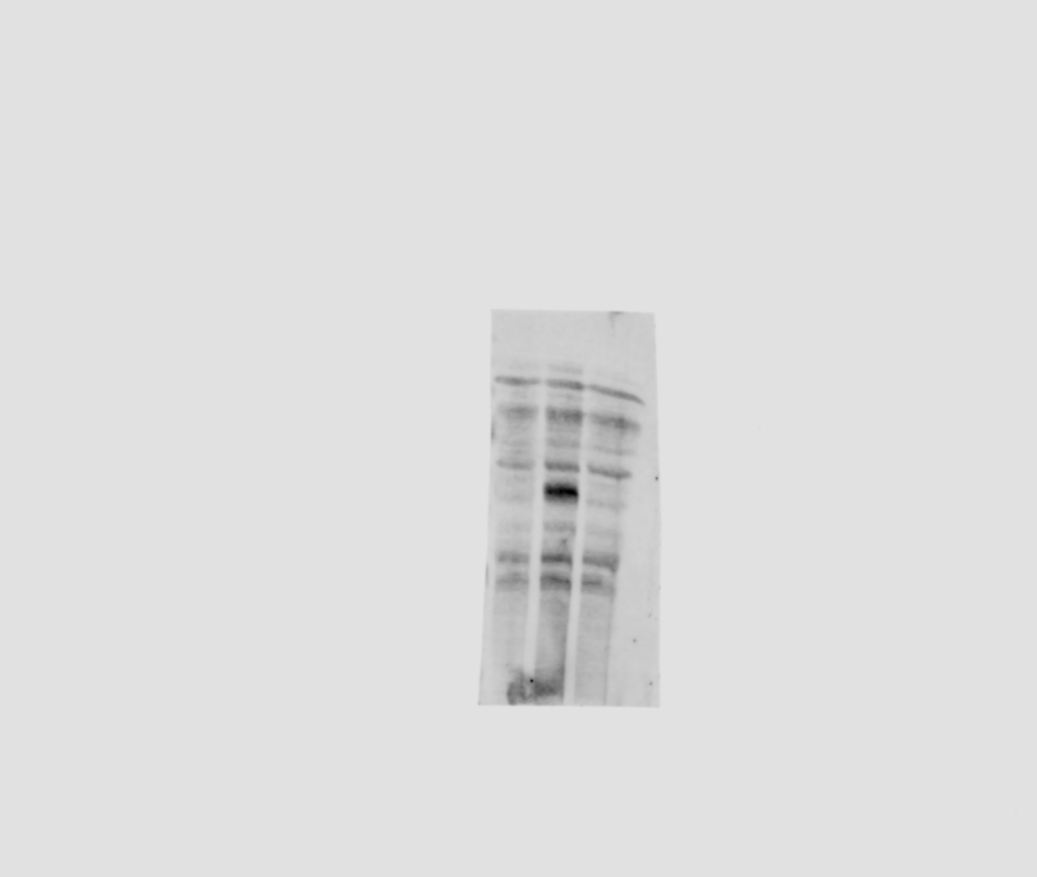

Supplement: Figure 1—figure supplement 2—source data 1. [file elife-87386-fig1-figsupp2-data1.zip › 02_Figure 1-figure supplement 2-source data 1/Cdu1 WB signal-original.png]

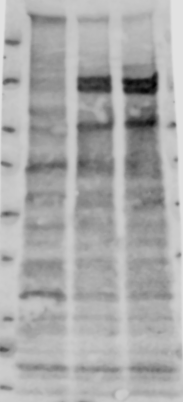

Supplement: Figure 1—figure supplement 2—source data 1. [file elife-87386-fig1-figsupp2-data1.zip › 02_Figure 1-figure supplement 2-source data 1/RpoB WB signal-original.png]

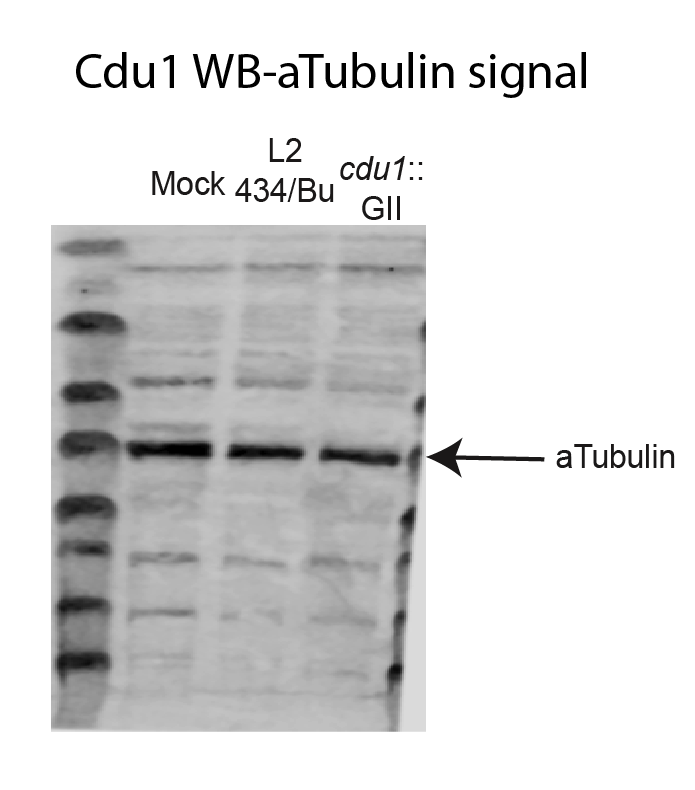

Supplement: Figure 1—figure supplement 2—source data 2. [file elife-87386-fig1-figsupp2-data2.zip › 03_Figure 1-figure supplement 2-source data 2/alpha Tubulin wb-original annotated.png]

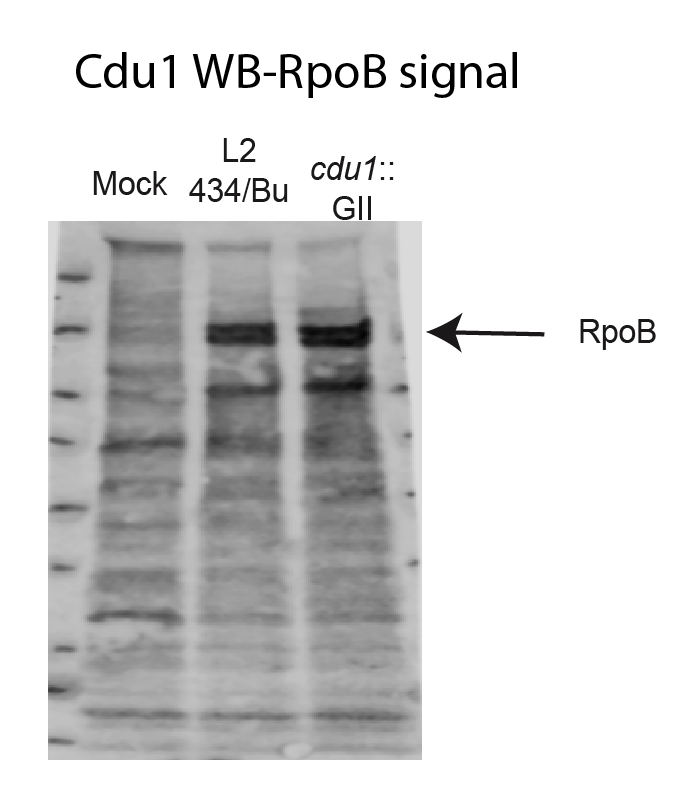

Supplement: Figure 1—figure supplement 2—source data 2. [file elife-87386-fig1-figsupp2-data2.zip › 03_Figure 1-figure supplement 2-source data 2/RpoB WB signal-original annotated.png]

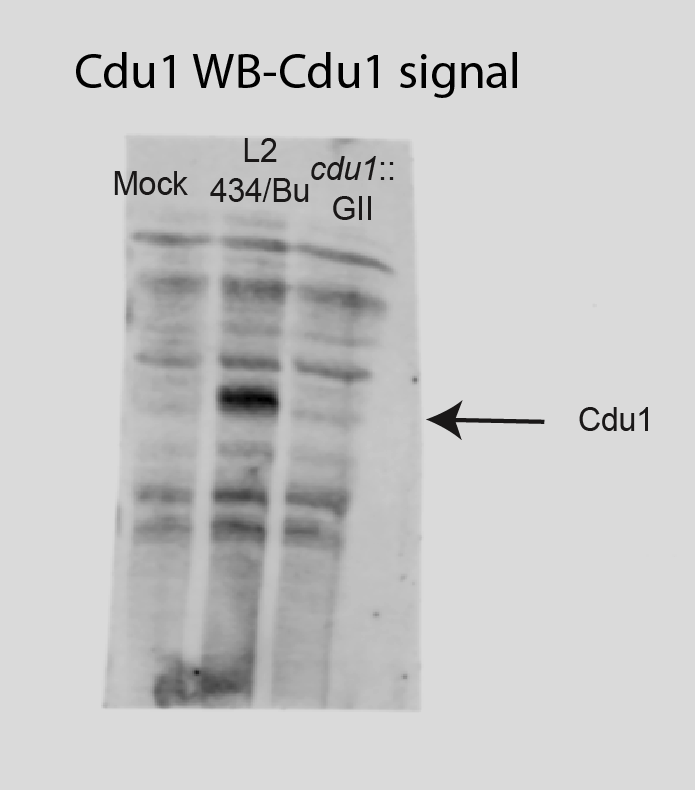

Supplement: Figure 1—figure supplement 2—source data 2. [file elife-87386-fig1-figsupp2-data2.zip › 03_Figure 1-figure supplement 2-source data 2/Cdu1 WB signal-original annotated.png]

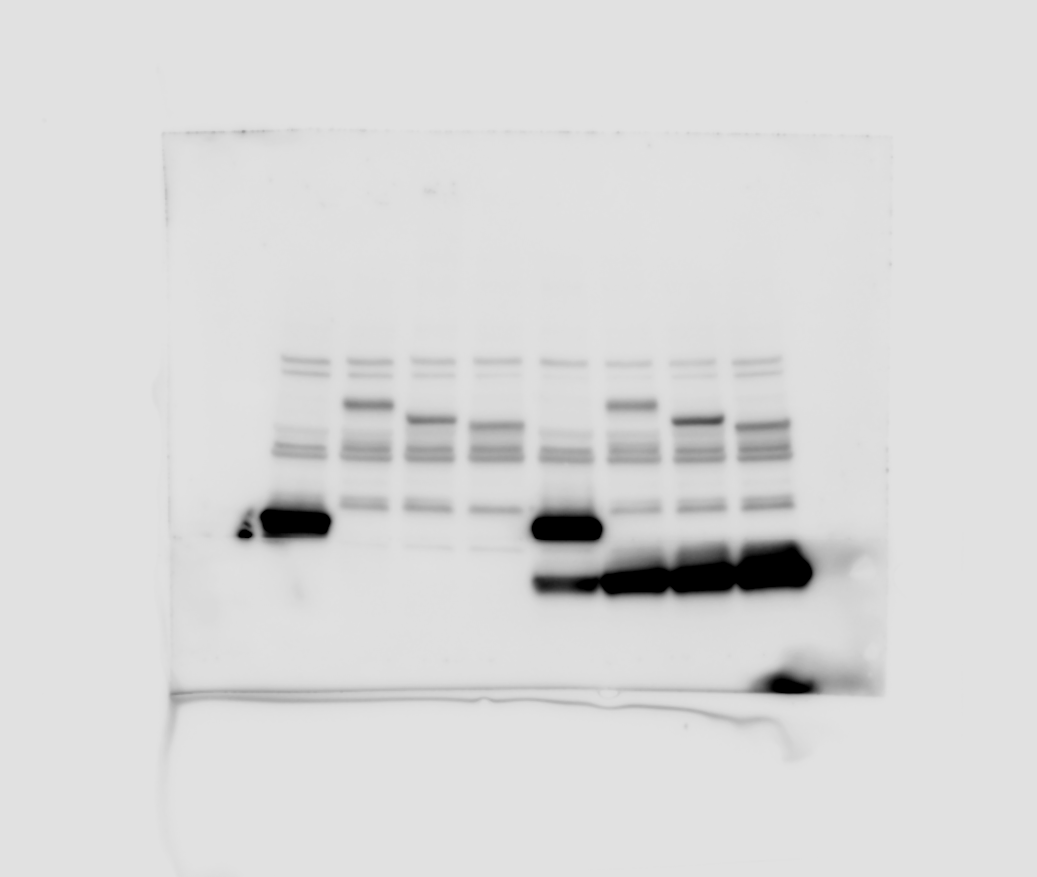

Supplement: Figure 2—source data 2. [file elife-87386-fig2-data2.zip › 08_Figure 2-source data 2/01_Cdu1_InaC interaction Figure_Raw data_Input GFP (Cdu1 variants).tif]

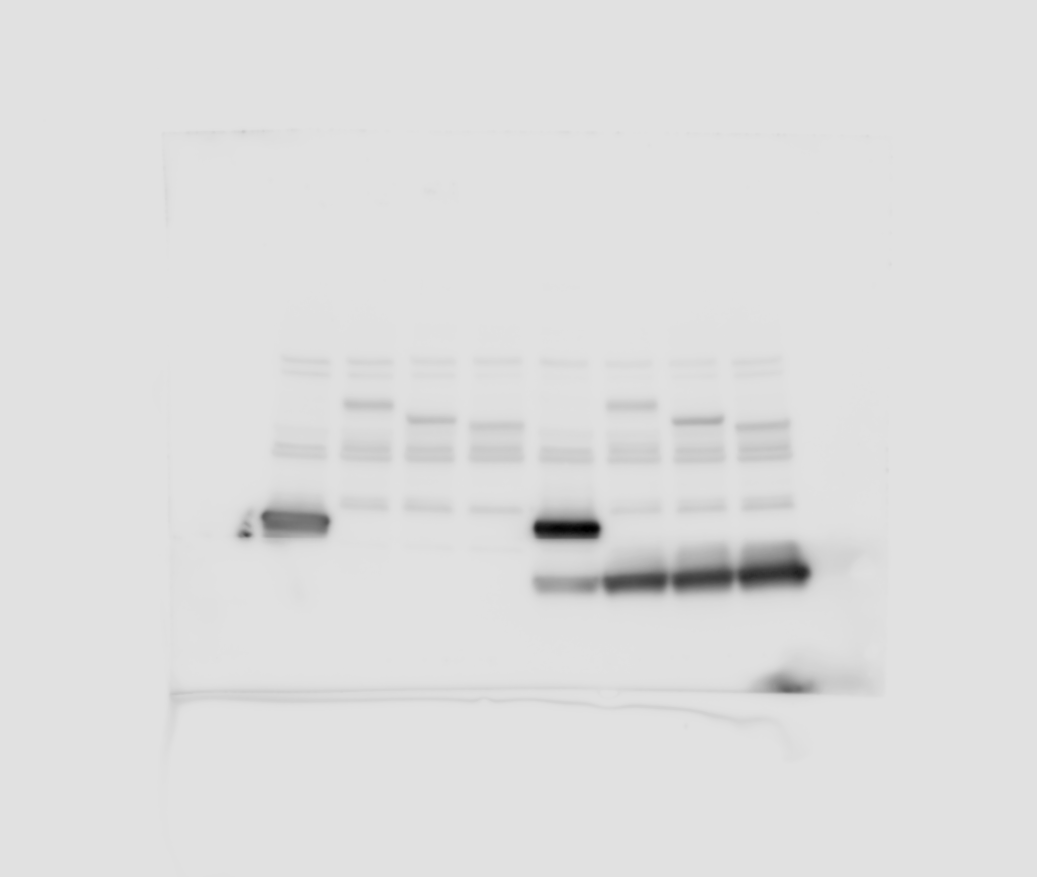

Supplement: Figure 2—source data 2. [file elife-87386-fig2-data2.zip › 08_Figure 2-source data 2/02_Cdu1_InaC interaction Figure_Raw data_Input Flag (InaC).tif]

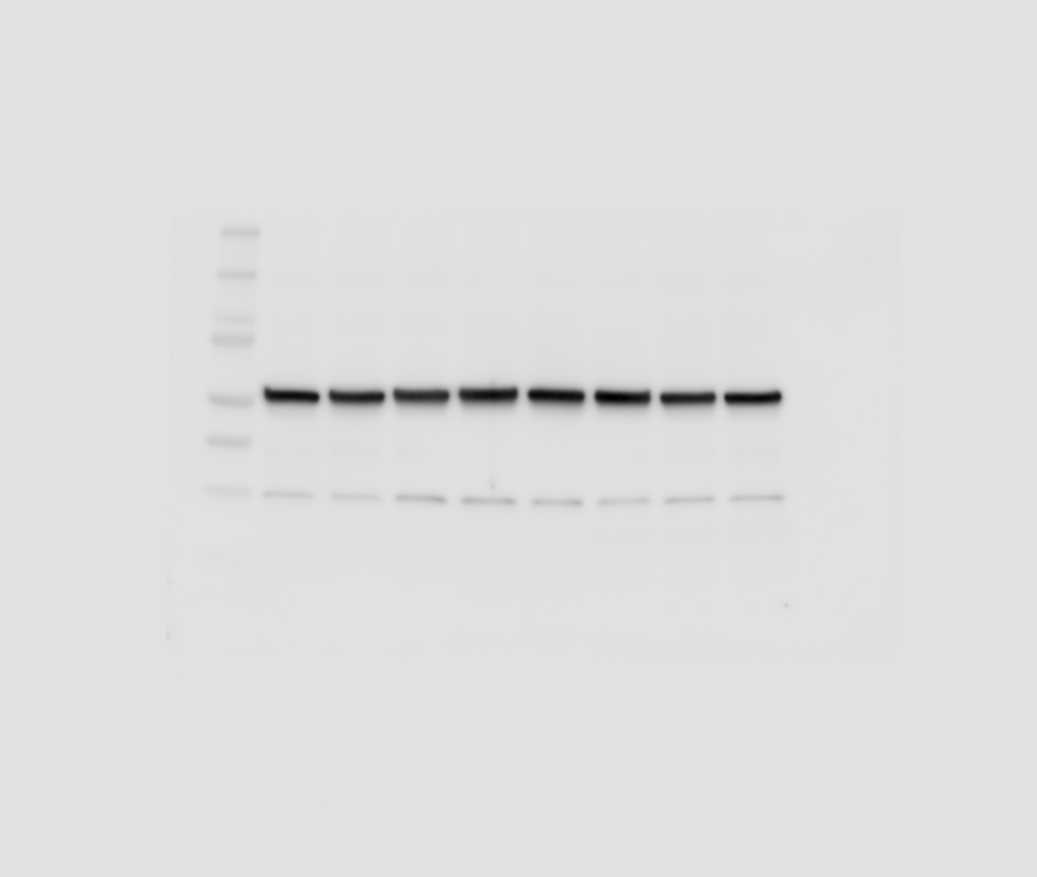

Supplement: Figure 2—source data 2. [file elife-87386-fig2-data2.zip › 08_Figure 2-source data 2/03_Cdu1_InaC interaction Figure_Raw data_Input Tubulin.tif]

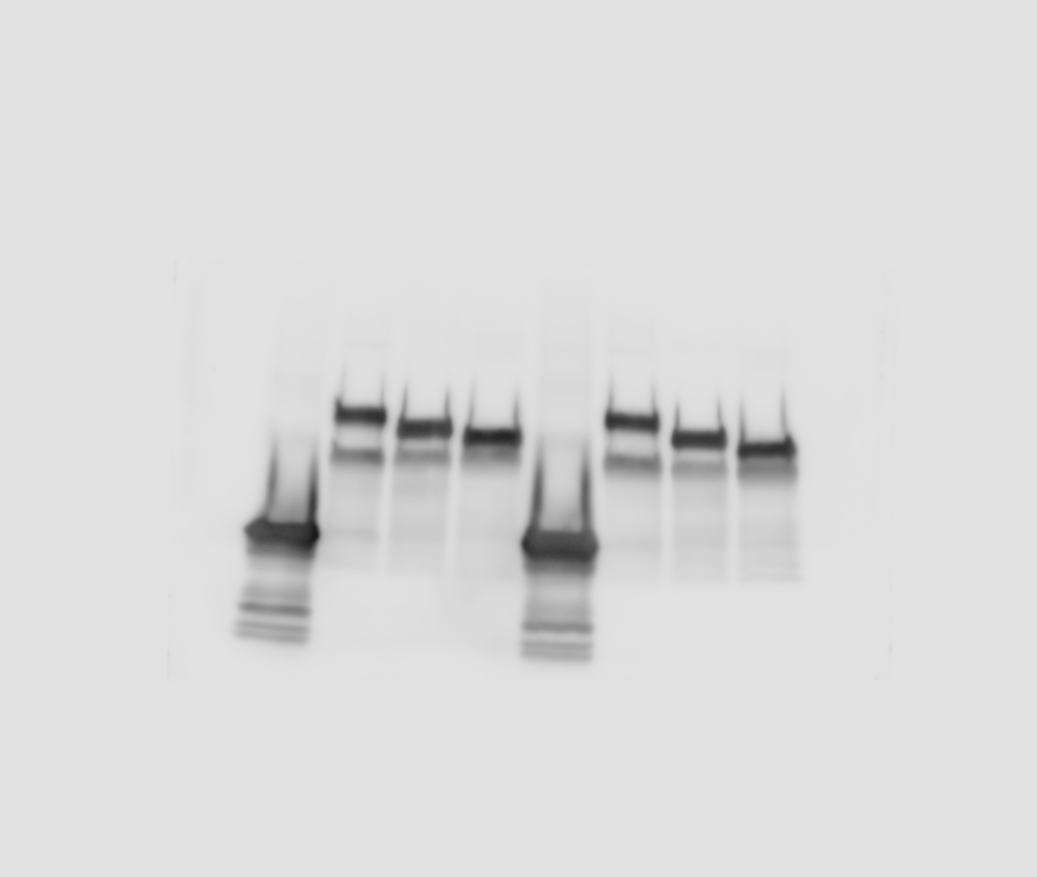

Supplement: Figure 2—source data 2. [file elife-87386-fig2-data2.zip › 08_Figure 2-source data 2/04_Cdu1_InaC interaction Figure_Raw data_IP GFP (cdu1 variants).tif]

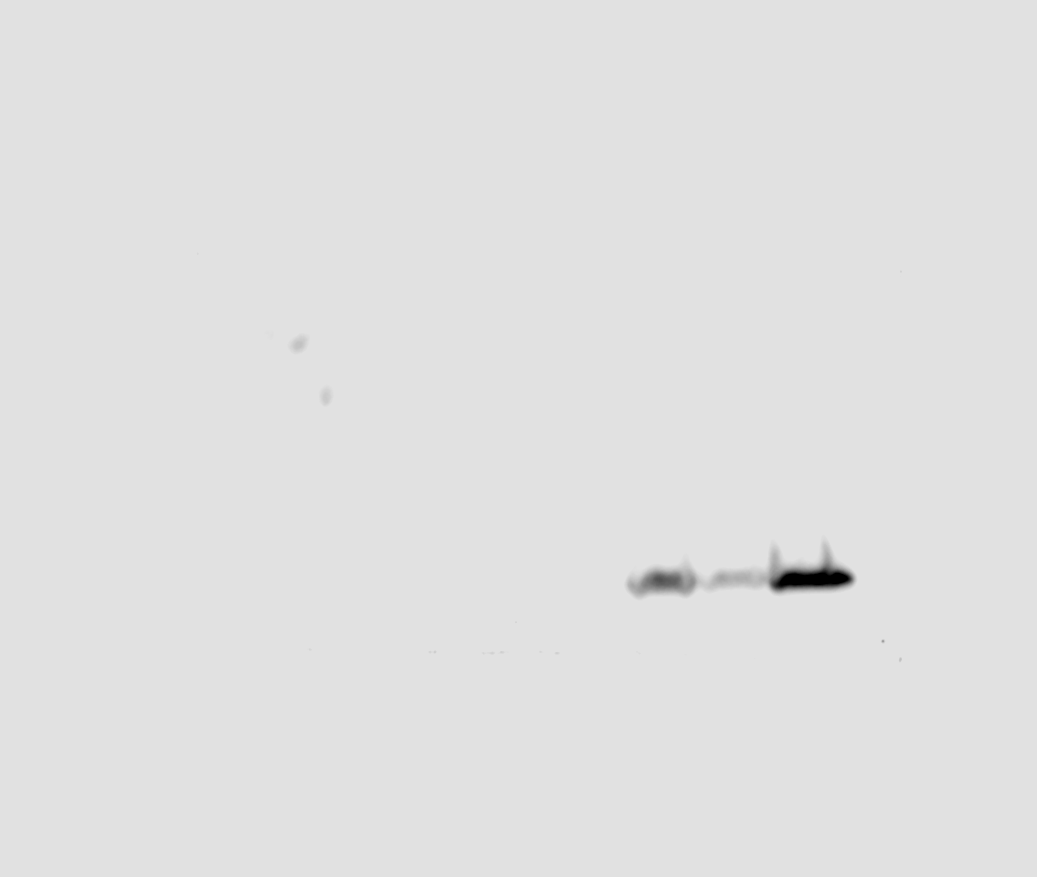

Supplement: Figure 2—source data 2. [file elife-87386-fig2-data2.zip › 08_Figure 2-source data 2/05_Cdu1_InaC interaction Figure_Raw data_IP Flag (InaC).tif]

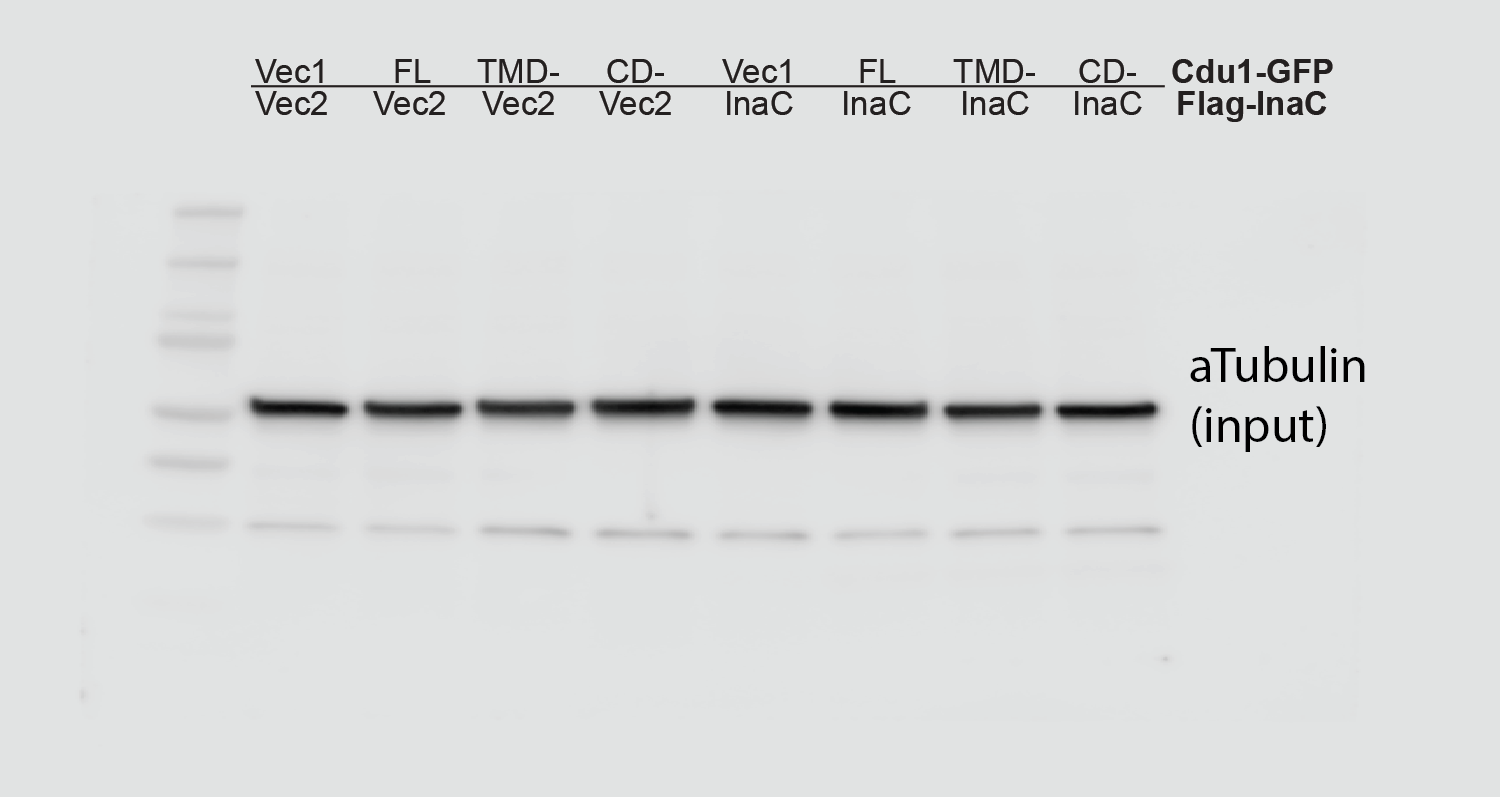

Supplement: Figure 2—source data 3. [file elife-87386-fig2-data3.zip › 09_Figure 2-source data 3/03_Cdu1_InaC interaction Figure_Raw data_Input Tubulin_annotated.png]

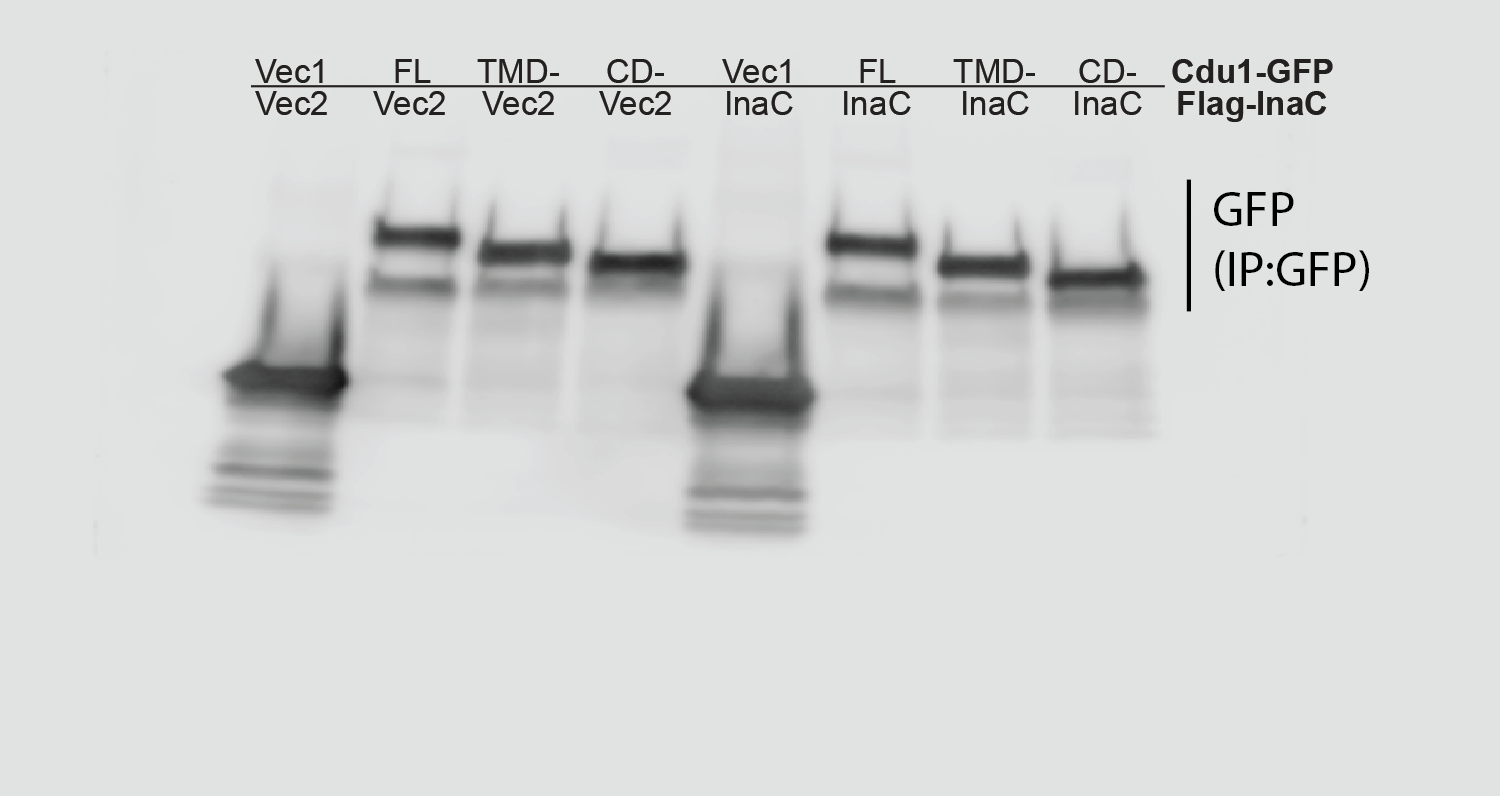

Supplement: Figure 2—source data 3. [file elife-87386-fig2-data3.zip › 09_Figure 2-source data 3/04_Cdu1_InaC interaction Figure_Raw data_IP GFP (cdu1 variants)_annotated.png]

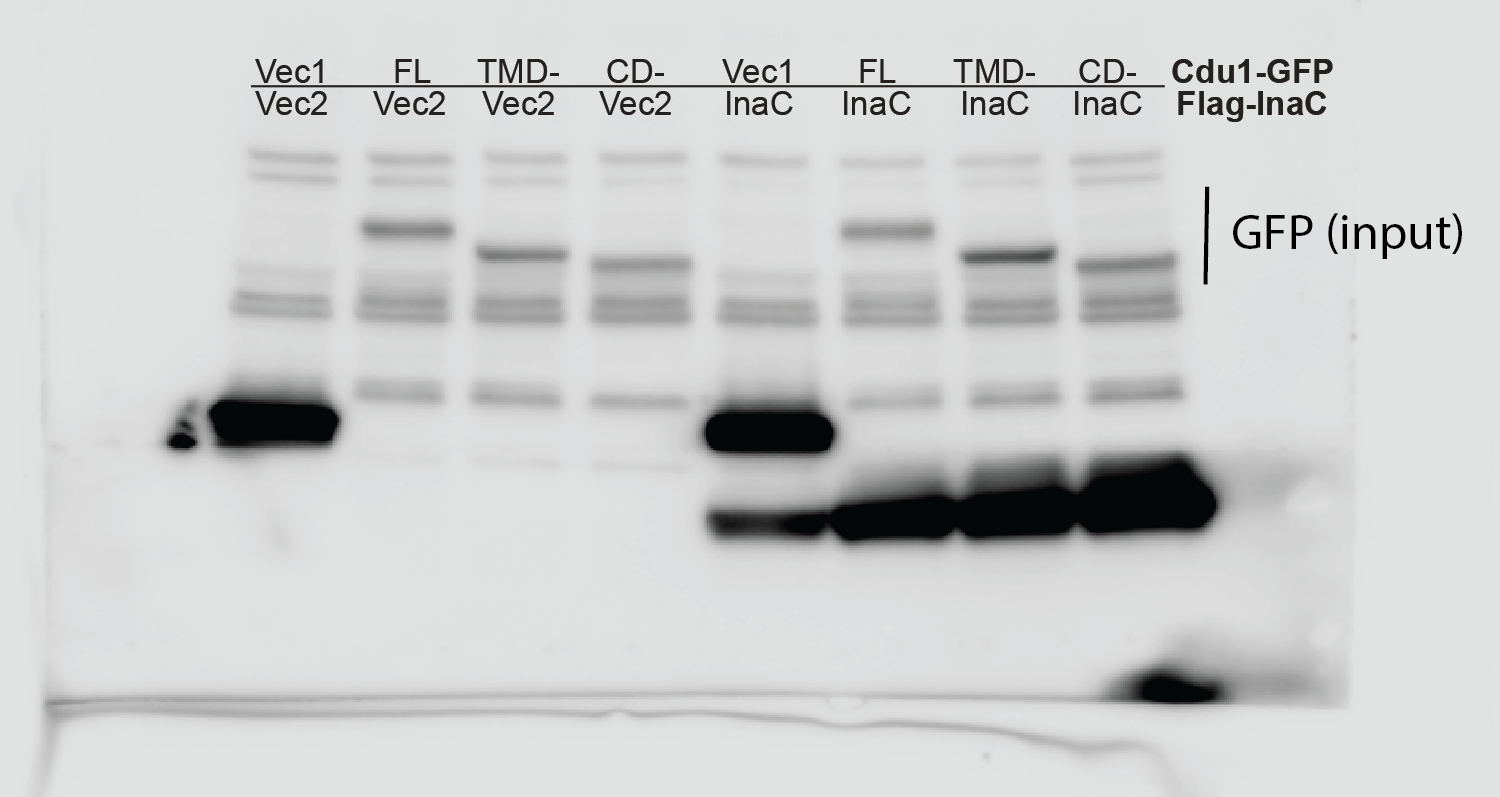

Supplement: Figure 2—source data 3. [file elife-87386-fig2-data3.zip › 09_Figure 2-source data 3/01_Cdu1_InaC interaction Figure_Raw data_Input GFP (Cdu1 variants)_annotated.png]

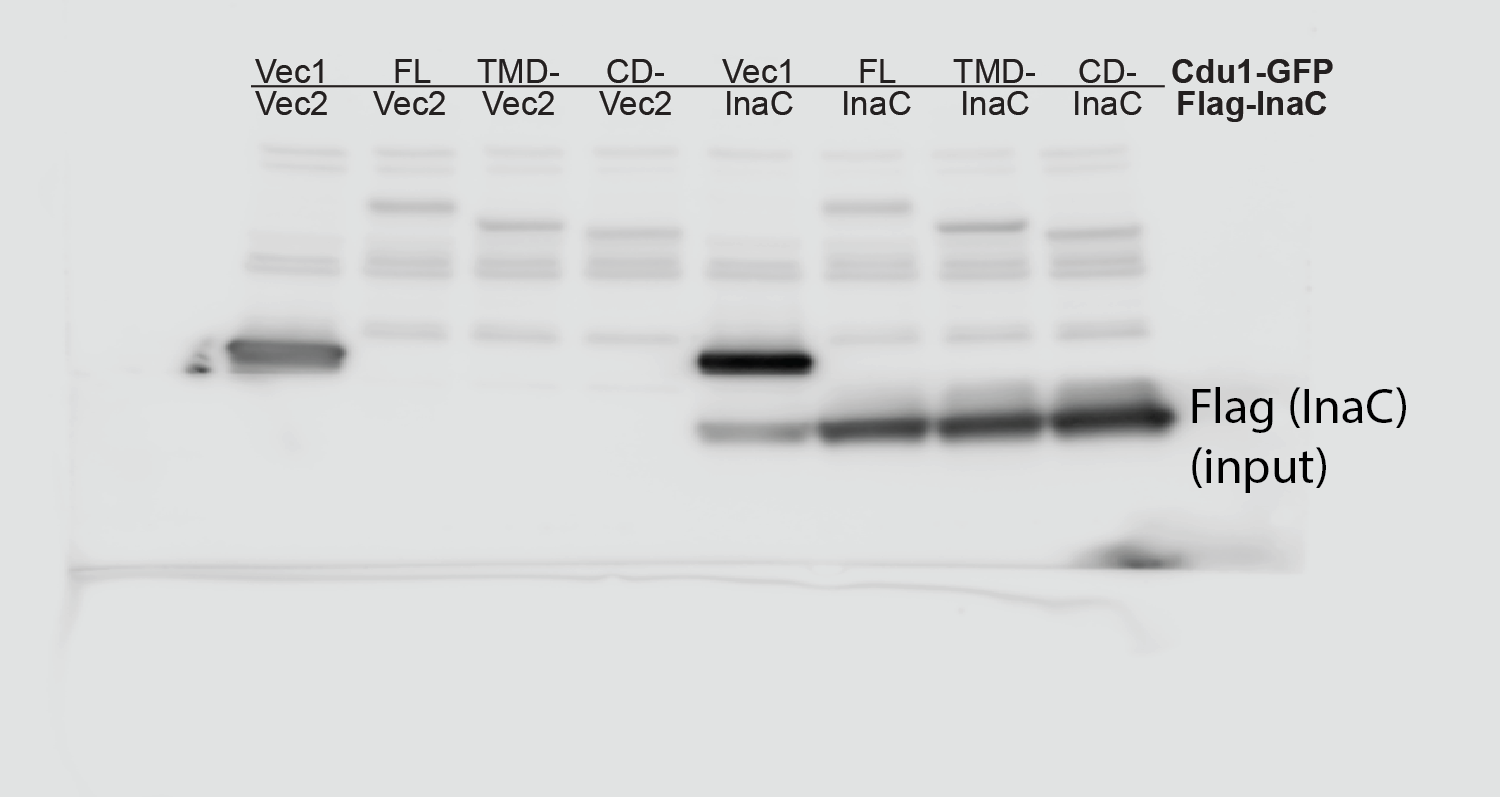

Supplement: Figure 2—source data 3. [file elife-87386-fig2-data3.zip › 09_Figure 2-source data 3/02_Cdu1_InaC interaction Figure_Raw data_Input Flag (InaC)_annotated.png]

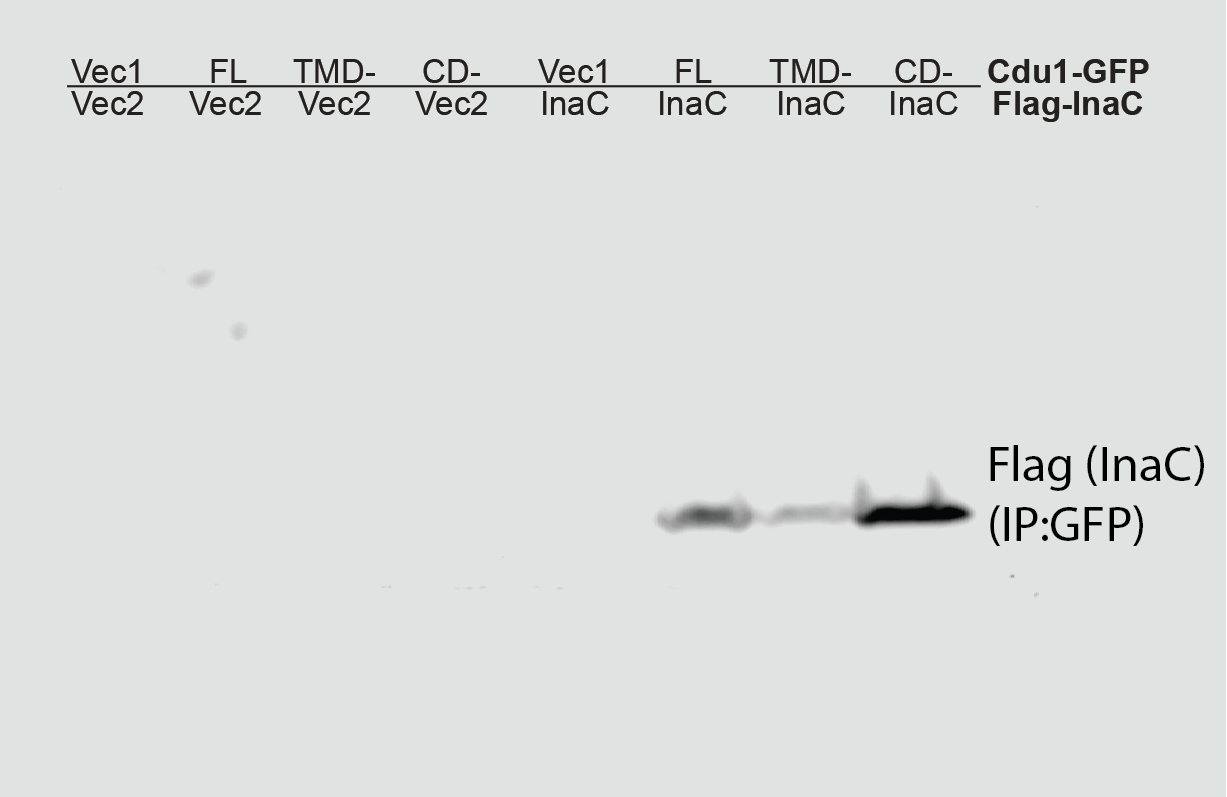

Supplement: Figure 2—source data 3. [file elife-87386-fig2-data3.zip › 09_Figure 2-source data 3/05_Cdu1_InaC interaction Figure_Raw data_IP Flag (InaC)_annotated.png]

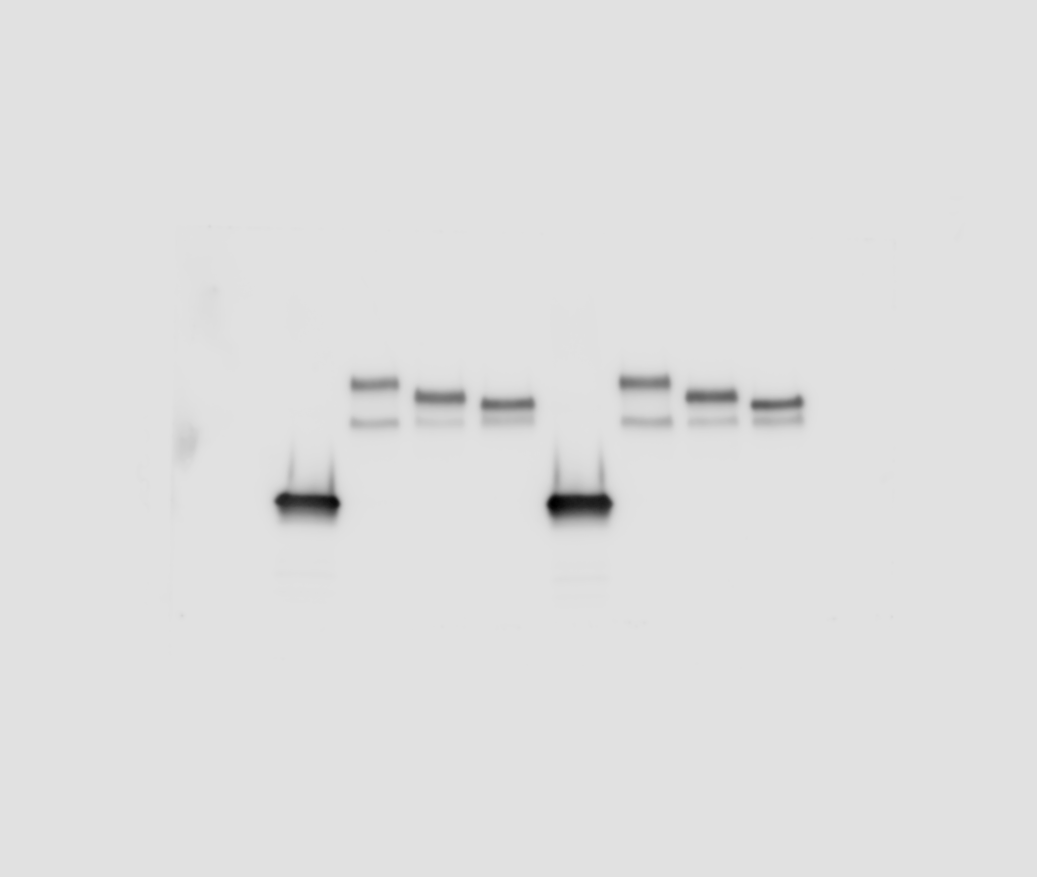

Supplement: Figure 2—source data 4. [file elife-87386-fig2-data4.zip › 10_Figure 2-source data 4/04_Cdu1_IpaM interaction Figure_Raw data_IP GFP_ GFP.tif]

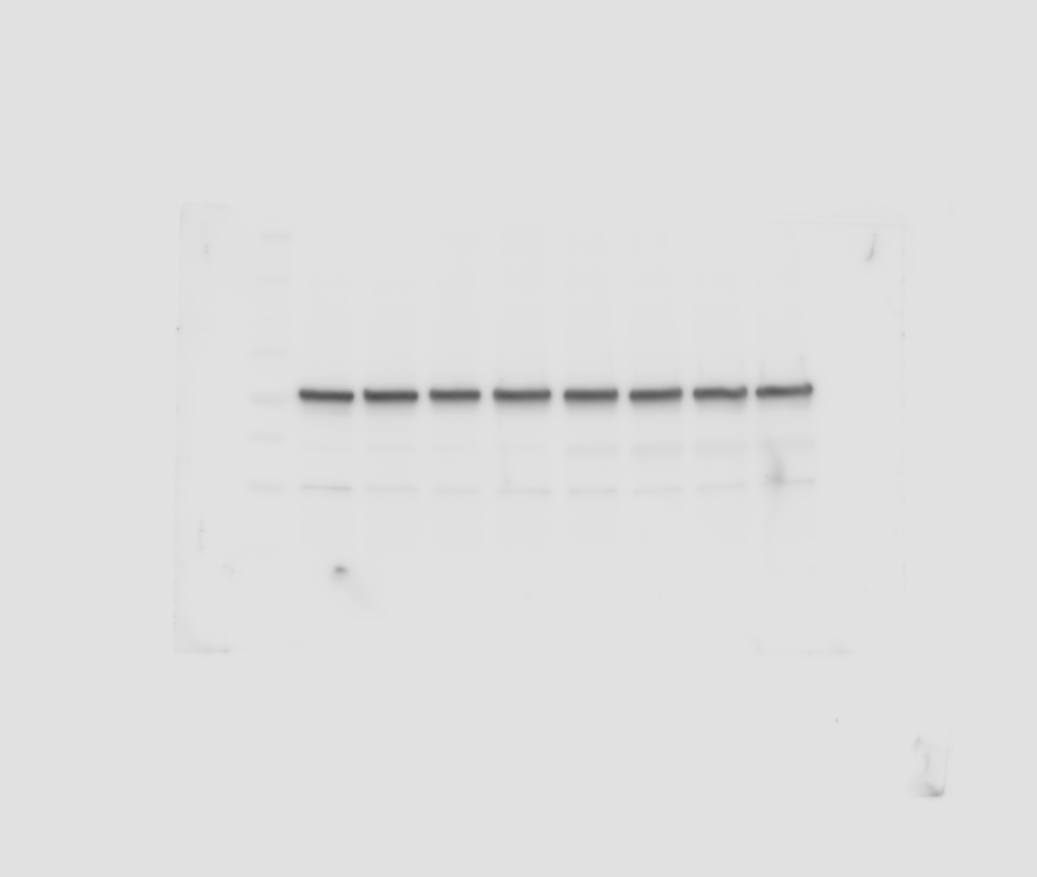

Supplement: Figure 2—source data 4. [file elife-87386-fig2-data4.zip › 10_Figure 2-source data 4/03_Cdu1_IpaM interaction Figure_Raw data_input Tubulin.tif]

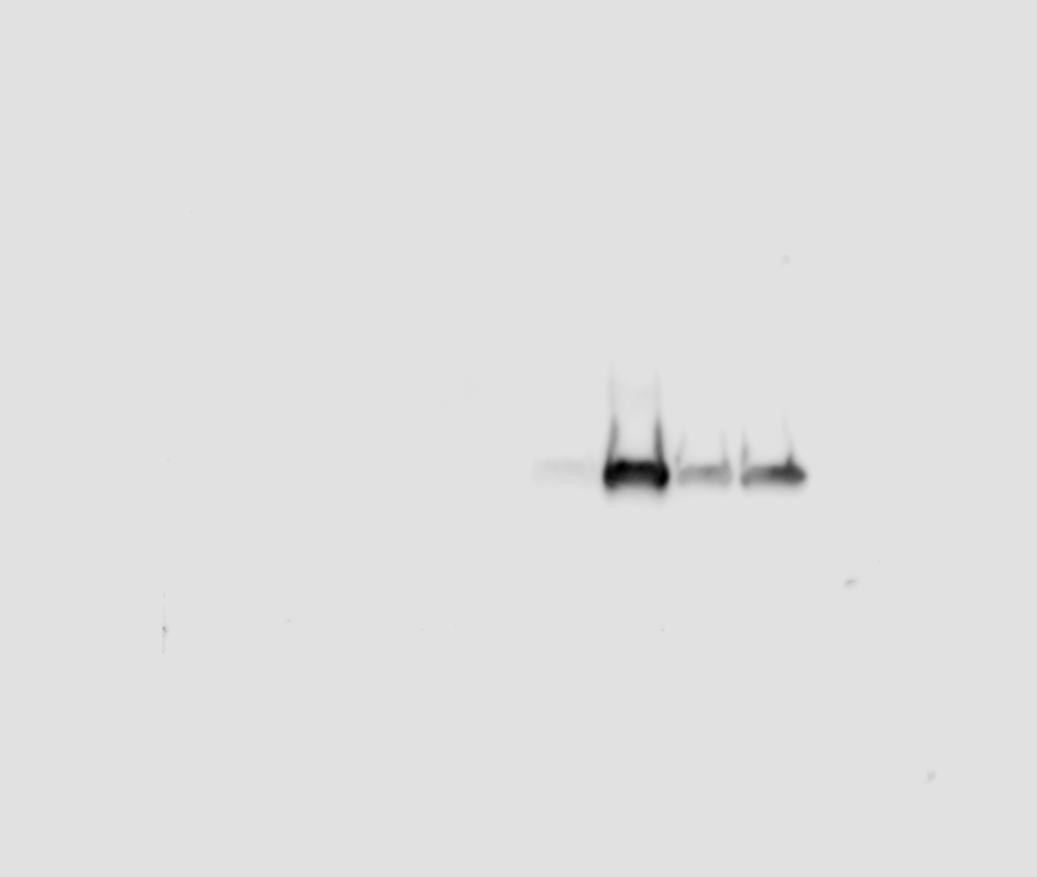

Supplement: Figure 2—source data 4. [file elife-87386-fig2-data4.zip › 10_Figure 2-source data 4/05_Cdu1_IpaM interaction Figure_Raw data_IP GFP_ V5 (IpaM).tif]

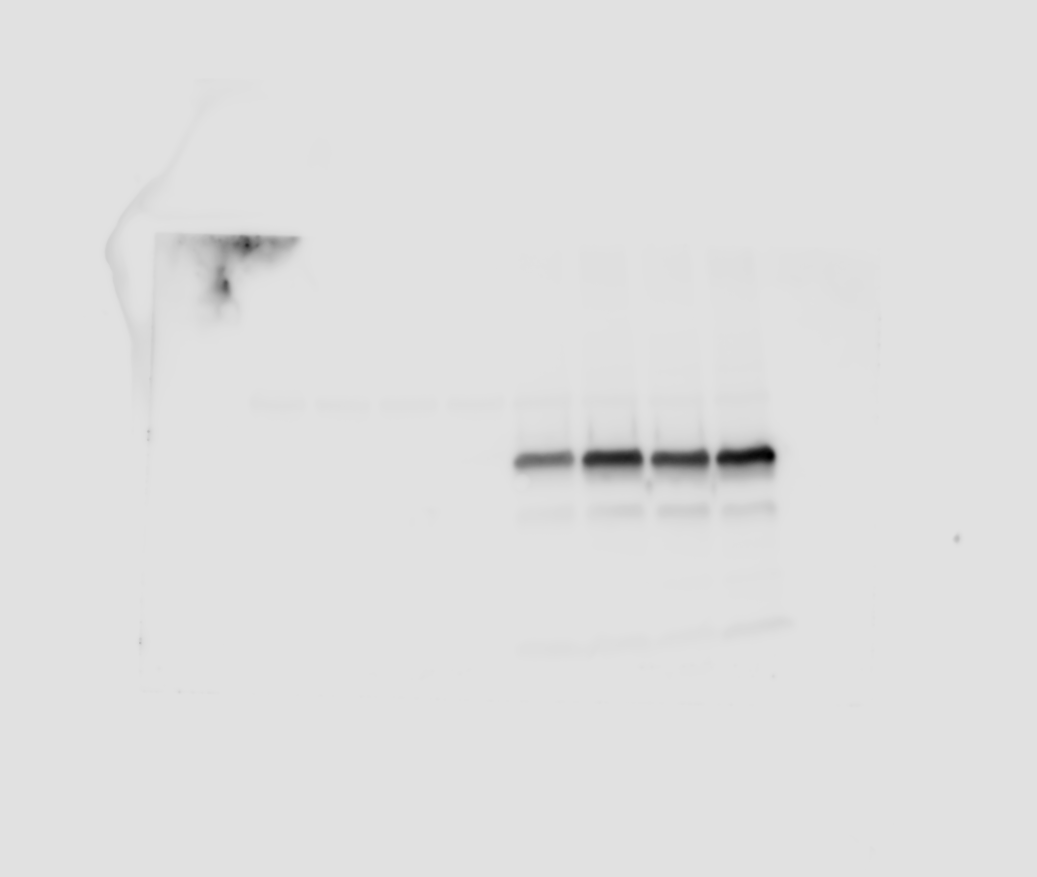

Supplement: Figure 2—source data 4. [file elife-87386-fig2-data4.zip › 10_Figure 2-source data 4/02_Cdu1_IpaM interaction Figure_Raw data_input V5 (IpaM).tif]

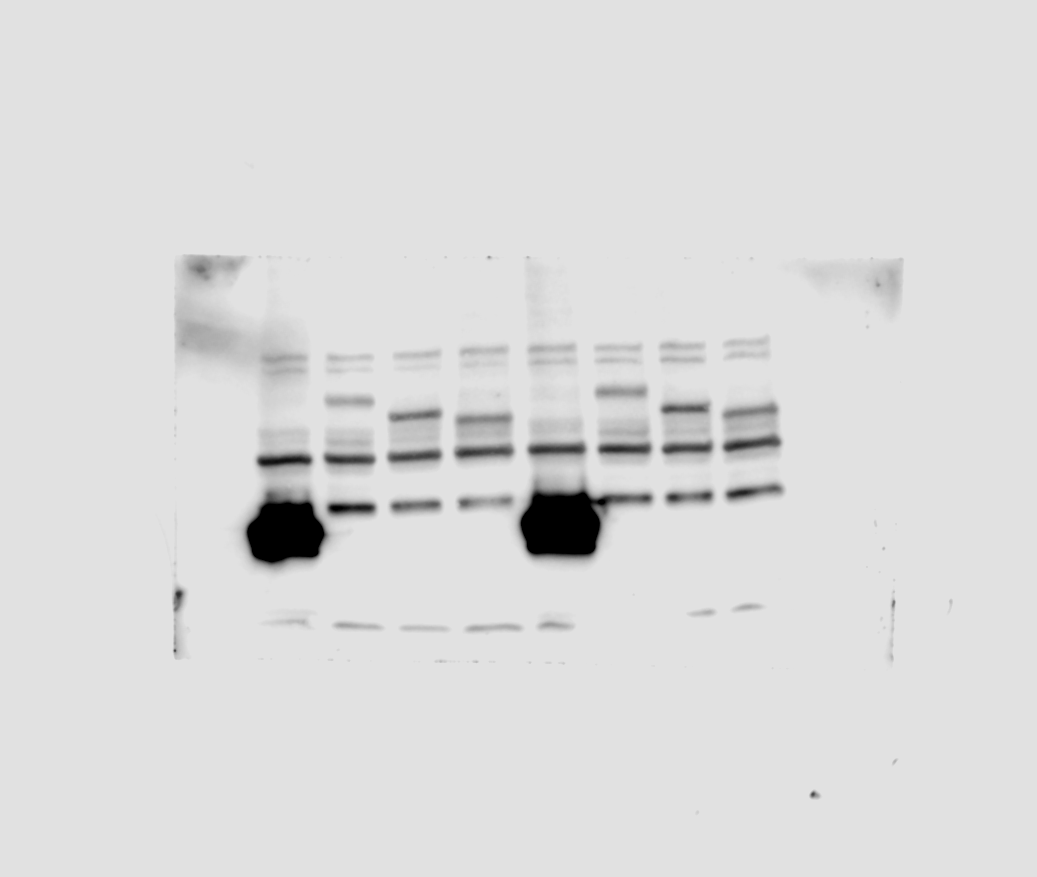

Supplement: Figure 2—source data 4. [file elife-87386-fig2-data4.zip › 10_Figure 2-source data 4/01_Cdu1_IpaM interaction Figure_Raw data_input GFP.tif]

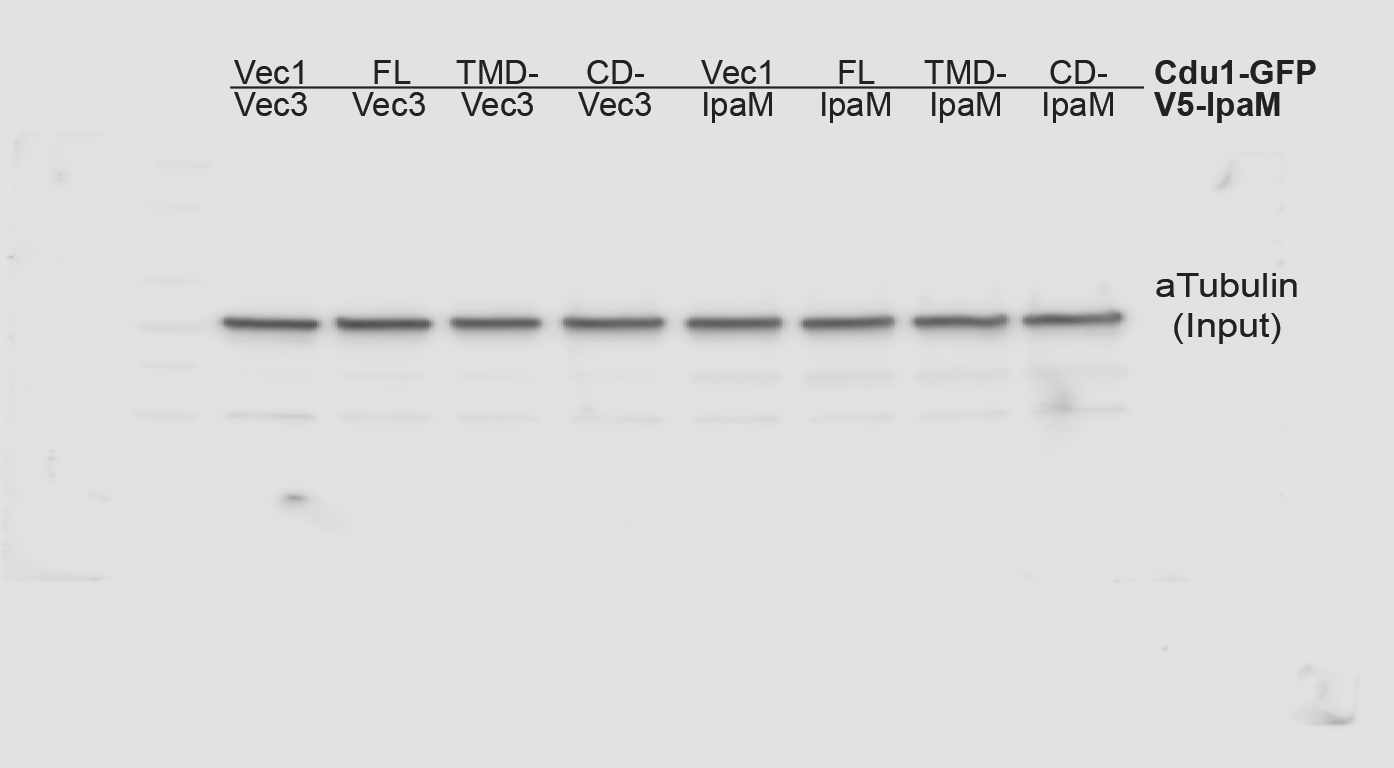

Supplement: Figure 2—source data 5. [file elife-87386-fig2-data5.zip › 11_Figure 2-source data 5/03_Cdu1_IpaM interaction Figure_Raw data_input Tubulin_Annotated.png]

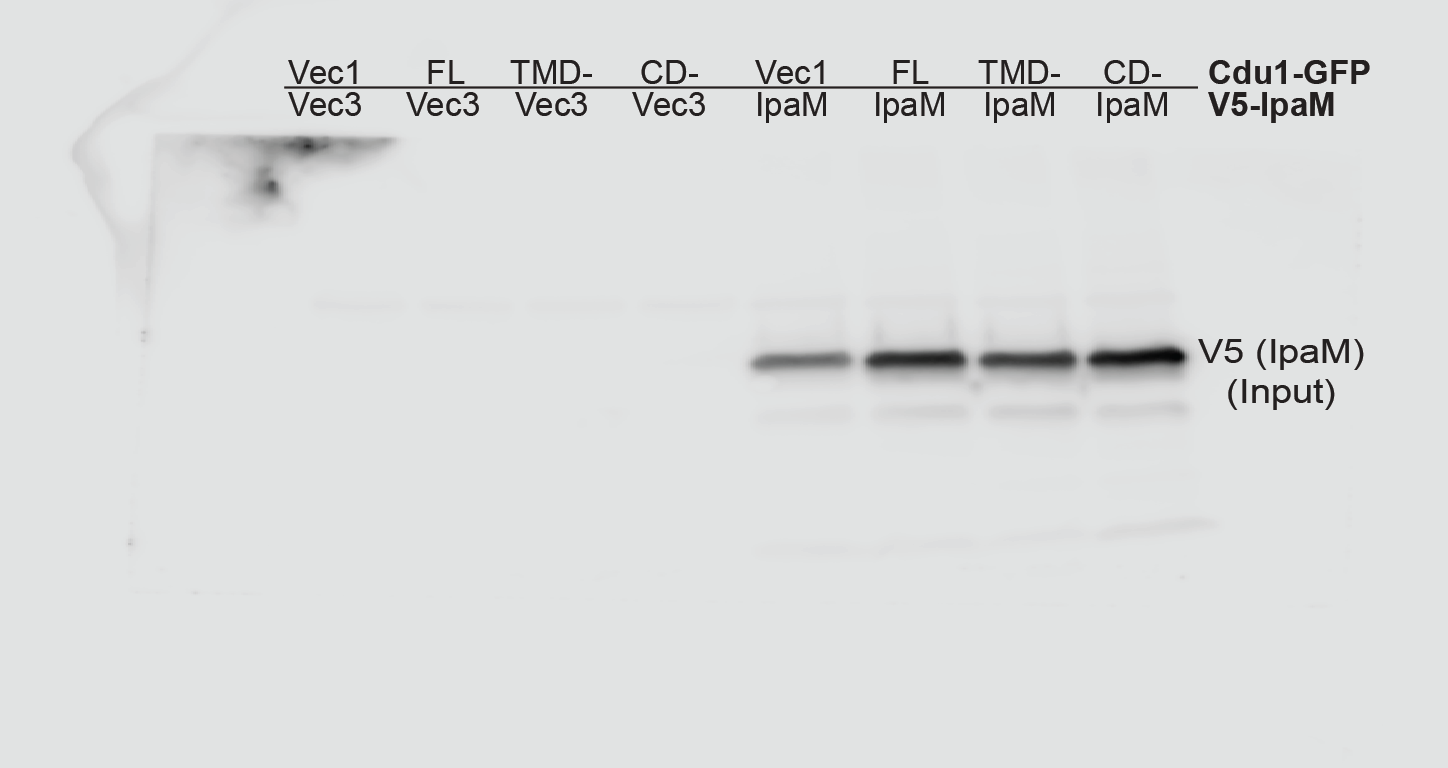

Supplement: Figure 2—source data 5. [file elife-87386-fig2-data5.zip › 11_Figure 2-source data 5/02_Cdu1_IpaM interaction Figure_Raw data_input V5 (IpaM)_Annotated.png]

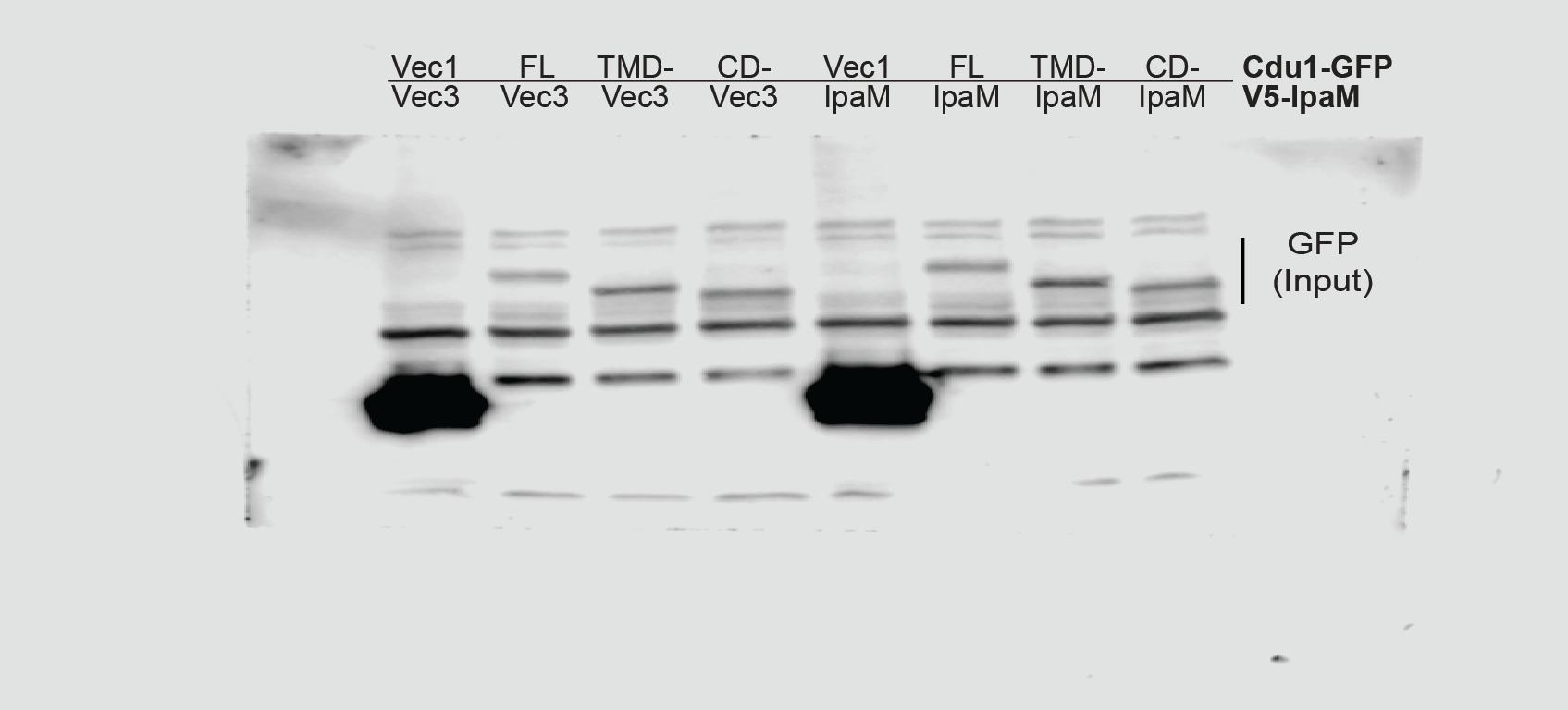

Supplement: Figure 2—source data 5. [file elife-87386-fig2-data5.zip › 11_Figure 2-source data 5/01_Cdu1_IpaM interaction Figure_Raw data_input GFP_Annotated.png]

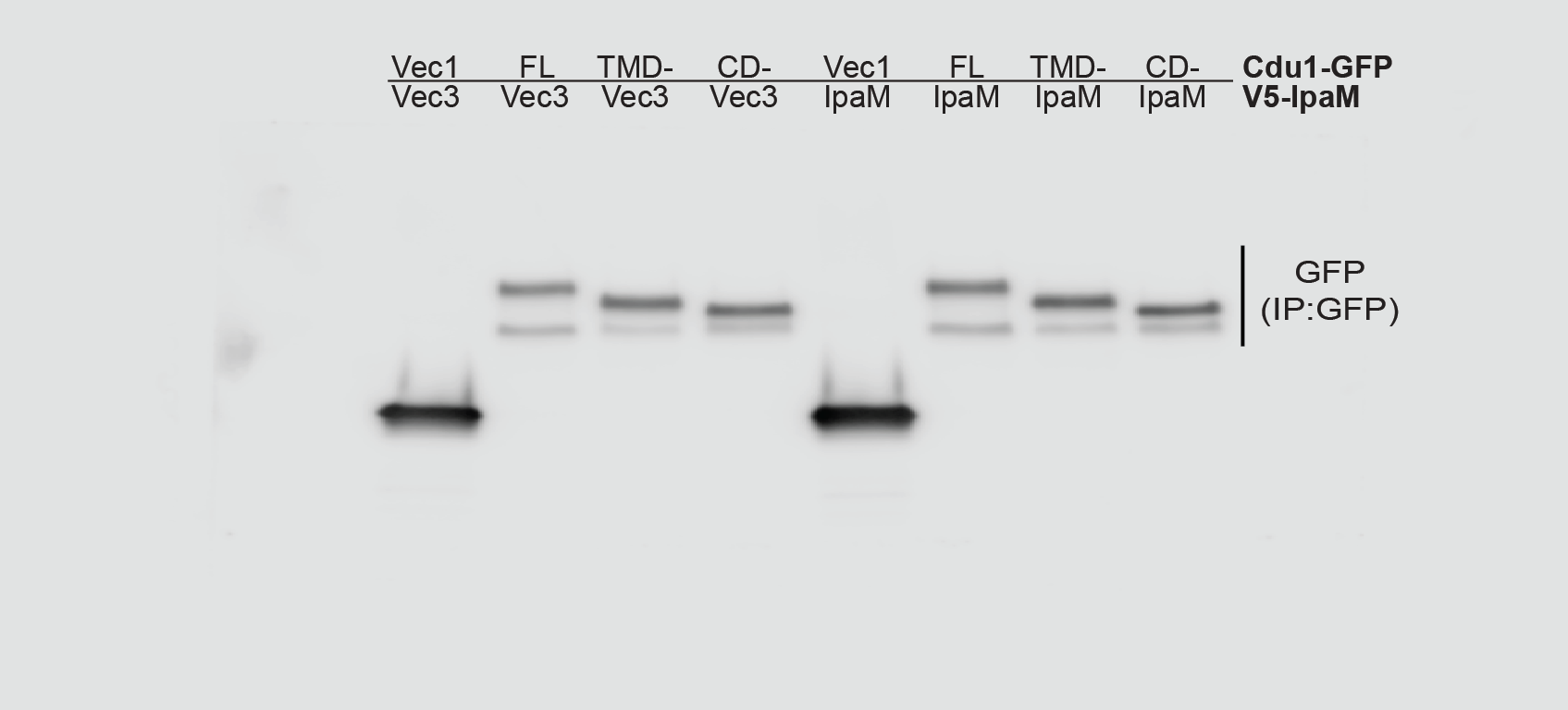

Supplement: Figure 2—source data 5. [file elife-87386-fig2-data5.zip › 11_Figure 2-source data 5/04_Cdu1_IpaM interaction Figure_Raw data_IP GFP_ GFP_Annotated.png]

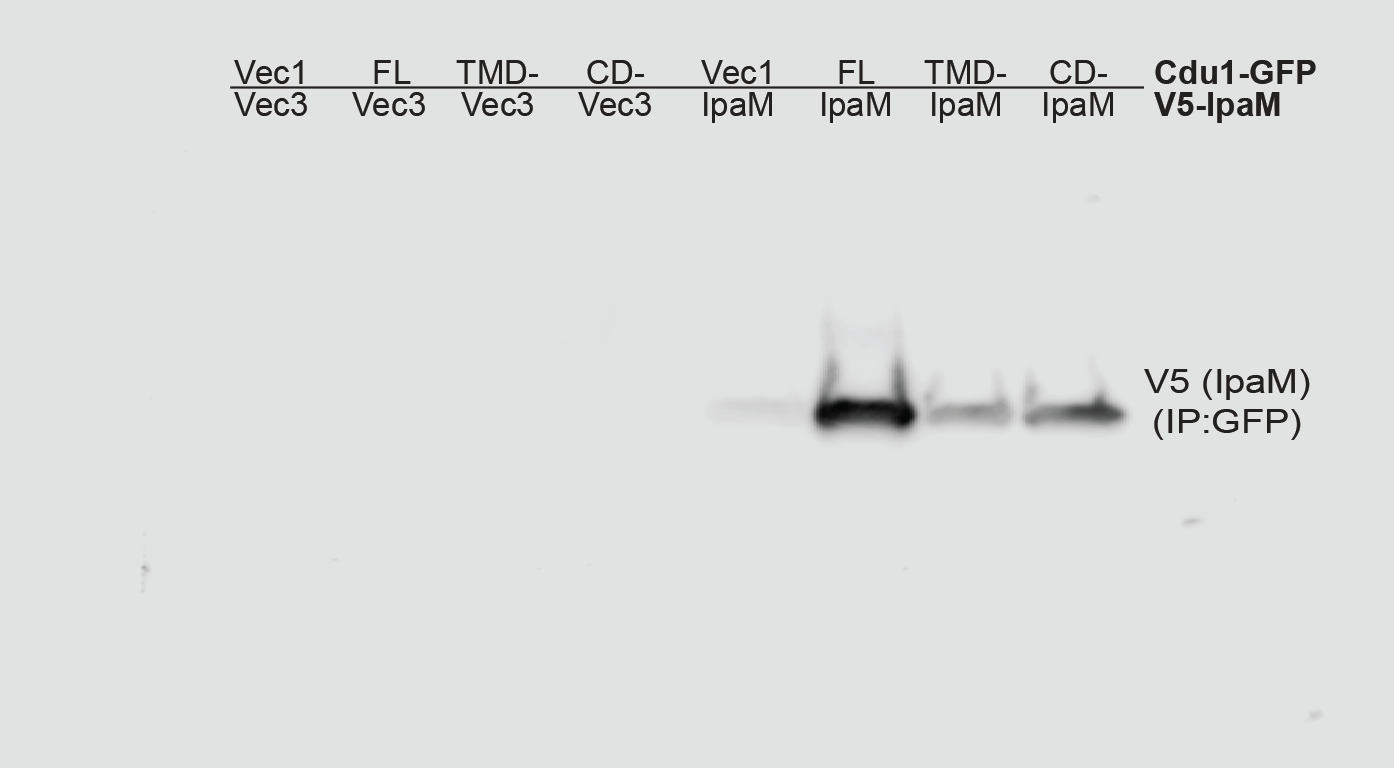

Supplement: Figure 2—source data 5. [file elife-87386-fig2-data5.zip › 11_Figure 2-source data 5/05_Cdu1_IpaM interaction Figure_Raw data_IP GFP_ V5 (IpaM)_Annotated.png]

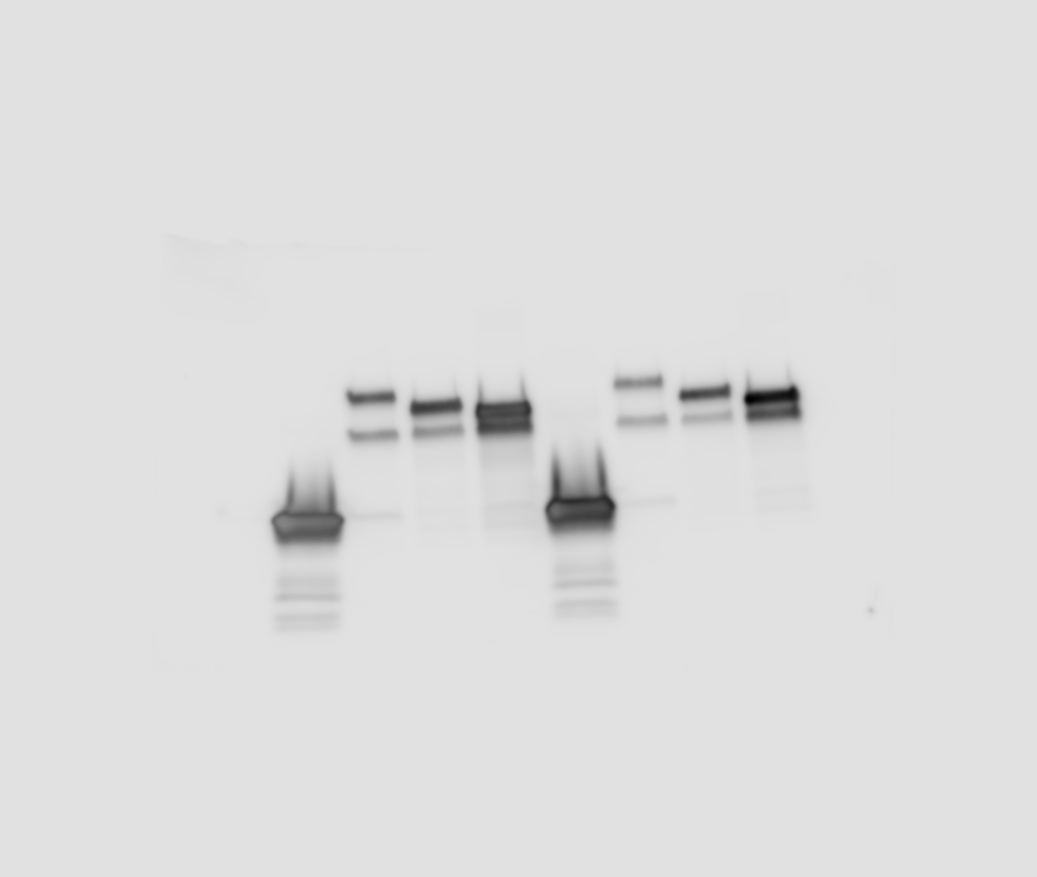

Supplement: Figure 2—source data 6. [file elife-87386-fig2-data6.zip › 12_Figure 2-source data 6/04_Cdu1_CTL0480 interaction Figure_Raw data_IP GFP (Cdu1 variants).tif]

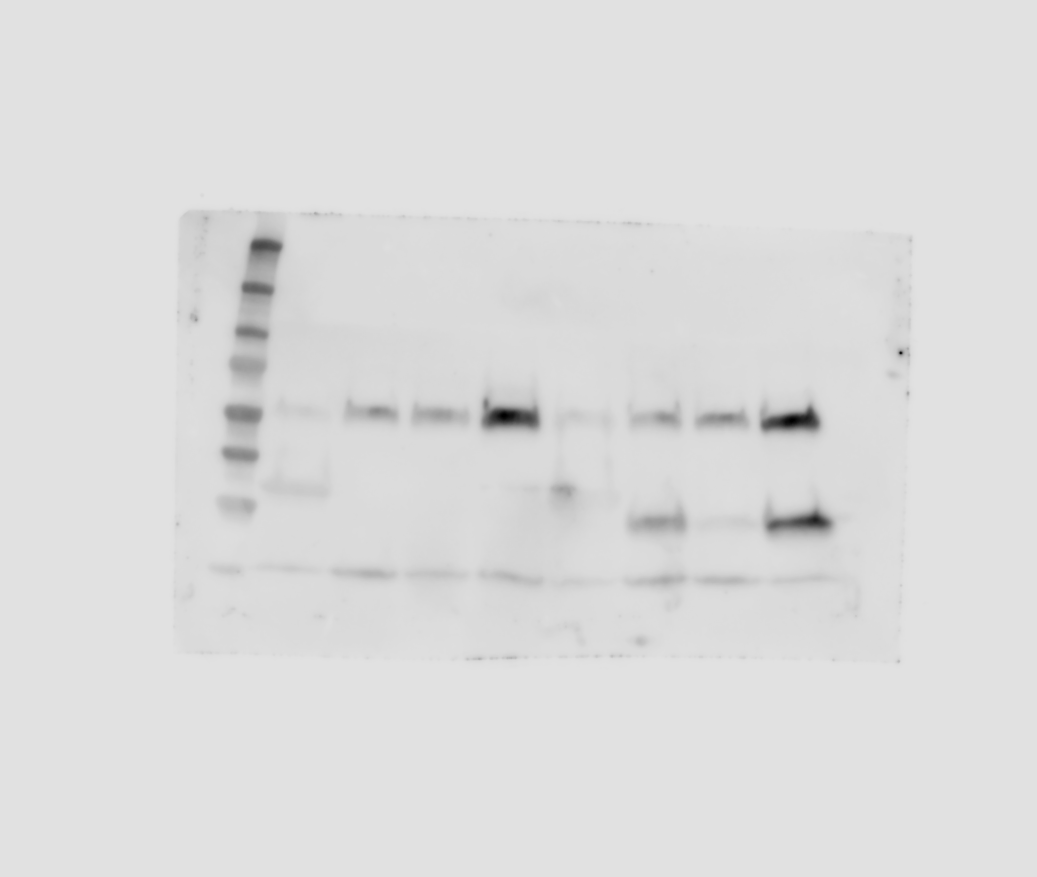

Supplement: Figure 2—source data 6. [file elife-87386-fig2-data6.zip › 12_Figure 2-source data 6/05_Cdu1_CTL0480 interaction Figure_Raw data_IP V5 (CTL0480).tif]

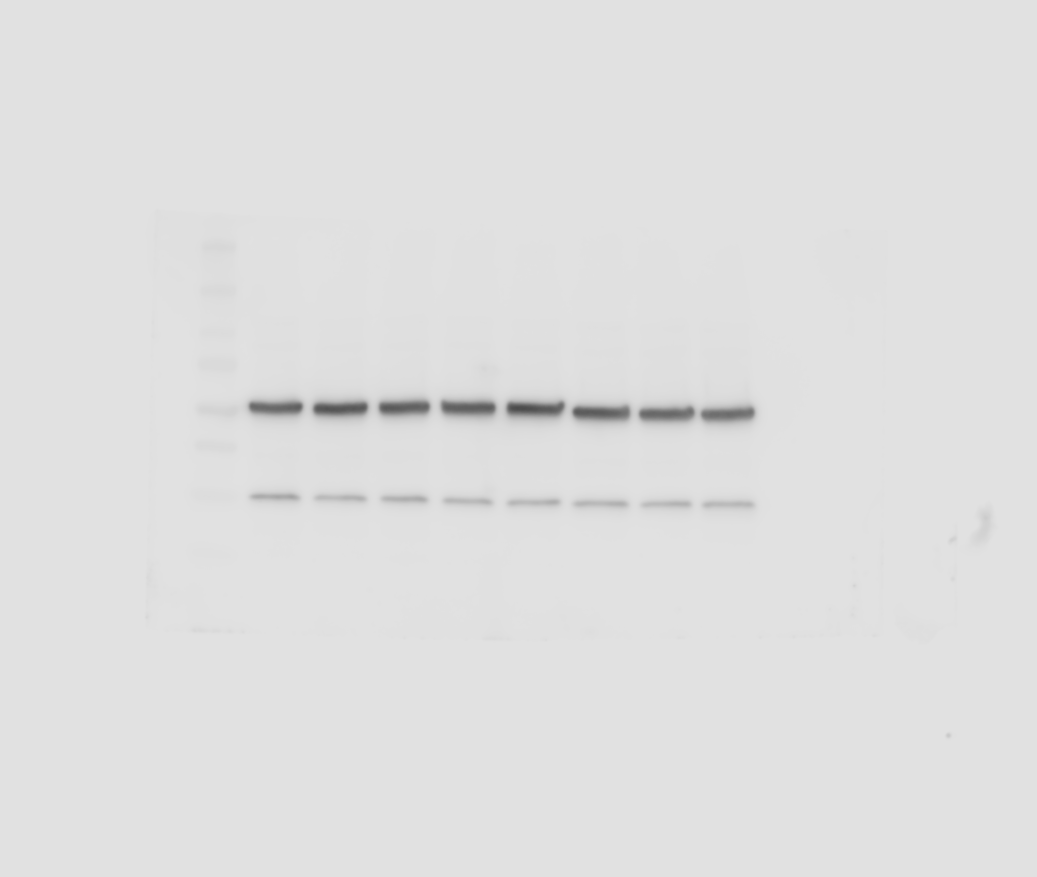

Supplement: Figure 2—source data 6. [file elife-87386-fig2-data6.zip › 12_Figure 2-source data 6/03_Cdu1_CTL0480 interaction Figure_Raw data_input Tubulin.tif]

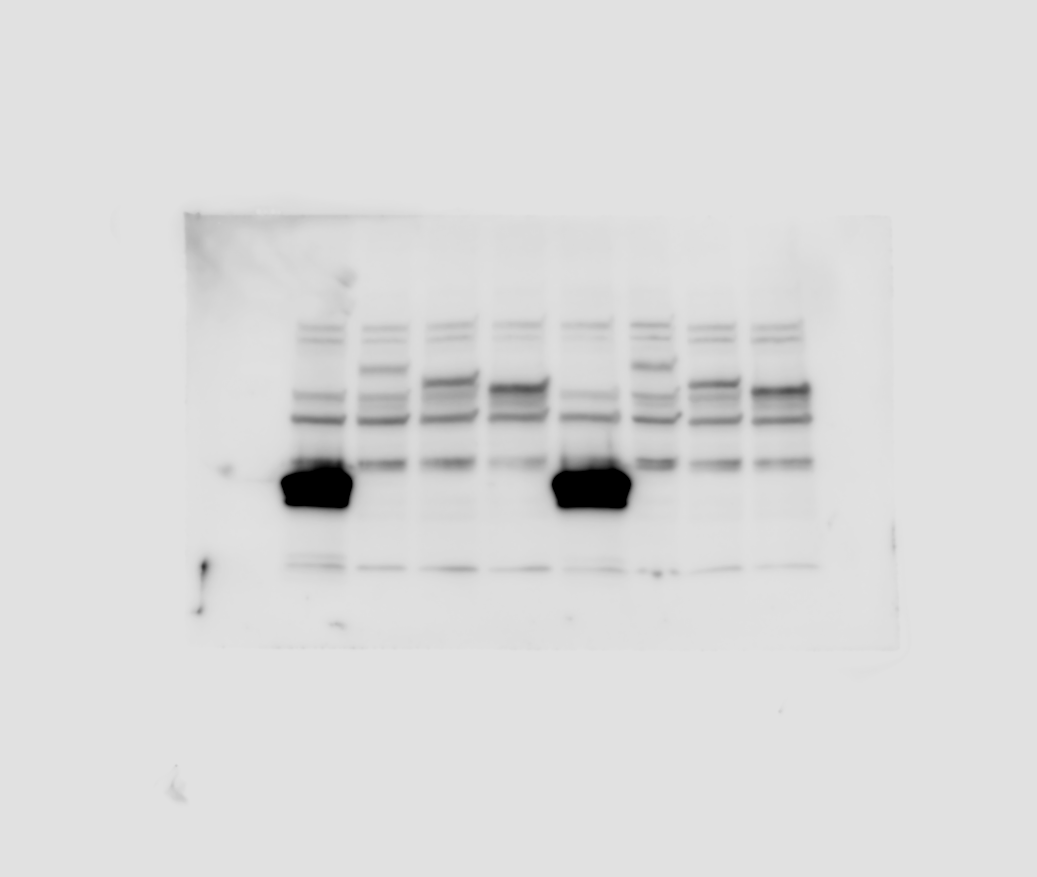

Supplement: Figure 2—source data 6. [file elife-87386-fig2-data6.zip › 12_Figure 2-source data 6/01_Cdu1_CTL0480 interaction Figure_Raw data_input GFP.tif]

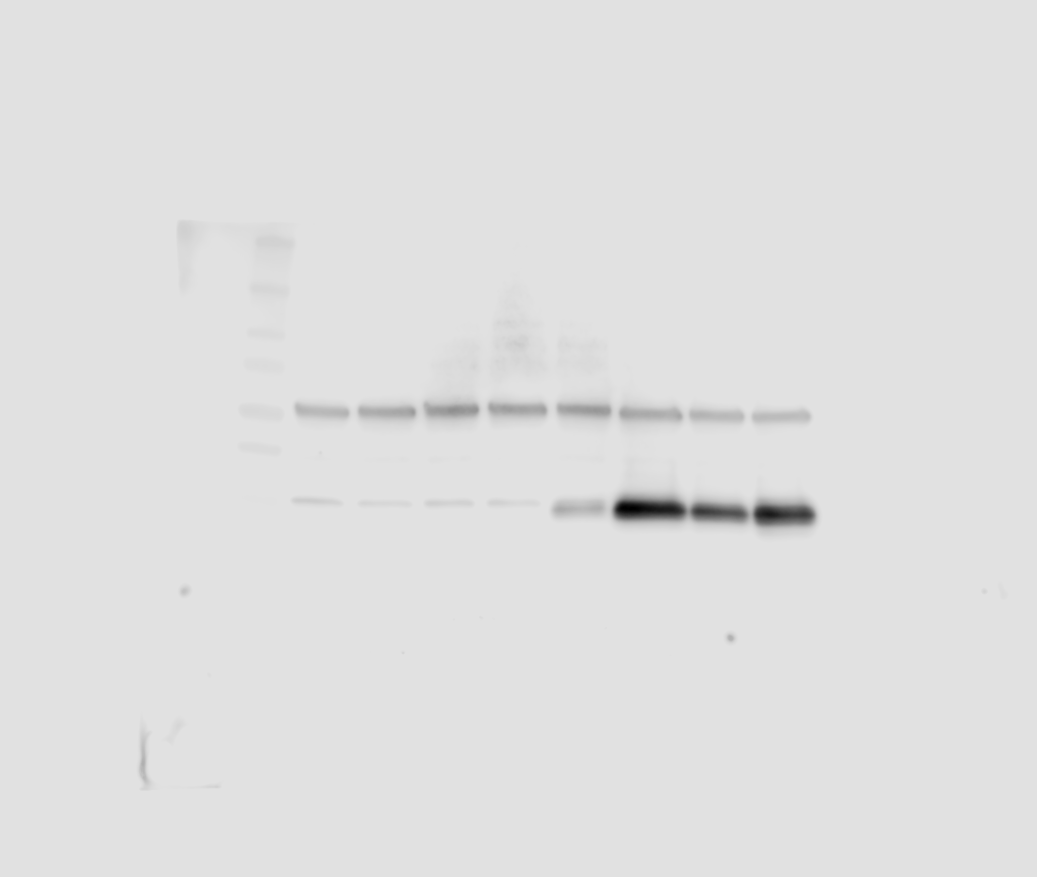

Supplement: Figure 2—source data 6. [file elife-87386-fig2-data6.zip › 12_Figure 2-source data 6/02_Cdu1_CTL0480 interaction Figure_Raw data_input V5 (CTL0480).tif]

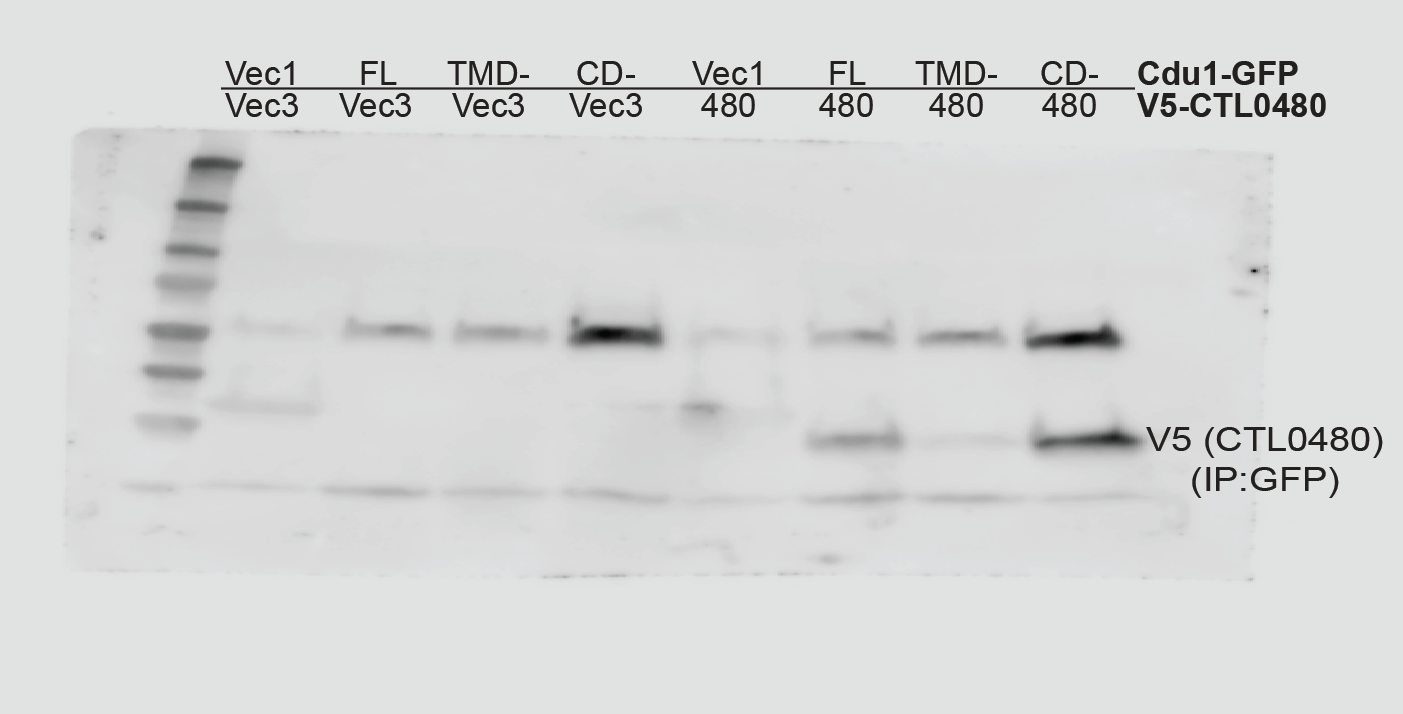

Supplement: Figure 2—source data 7. [file elife-87386-fig2-data7.zip › 13_Figure 2-source data 7/05_Cdu1_CTL0480 interaction Figure_Raw data_IP V5 (CTL0480)_Annotated.png]

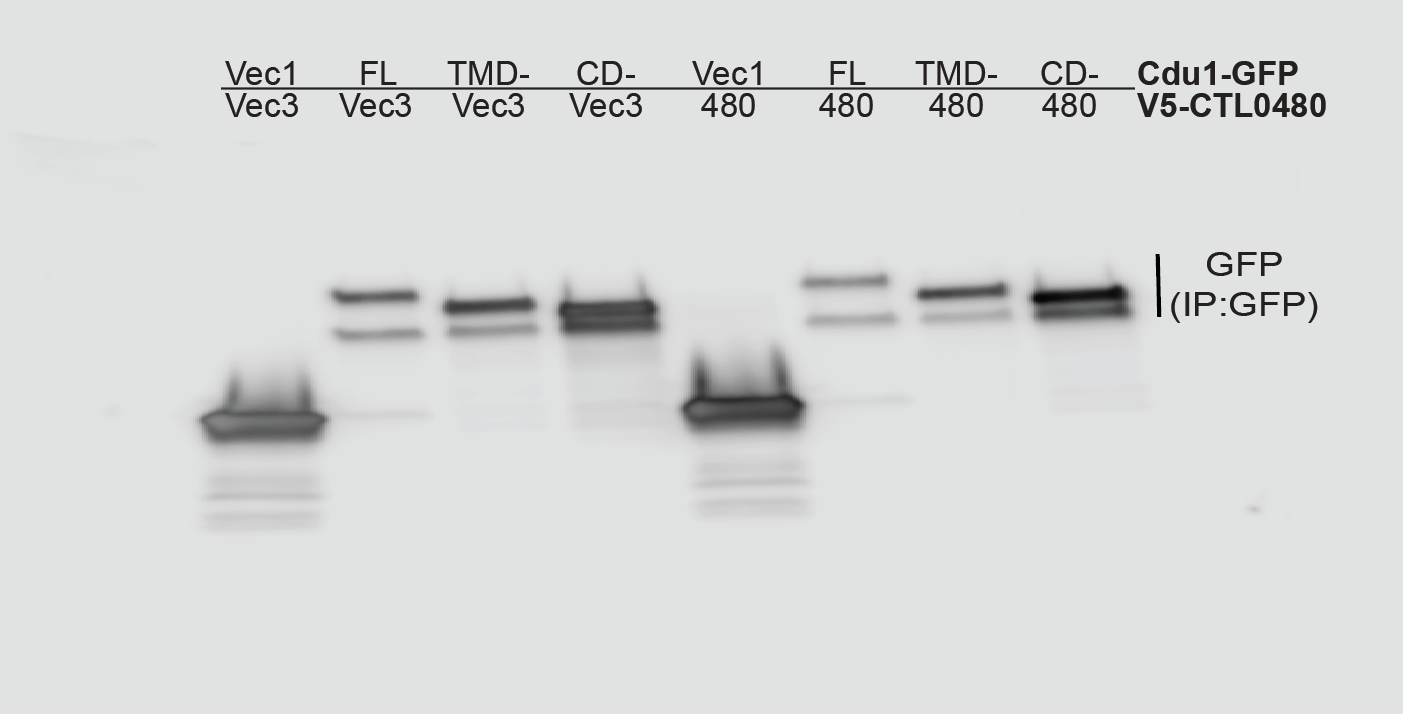

Supplement: Figure 2—source data 7. [file elife-87386-fig2-data7.zip › 13_Figure 2-source data 7/04_Cdu1_CTL0480 interaction Figure_Raw data_IP GFP (Cdu1 variants).png]

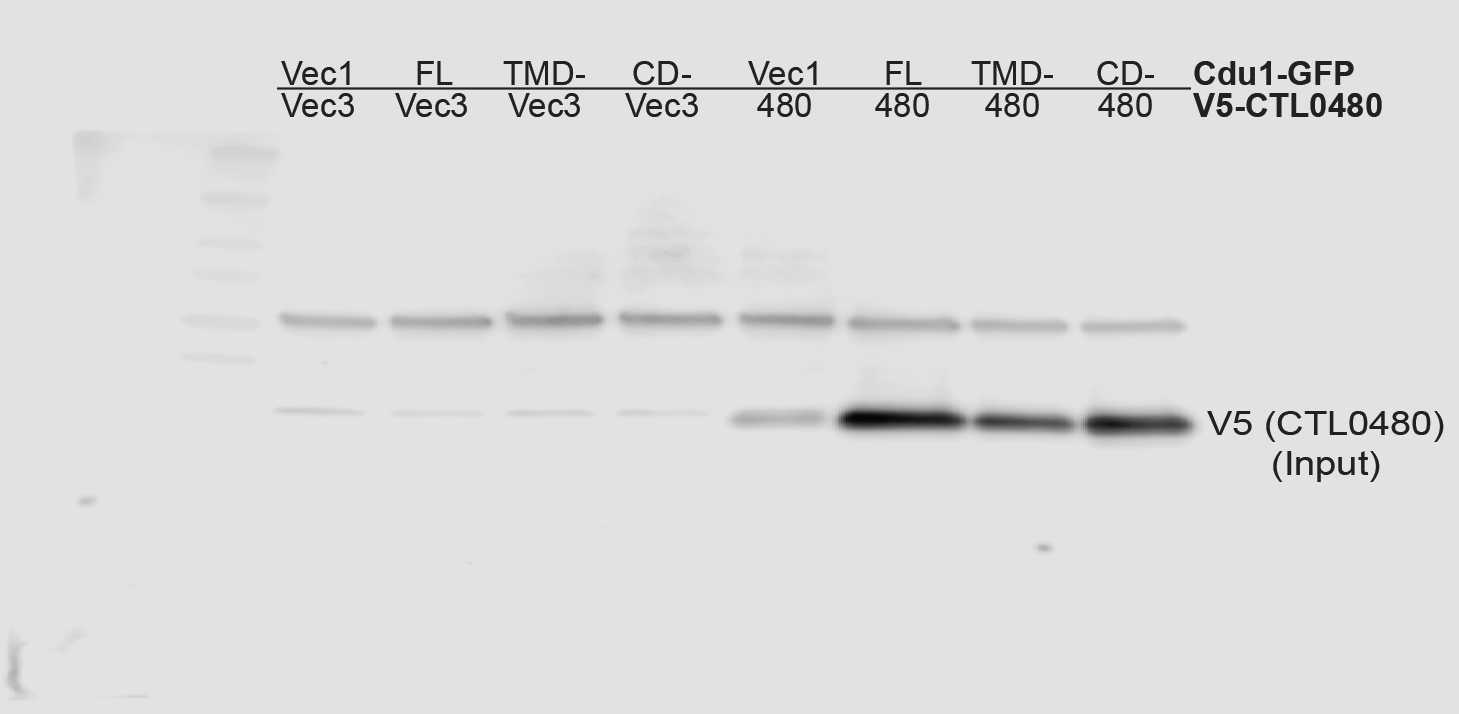

Supplement: Figure 2—source data 7. [file elife-87386-fig2-data7.zip › 13_Figure 2-source data 7/02_Cdu1_CTL0480 interaction Figure_Raw data_input V5 (CTL0480)_Annotated.png]

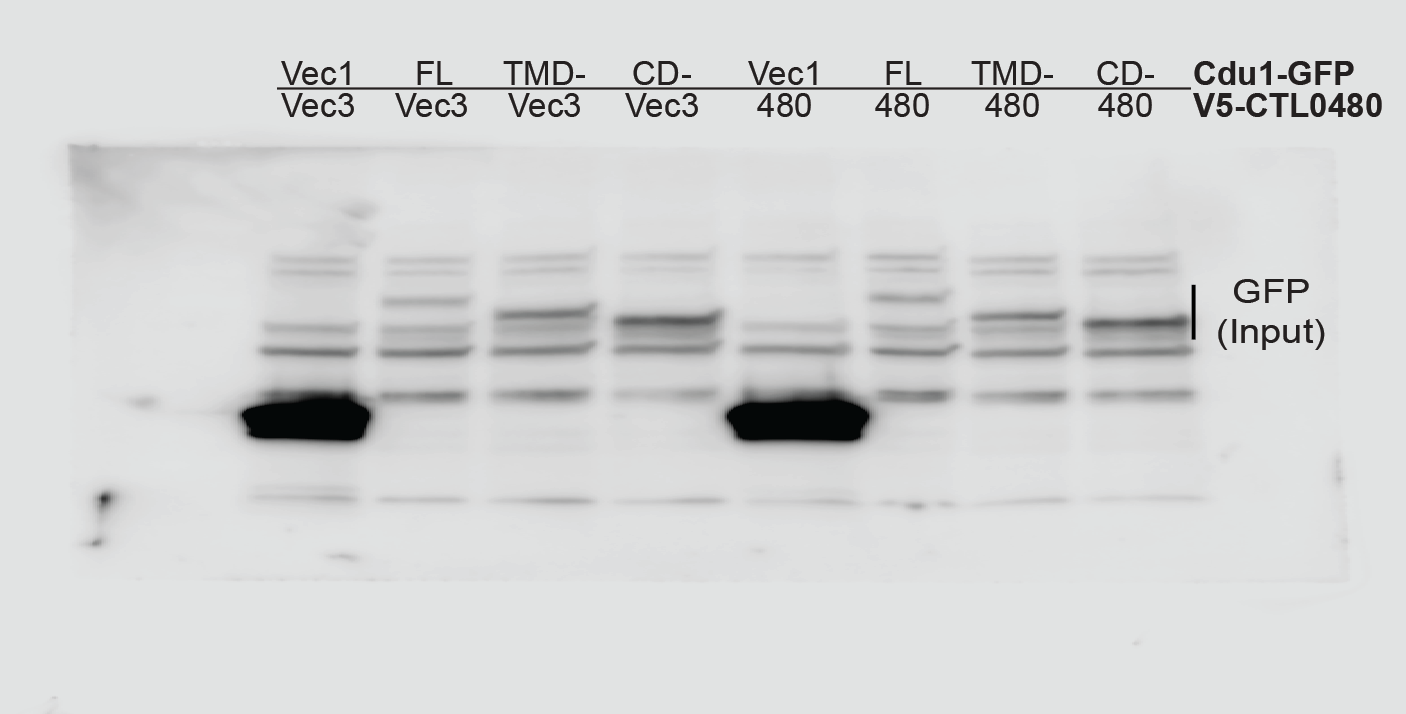

Supplement: Figure 2—source data 7. [file elife-87386-fig2-data7.zip › 13_Figure 2-source data 7/01_Cdu1_CTL0480 interaction Figure_Raw data_input GFP_Annotated.png]

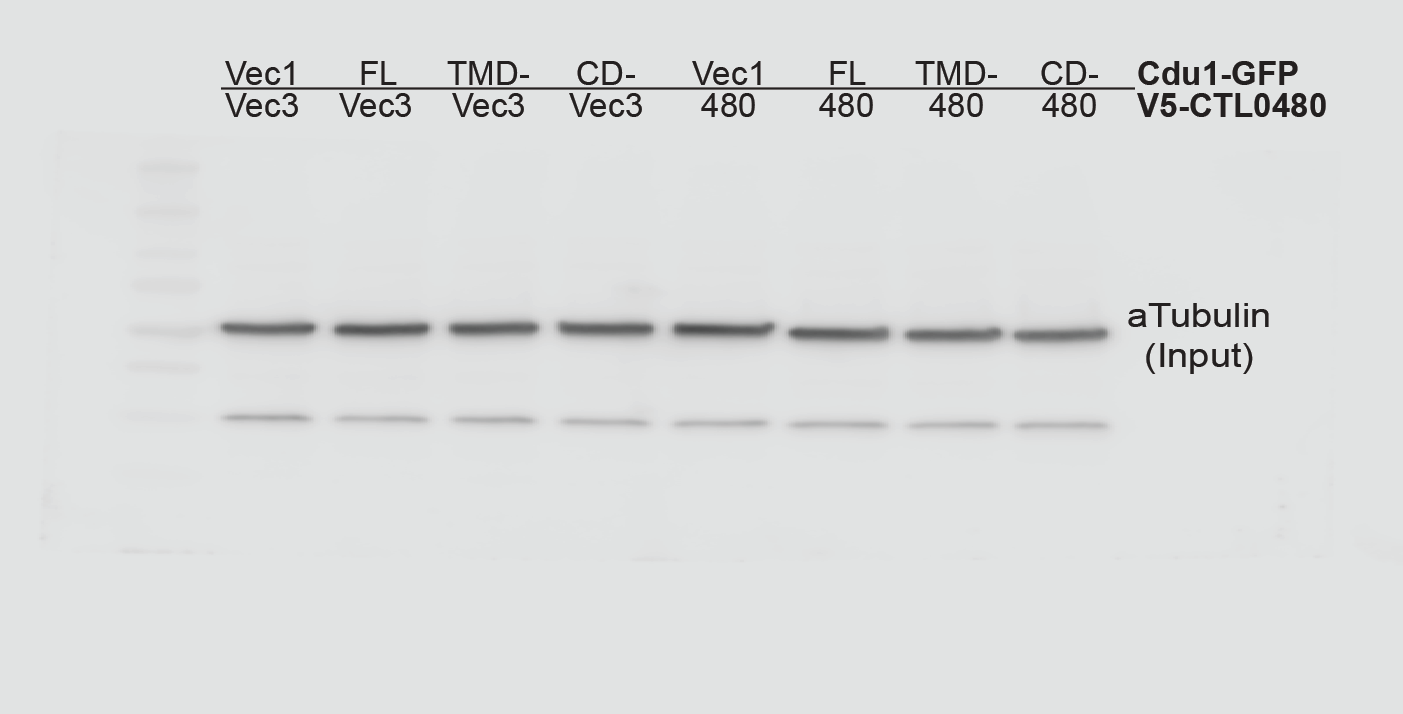

Supplement: Figure 2—source data 7. [file elife-87386-fig2-data7.zip › 13_Figure 2-source data 7/03_Cdu1_CTL0480 interaction Figure_Raw data_input Tubulin_Annotated.png]

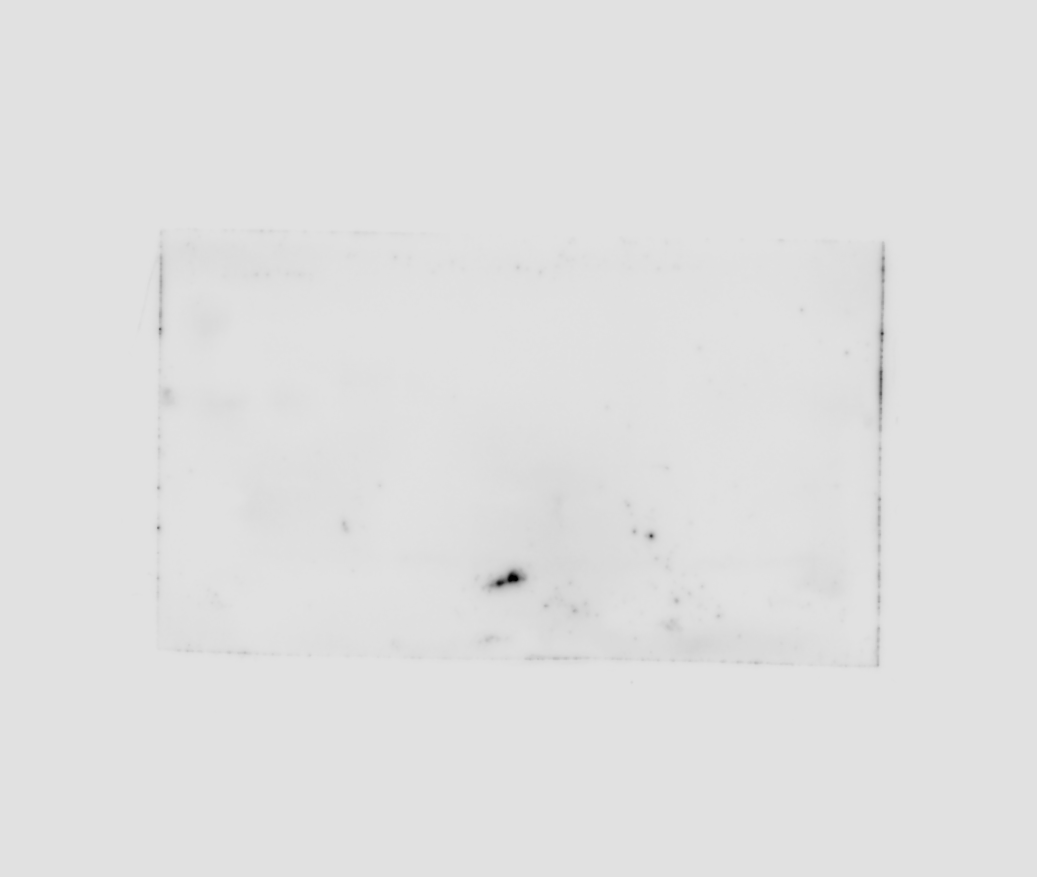

Supplement: Figure 2—source data 8. [file elife-87386-fig2-data8.zip › 14_Figure 2-source data 8/05_Cdu1_CpoS interaction Figure_Raw data_IP V5 (CpoS).tif]

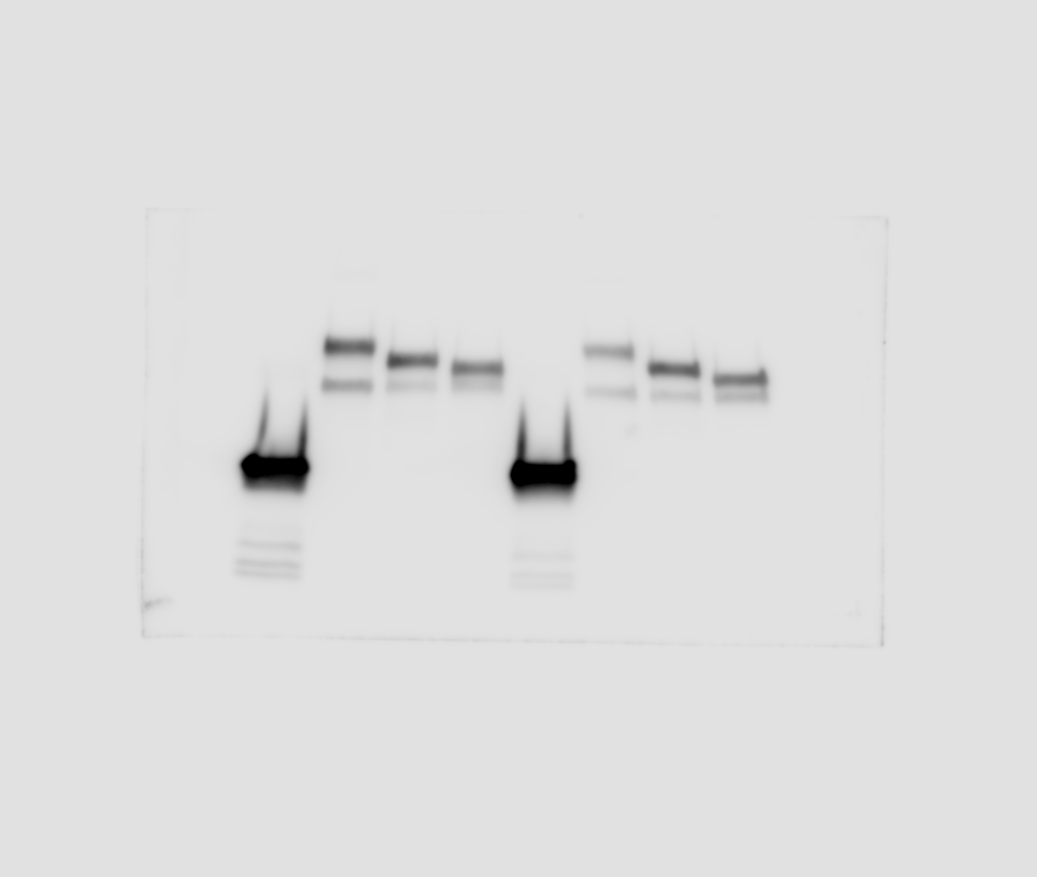

Supplement: Figure 2—source data 8. [file elife-87386-fig2-data8.zip › 14_Figure 2-source data 8/01_Cdu1_CpoS interaction Figure_Raw data_input GFP.tif]

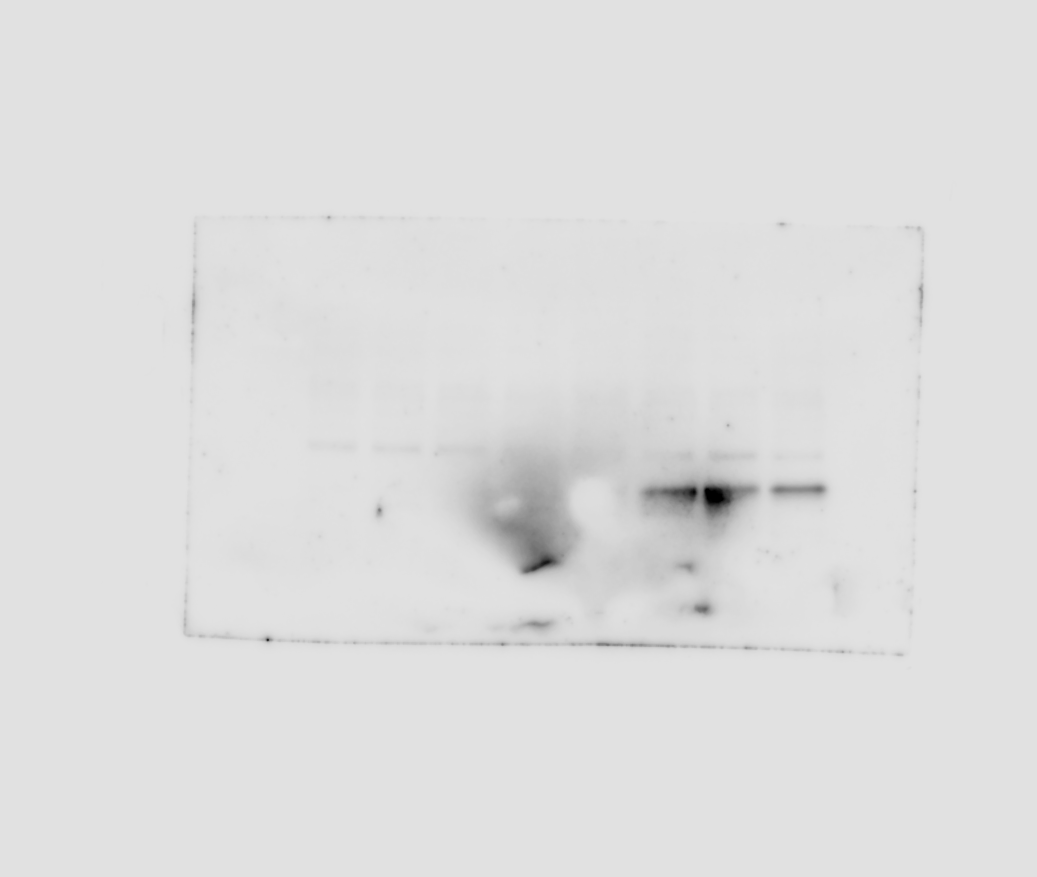

Supplement: Figure 2—source data 8. [file elife-87386-fig2-data8.zip › 14_Figure 2-source data 8/02_Cdu1_CpoS interaction Figure_Raw data_input V5 (CpoS).tif]

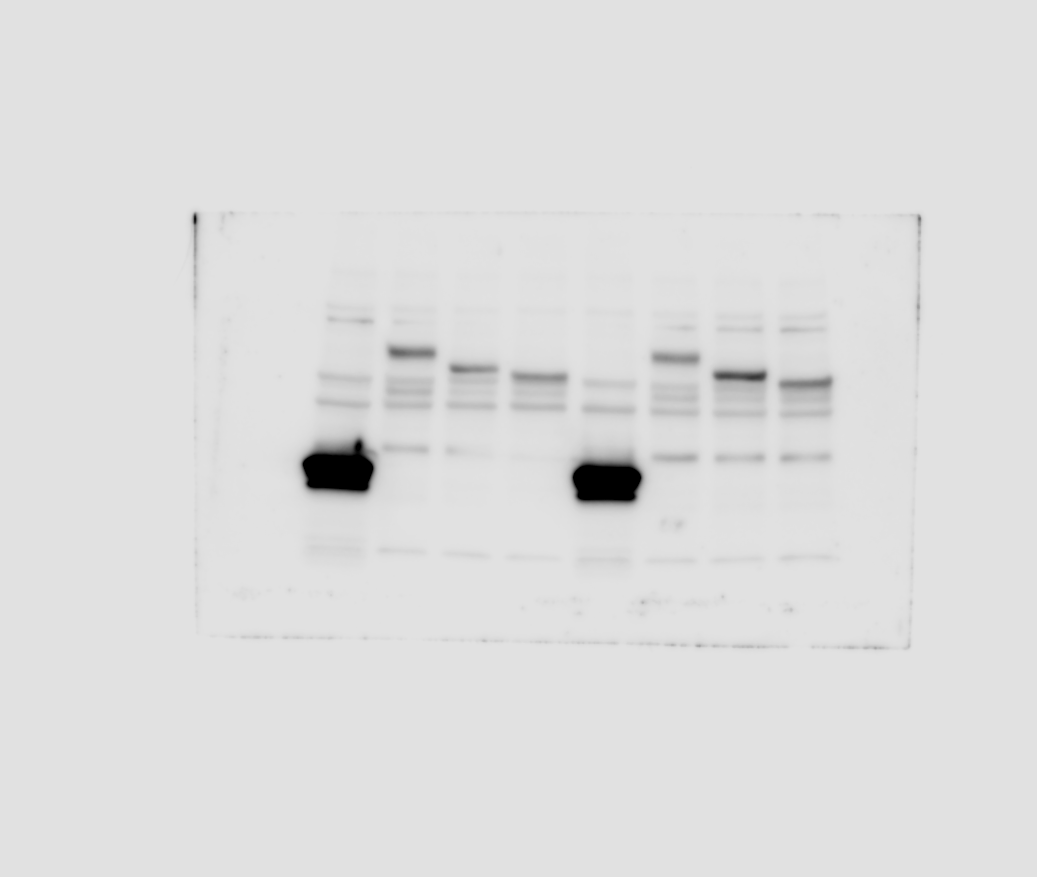

Supplement: Figure 2—source data 8. [file elife-87386-fig2-data8.zip › 14_Figure 2-source data 8/04_Cdu1_CpoS interaction Figure_Raw data_IP GFP (Cdu1 variants).tif]

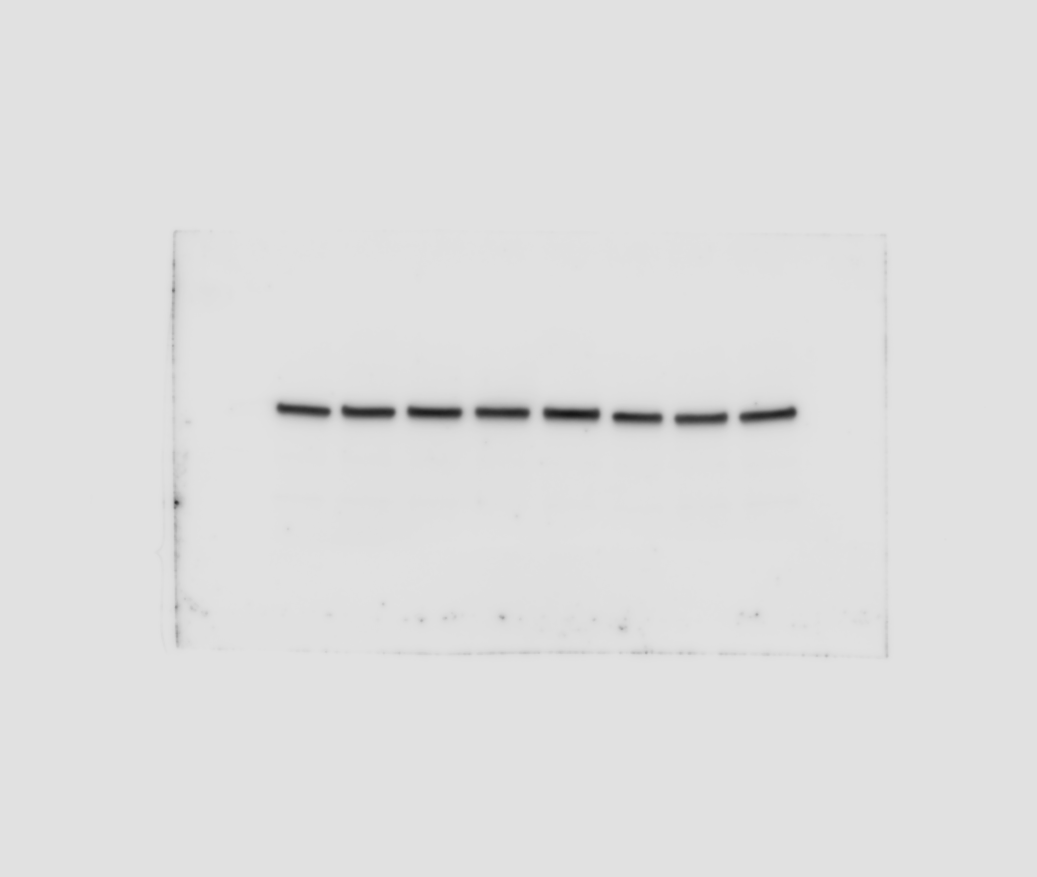

Supplement: Figure 2—source data 8. [file elife-87386-fig2-data8.zip › 14_Figure 2-source data 8/03_Cdu1_CpoS interaction Figure_Raw data_input Tubulin.tif]

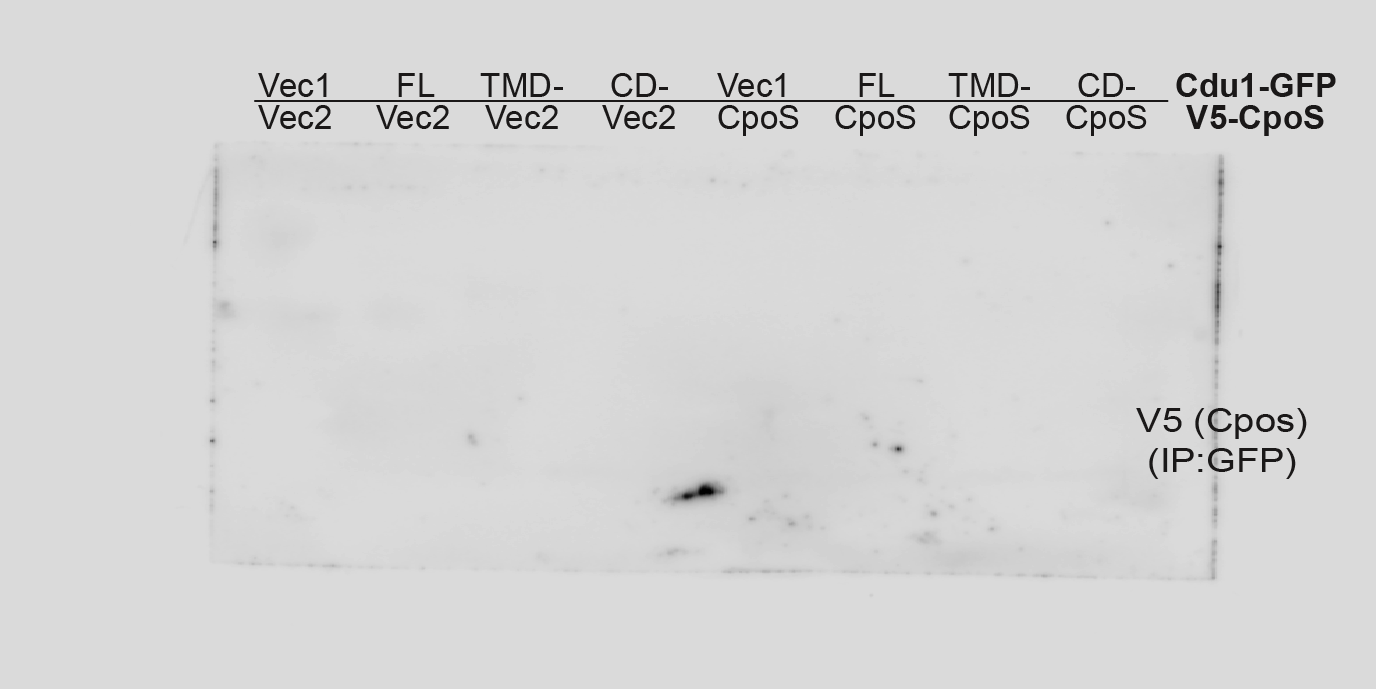

Supplement: Figure 2—source data 9. [file elife-87386-fig2-data9.zip › 15_Figure 2-source data 9/05_Cdu1_CpoS interaction Figure_Raw data_IP V5 (CpoS)_Annotated.png]

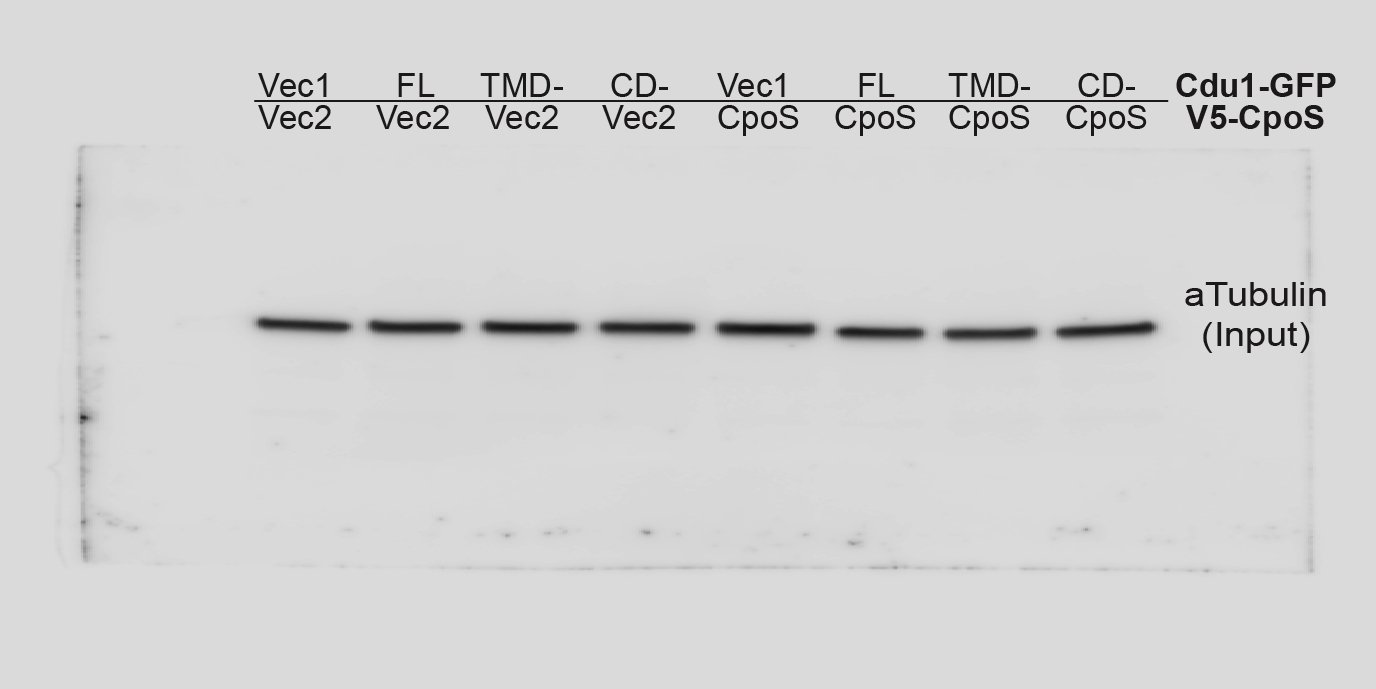

Supplement: Figure 2—source data 9. [file elife-87386-fig2-data9.zip › 15_Figure 2-source data 9/03_Cdu1_CpoS interaction Figure_Raw data_input Tubulin_Annotated.png]

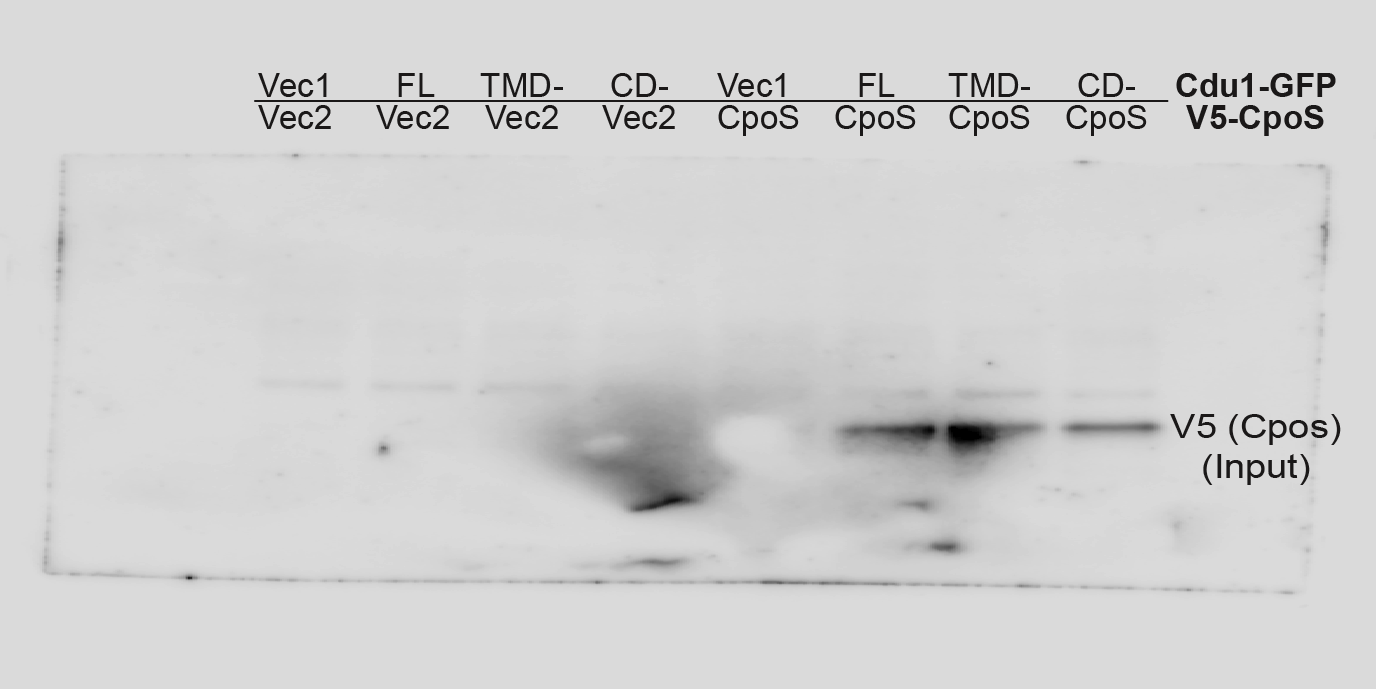

Supplement: Figure 2—source data 9. [file elife-87386-fig2-data9.zip › 15_Figure 2-source data 9/02_Cdu1_CpoS interaction Figure_Raw data_input V5 (CpoS)_Annotated.png]

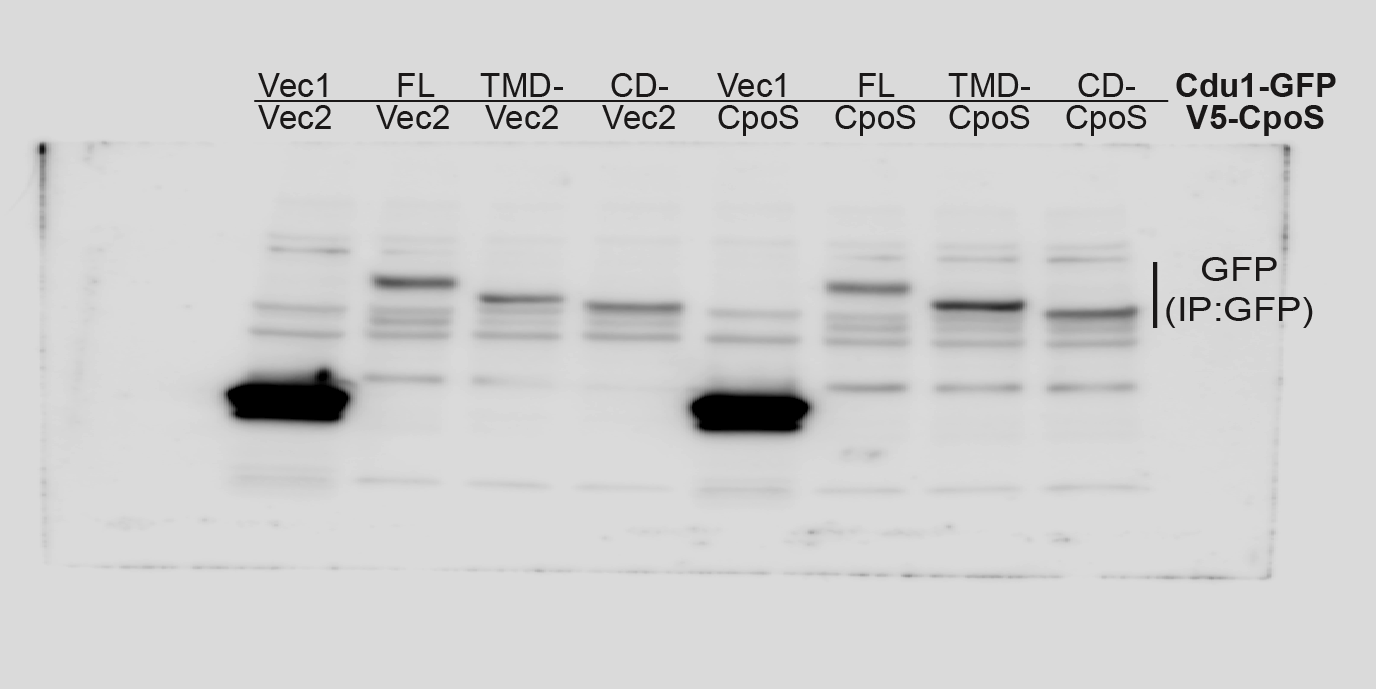

Supplement: Figure 2—source data 9. [file elife-87386-fig2-data9.zip › 15_Figure 2-source data 9/04_Cdu1_CpoS interaction Figure_Raw data_IP GFP (Cdu1 variants)_Annotated.png]

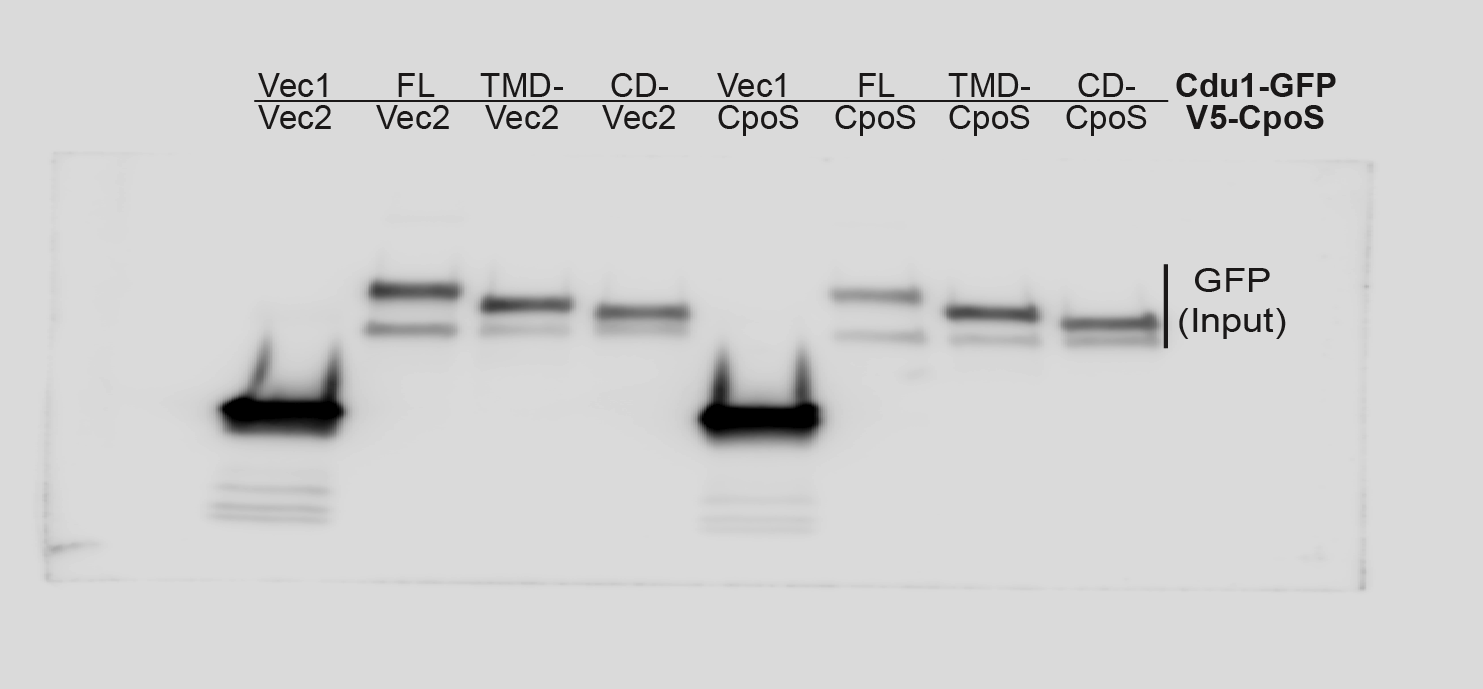

Supplement: Figure 2—source data 9. [file elife-87386-fig2-data9.zip › 15_Figure 2-source data 9/01_Cdu1_CpoS interaction Figure_Raw data_input GFP_Annotated.png]

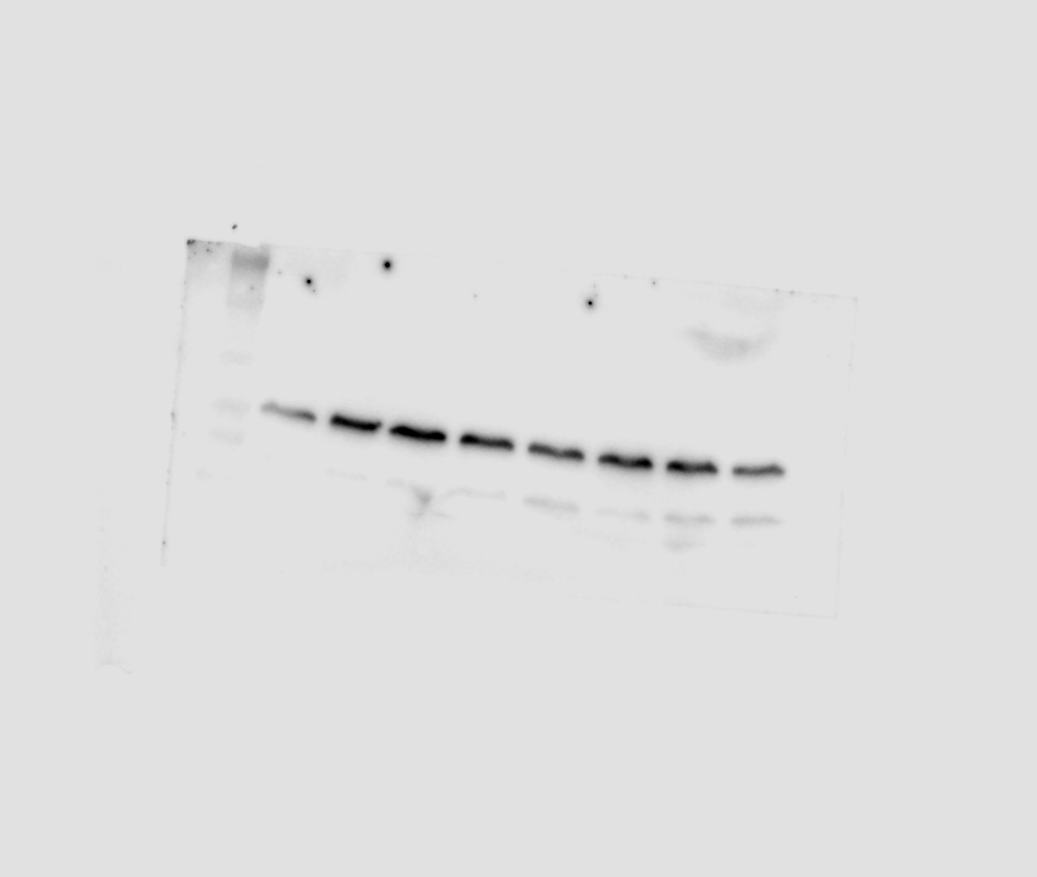

Supplement: Figure 3—source data 1. [file elife-87386-fig3-data1.zip › 16_Figure 3-source data 1/alpha Tubulin WB.png]

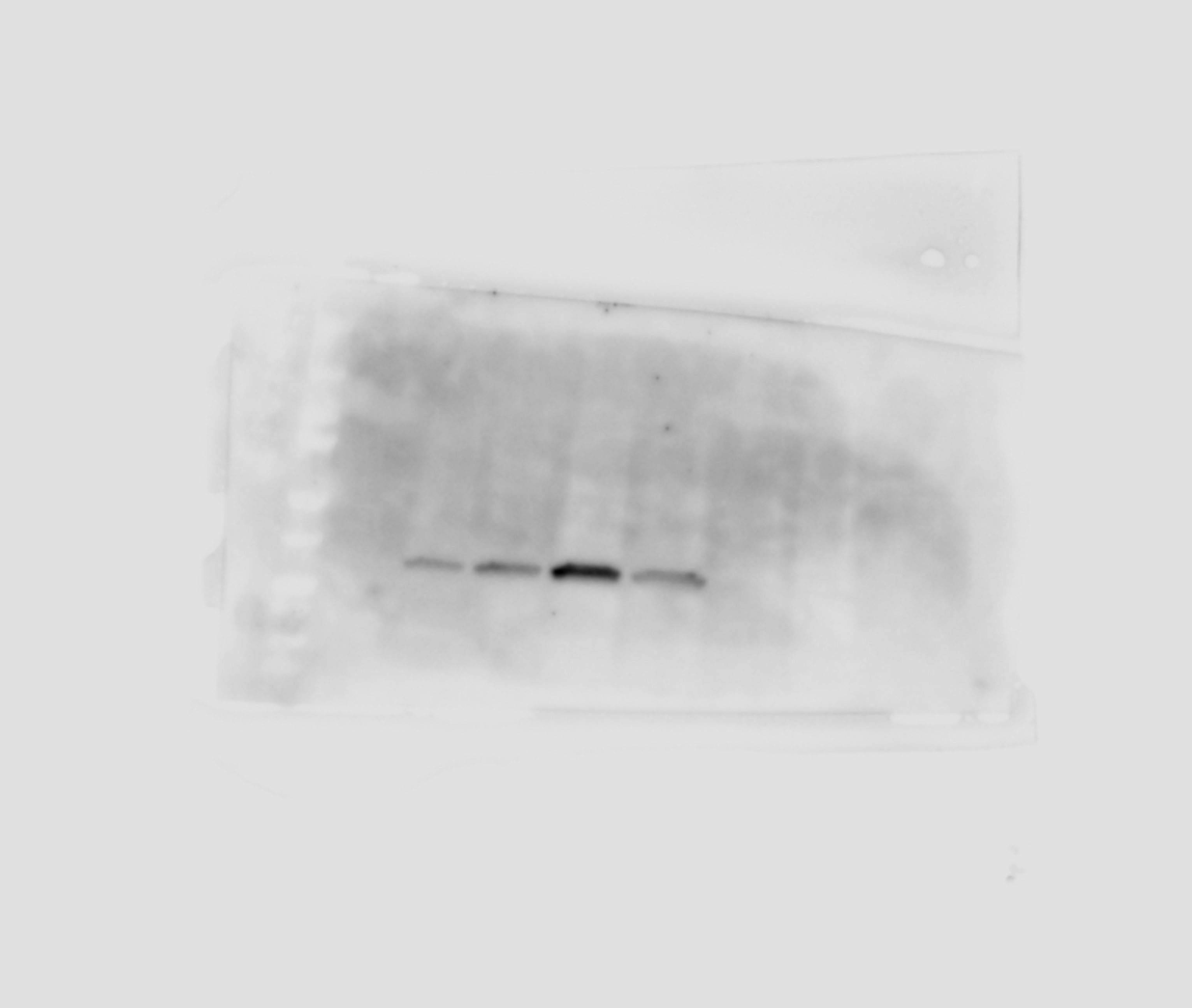

Supplement: Figure 3—source data 1. [file elife-87386-fig3-data1.zip › 16_Figure 3-source data 1/InaC WB.tif]

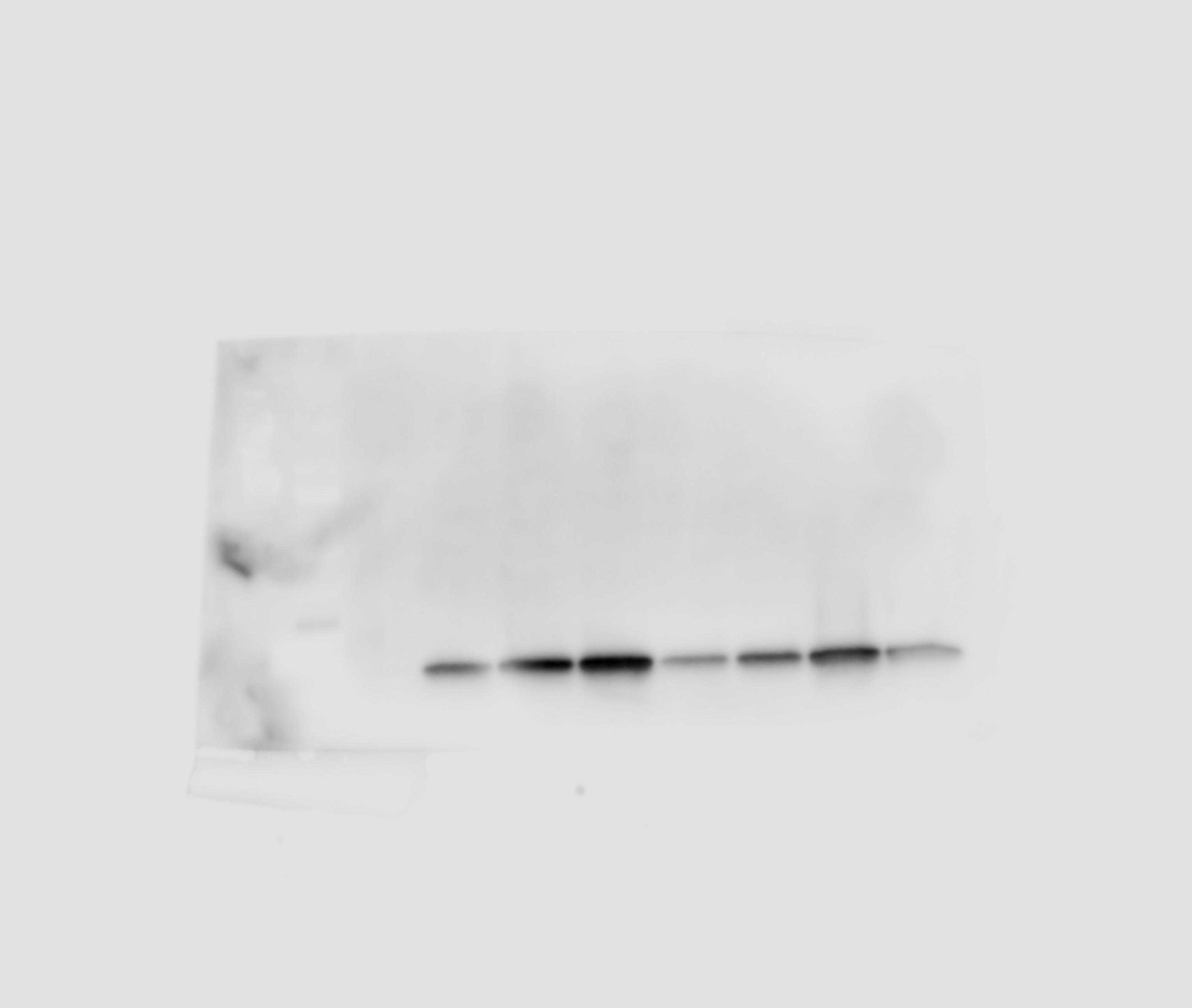

Supplement: Figure 3—source data 1. [file elife-87386-fig3-data1.zip › 16_Figure 3-source data 1/Slc1 WB.tif]

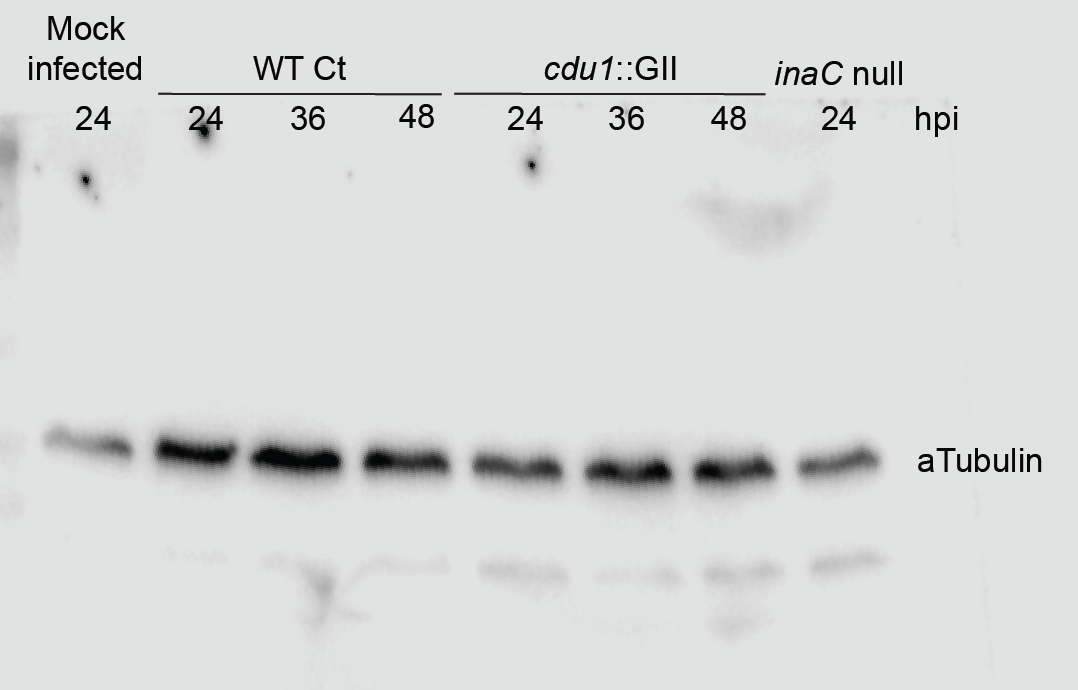

Supplement: Figure 3—source data 2. [file elife-87386-fig3-data2.zip › 17_Figure 3-source data 2/alpha Tubulin WB_Annotated.png]

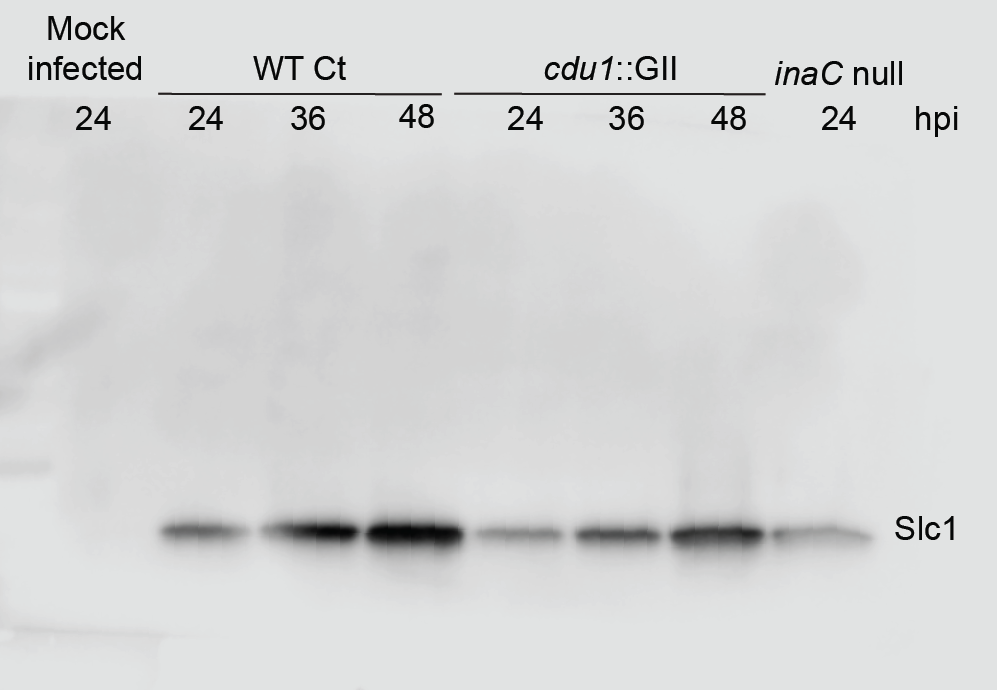

Supplement: Figure 3—source data 2. [file elife-87386-fig3-data2.zip › 17_Figure 3-source data 2/Slc1 WB_Annotated.png]

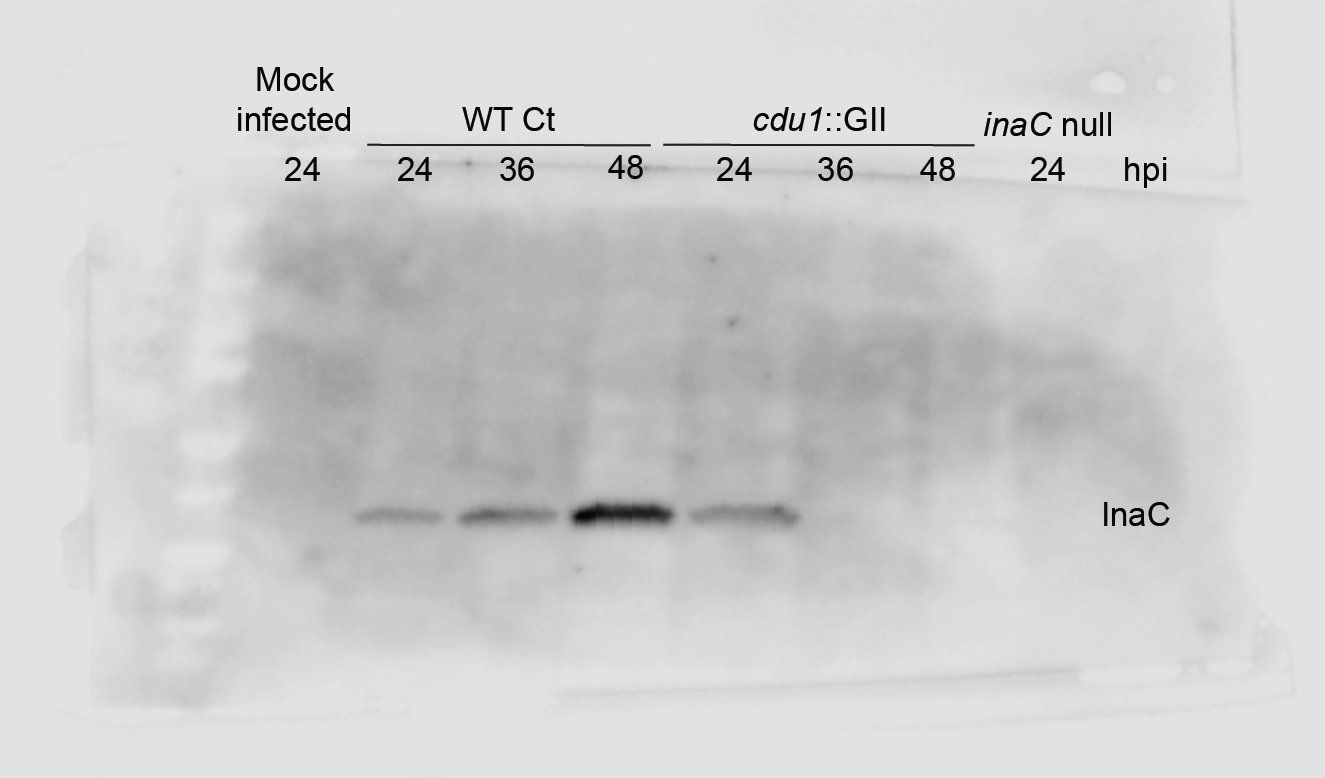

Supplement: Figure 3—source data 2. [file elife-87386-fig3-data2.zip › 17_Figure 3-source data 2/InaC WB_Annotated.png]

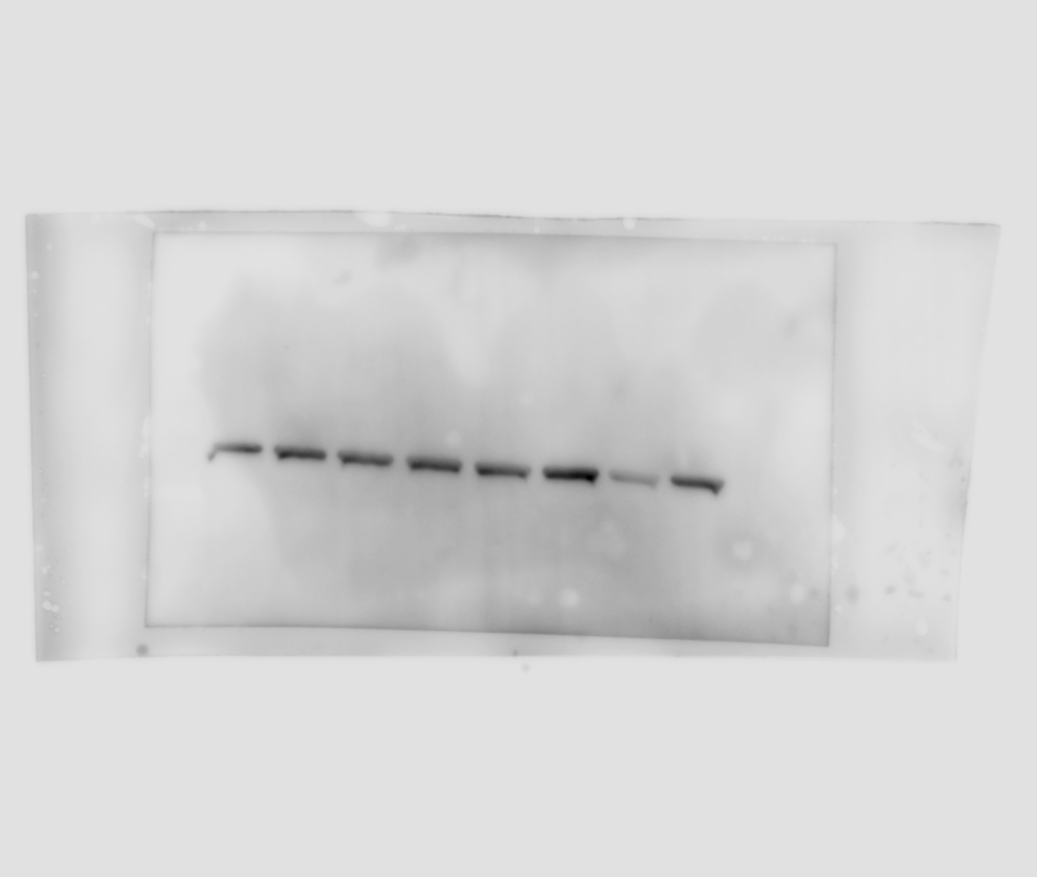

Supplement: Figure 3—source data 4. [file elife-87386-fig3-data4.zip › 19_Figure 3-source data 4/aTubulin WB.png]

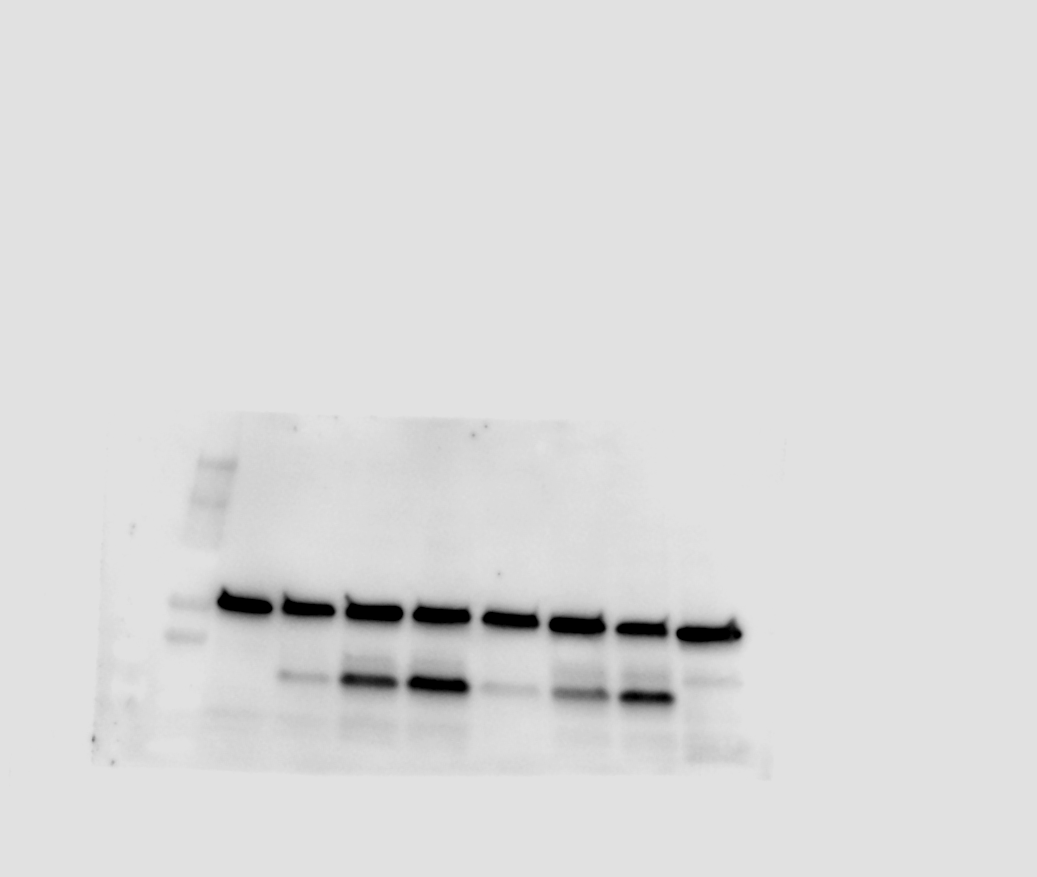

Supplement: Figure 3—source data 4. [file elife-87386-fig3-data4.zip › 19_Figure 3-source data 4/IPAM WB.tif]

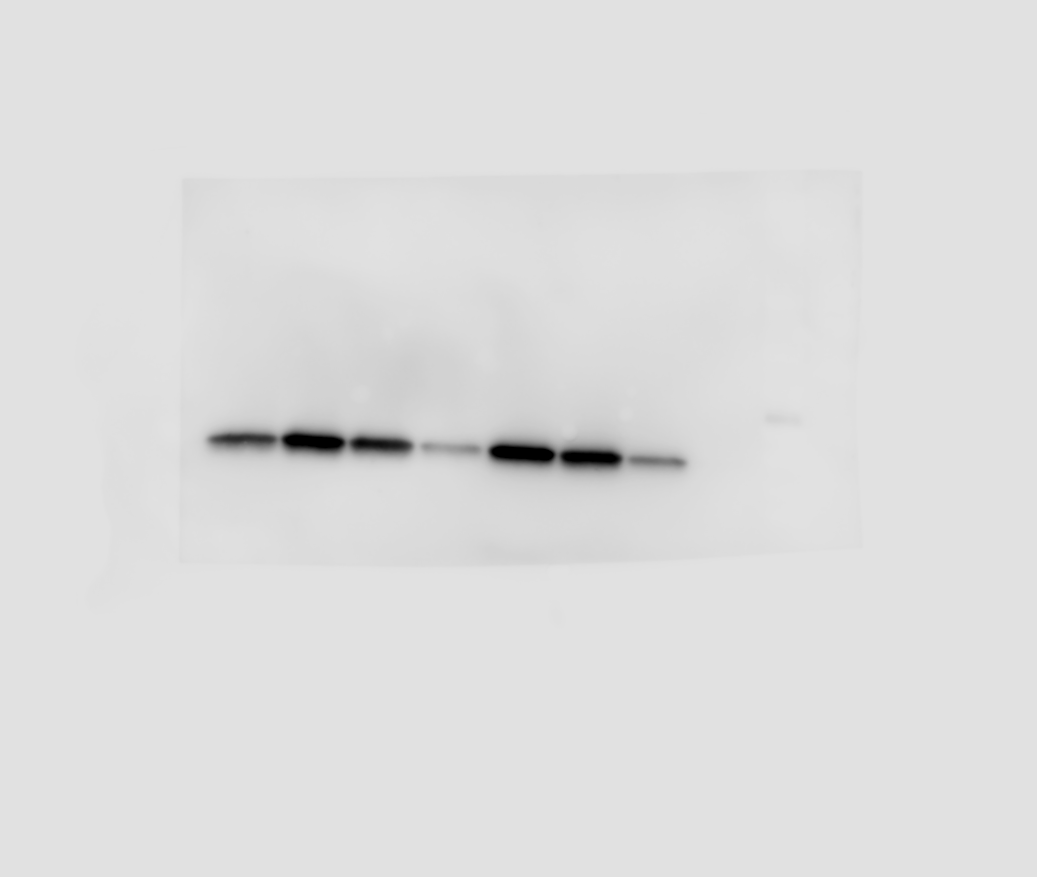

Supplement: Figure 3—source data 4. [file elife-87386-fig3-data4.zip › 19_Figure 3-source data 4/Slc1 WB.tif]

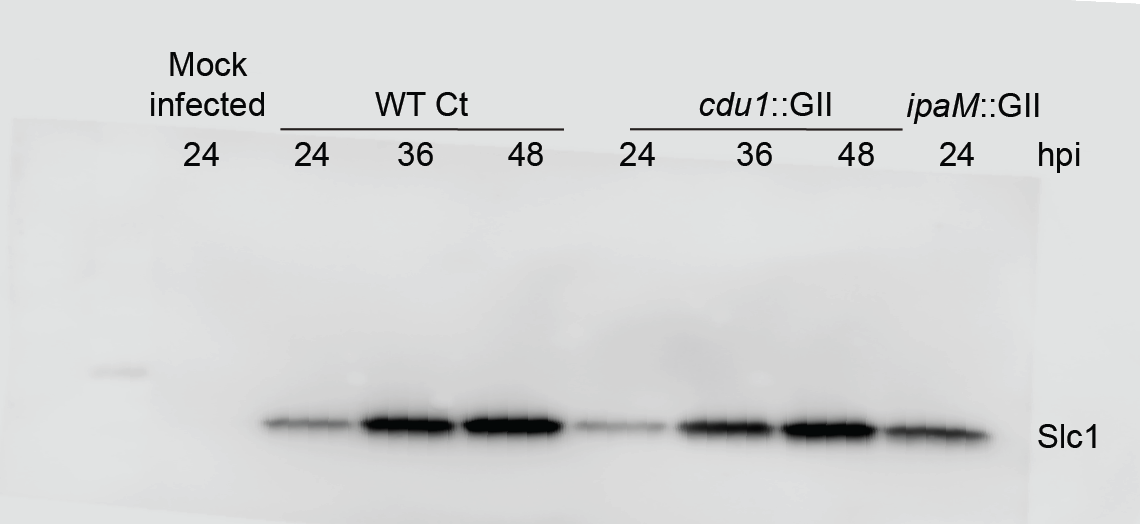

Supplement: Figure 3—source data 5. [file elife-87386-fig3-data5.zip › 20_Figure 3-source data 5/Slc1 WB_Annotated.png]

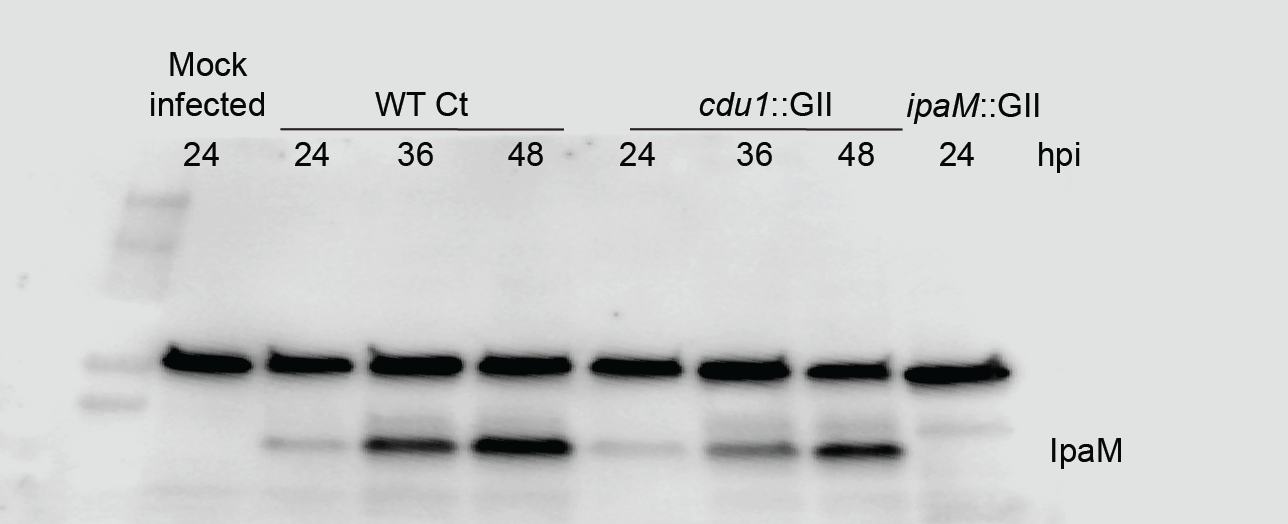

Supplement: Figure 3—source data 5. [file elife-87386-fig3-data5.zip › 20_Figure 3-source data 5/IPAM WB_Annotated.png]

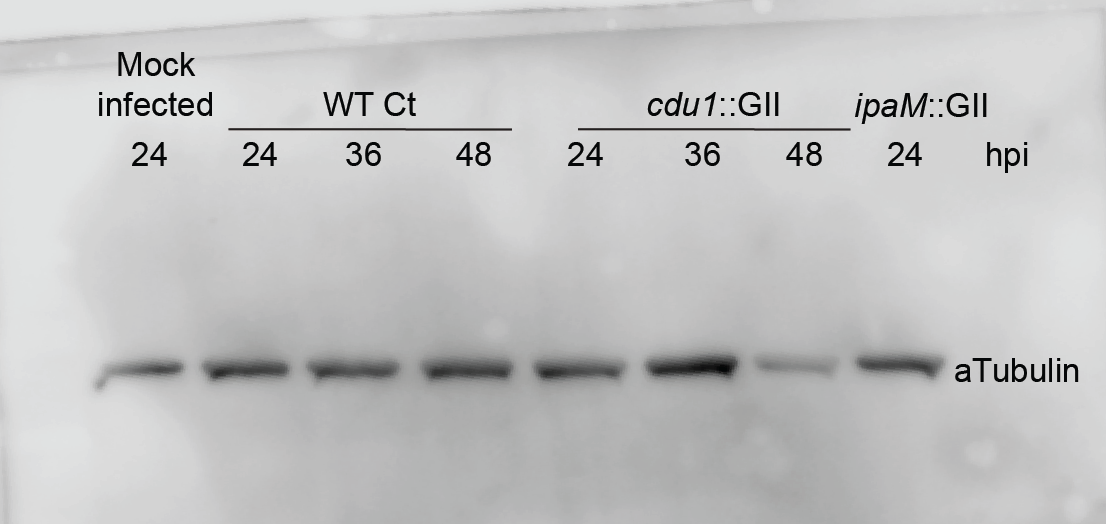

Supplement: Figure 3—source data 5. [file elife-87386-fig3-data5.zip › 20_Figure 3-source data 5/aTubulin WB_Annotated.png]

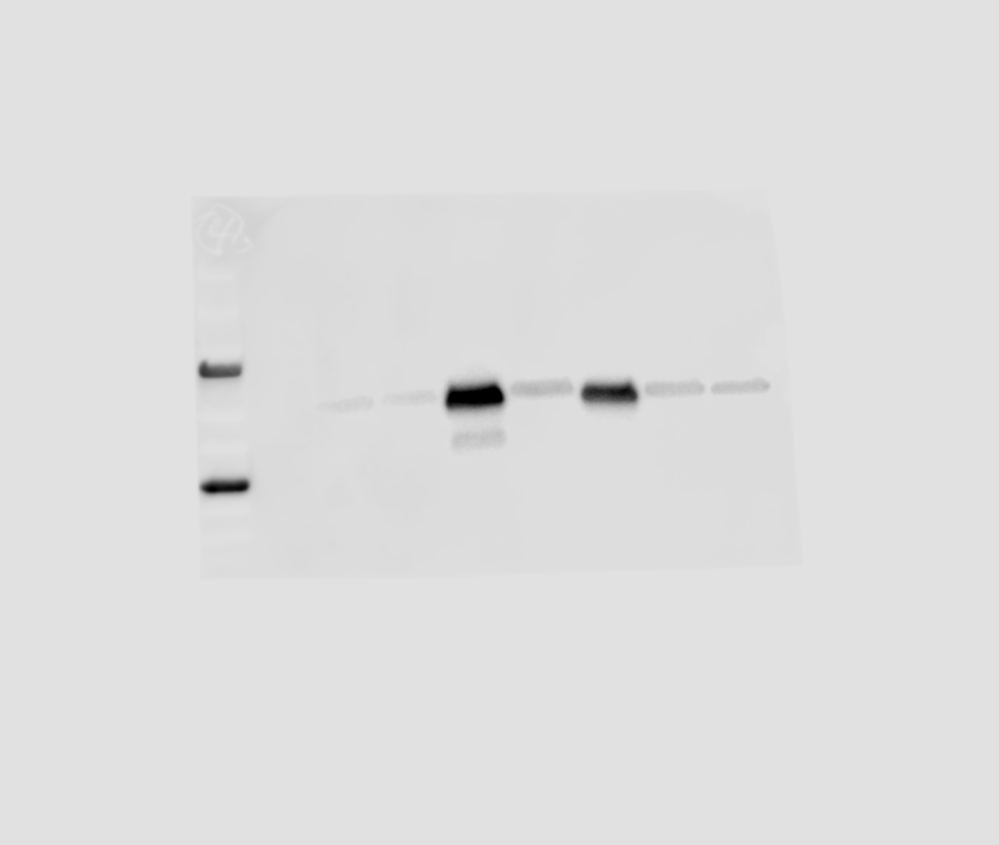

Supplement: Figure 4—source data 1. [file elife-87386-fig4-data1.zip › Figure 4-source data 1/Flag WB.png]

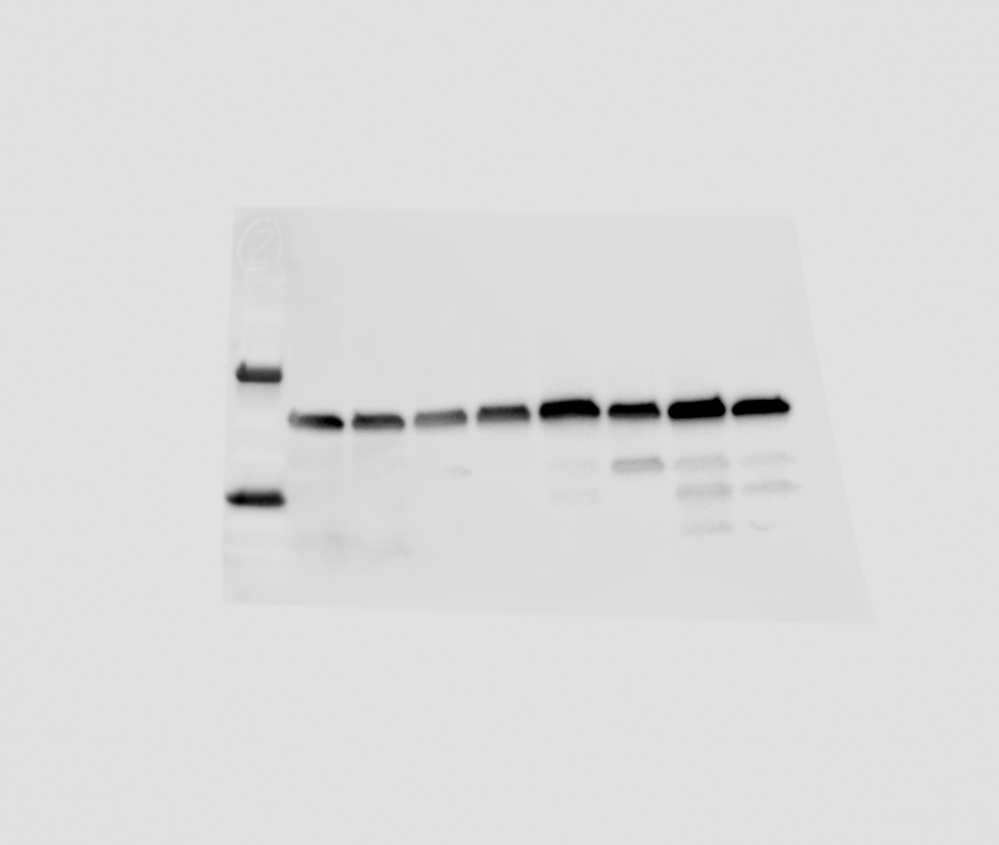

Supplement: Figure 4—source data 1. [file elife-87386-fig4-data1.zip › Figure 4-source data 1/aTubulin WB.png]

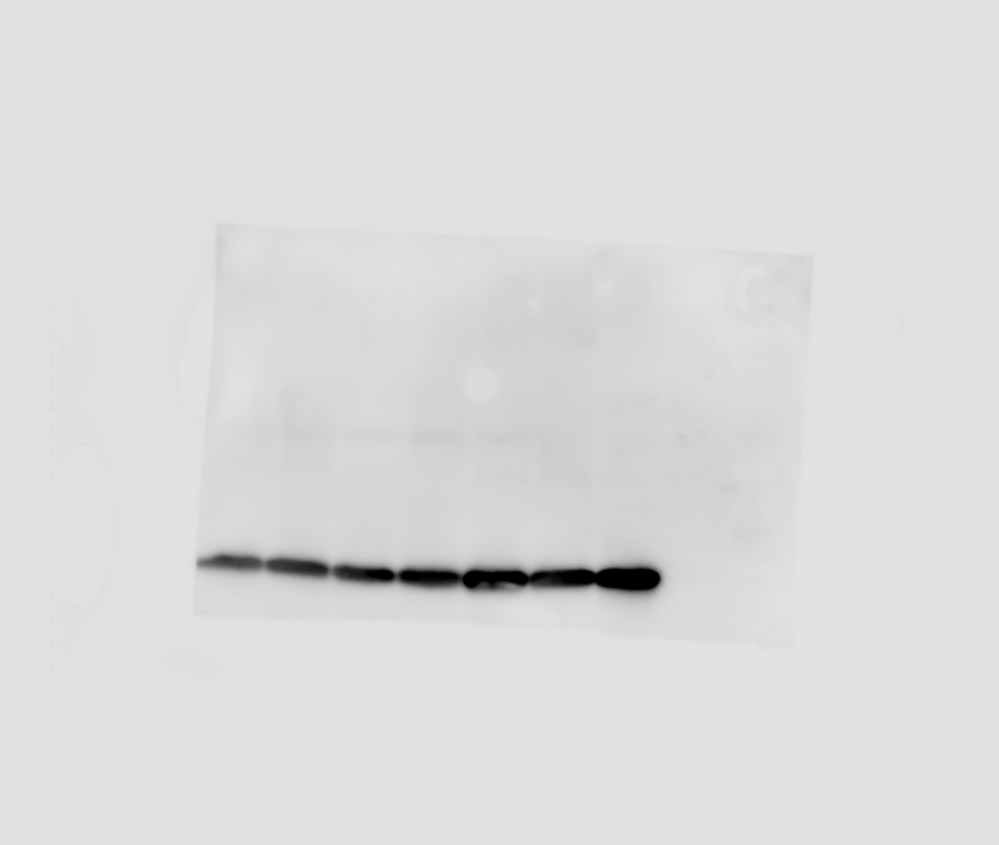

Supplement: Figure 4—source data 1. [file elife-87386-fig4-data1.zip › Figure 4-source data 1/Slc1 WB.png]

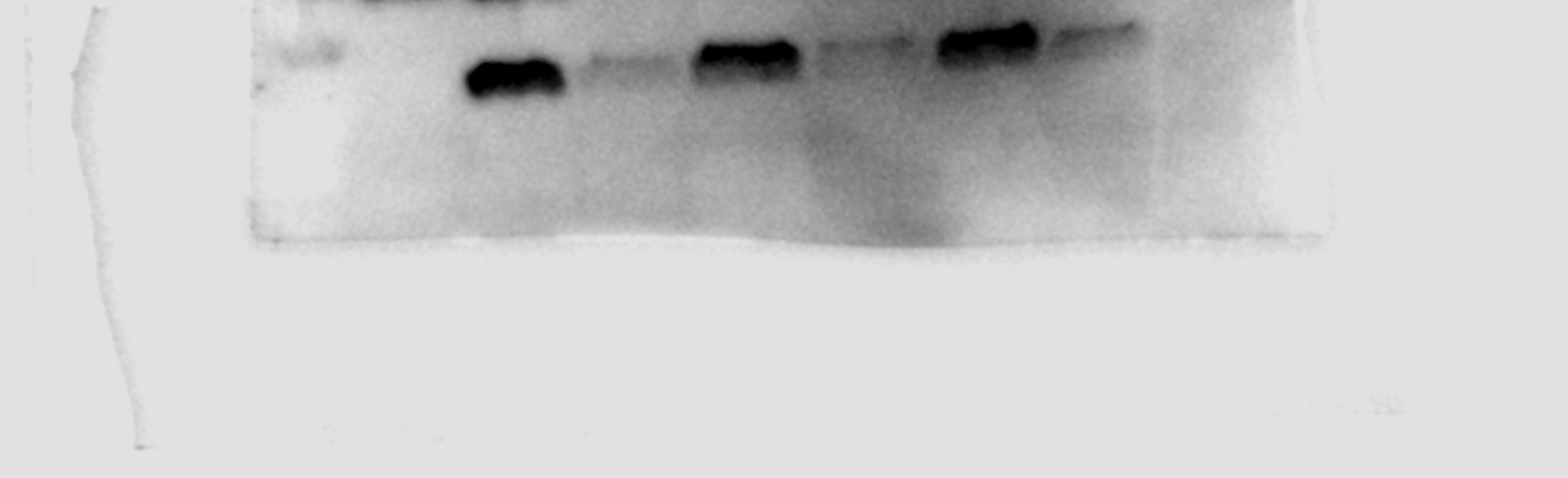

Supplement: Figure 4—source data 1. [file elife-87386-fig4-data1.zip › Figure 4-source data 1/InaC WB.png]

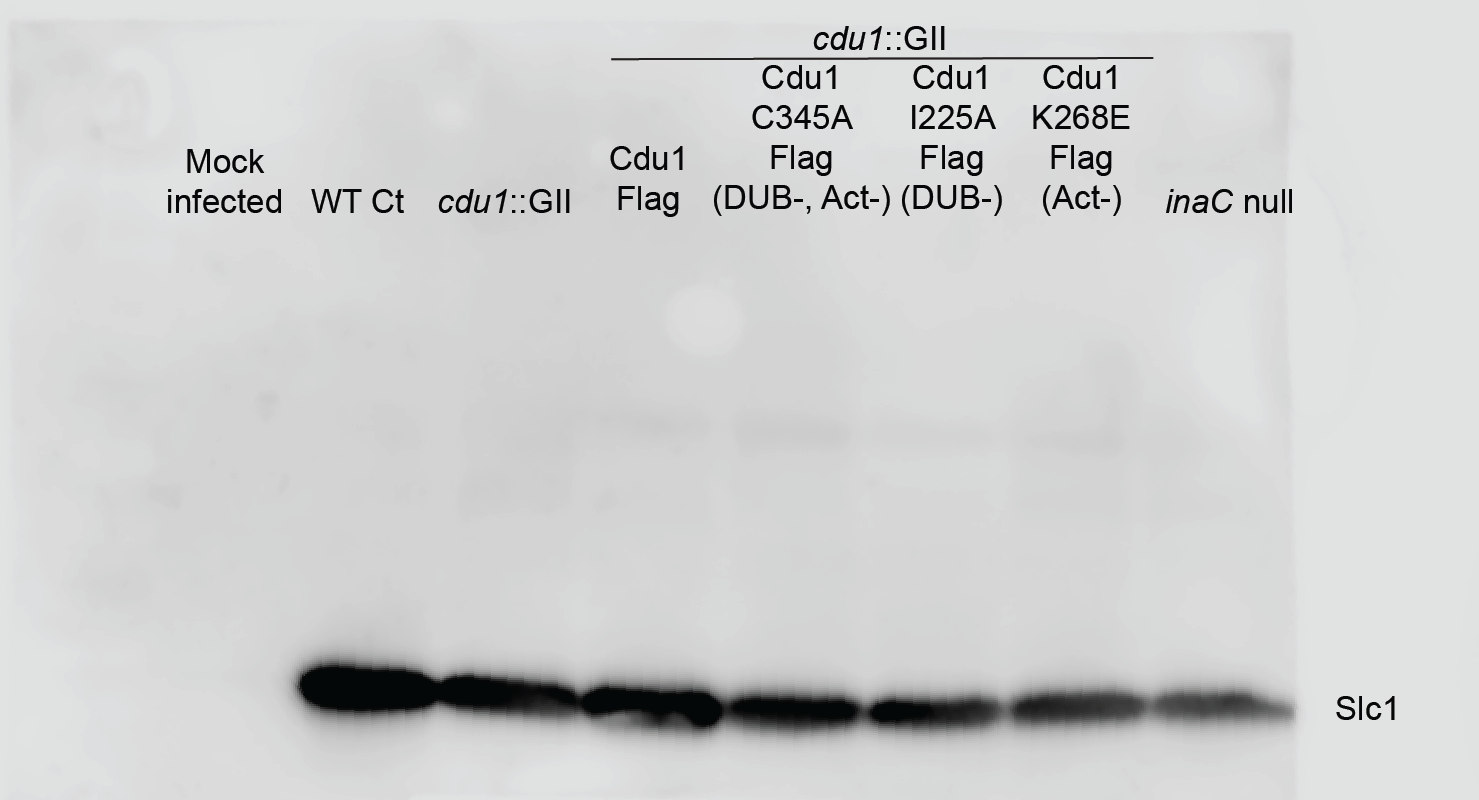

Supplement: Figure 4—source data 2. [file elife-87386-fig4-data2.zip › Figure 4-source data 2/Slc1 WB_Annotated.png]

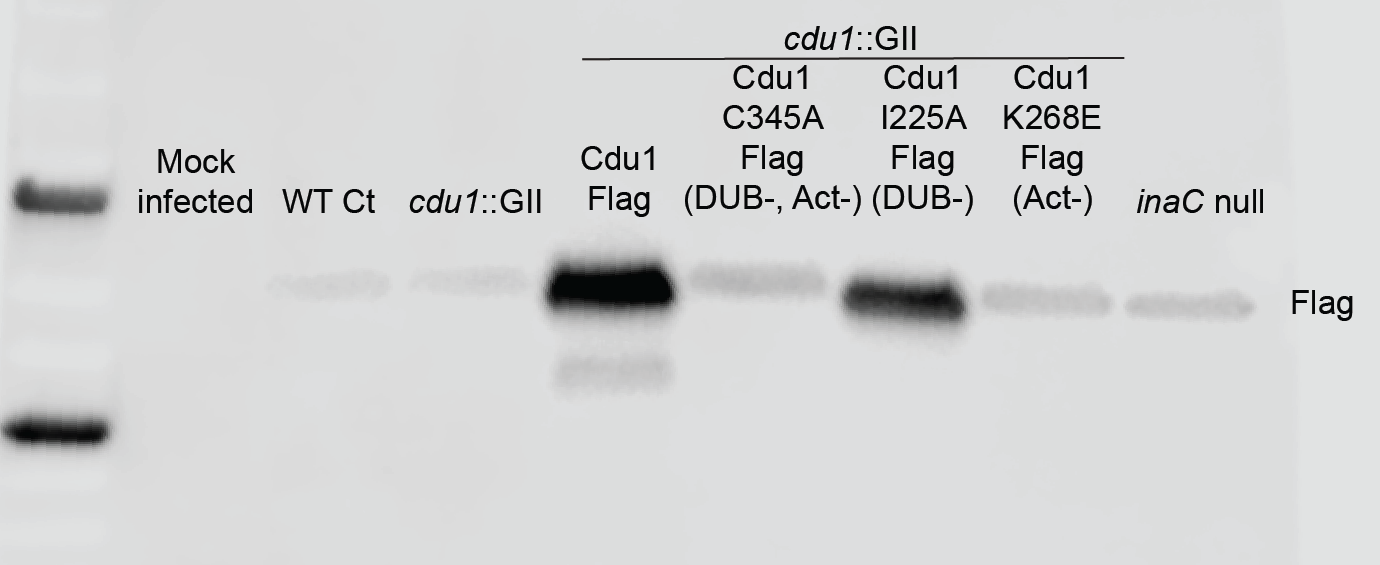

Supplement: Figure 4—source data 2. [file elife-87386-fig4-data2.zip › Figure 4-source data 2/Flag WB_Annotated.png]

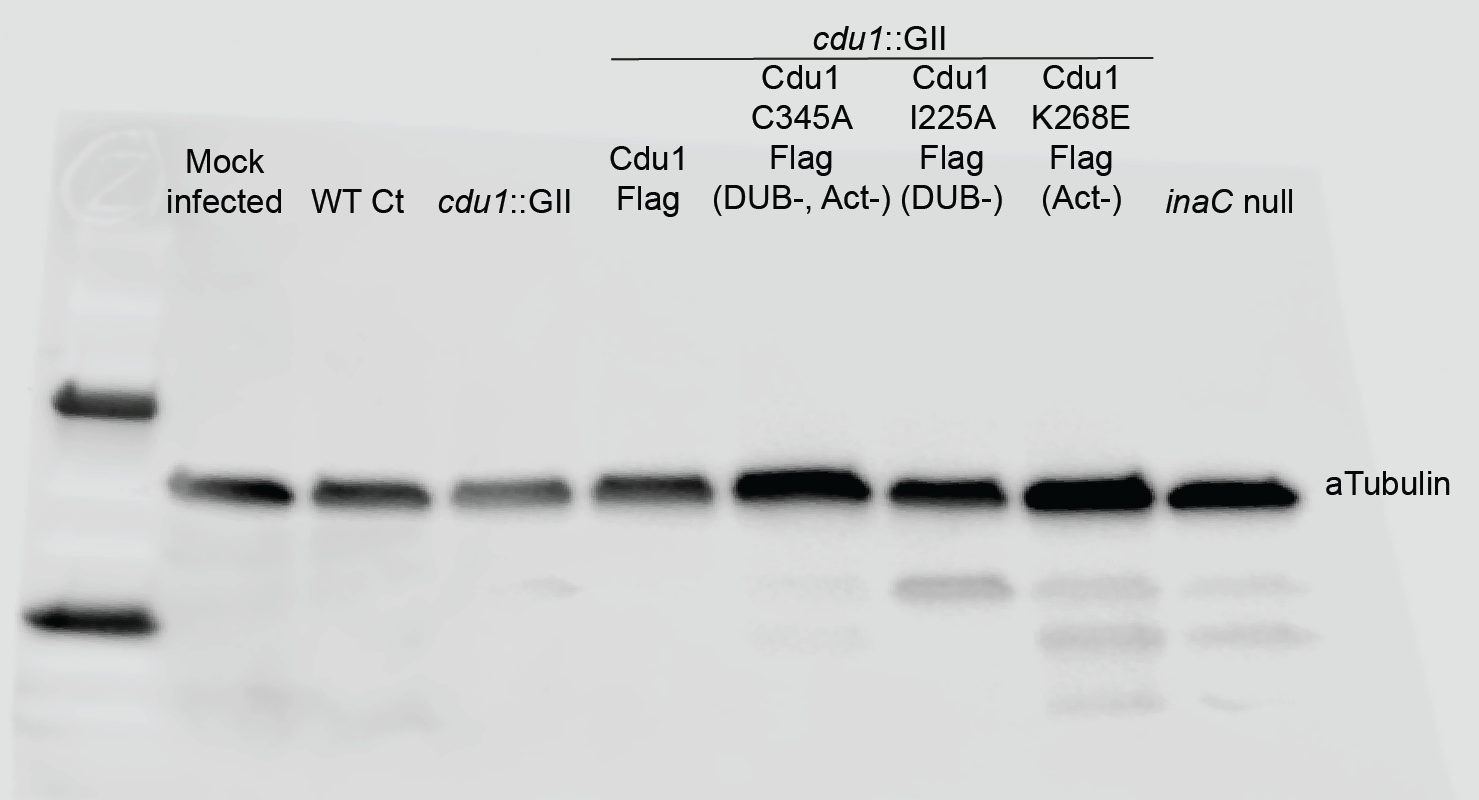

Supplement: Figure 4—source data 2. [file elife-87386-fig4-data2.zip › Figure 4-source data 2/aTubulin WB_Annotated.png]

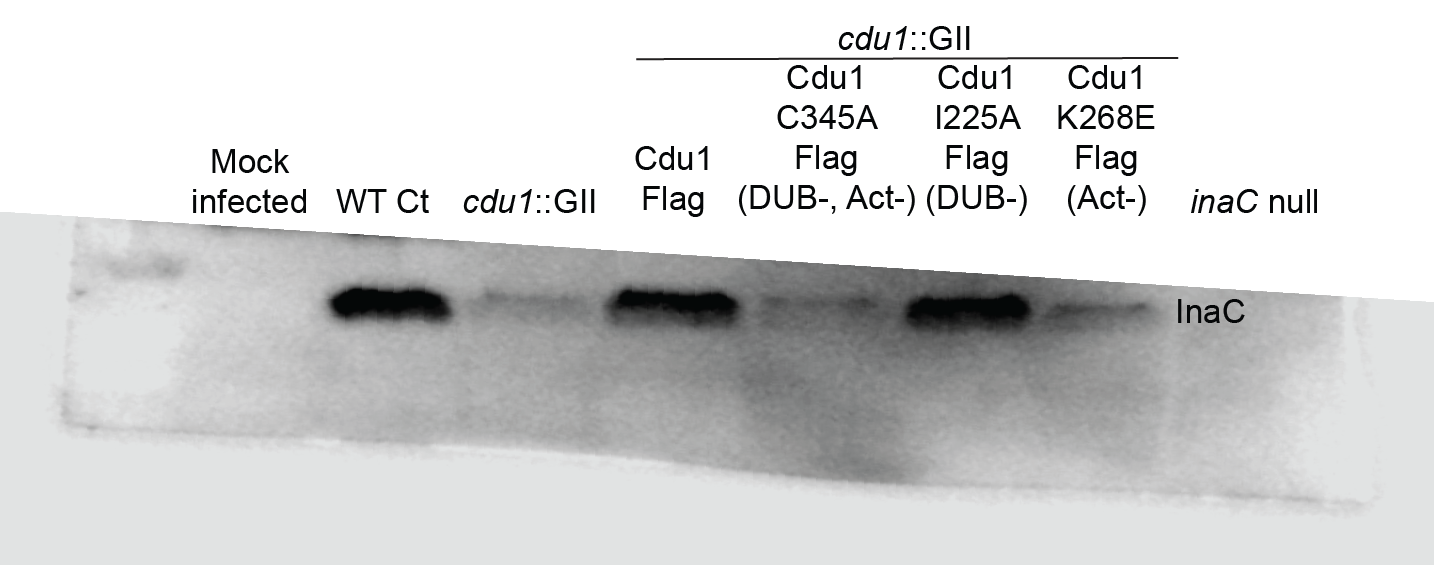

Supplement: Figure 4—source data 2. [file elife-87386-fig4-data2.zip › Figure 4-source data 2/InaC WB_Annotated.png]

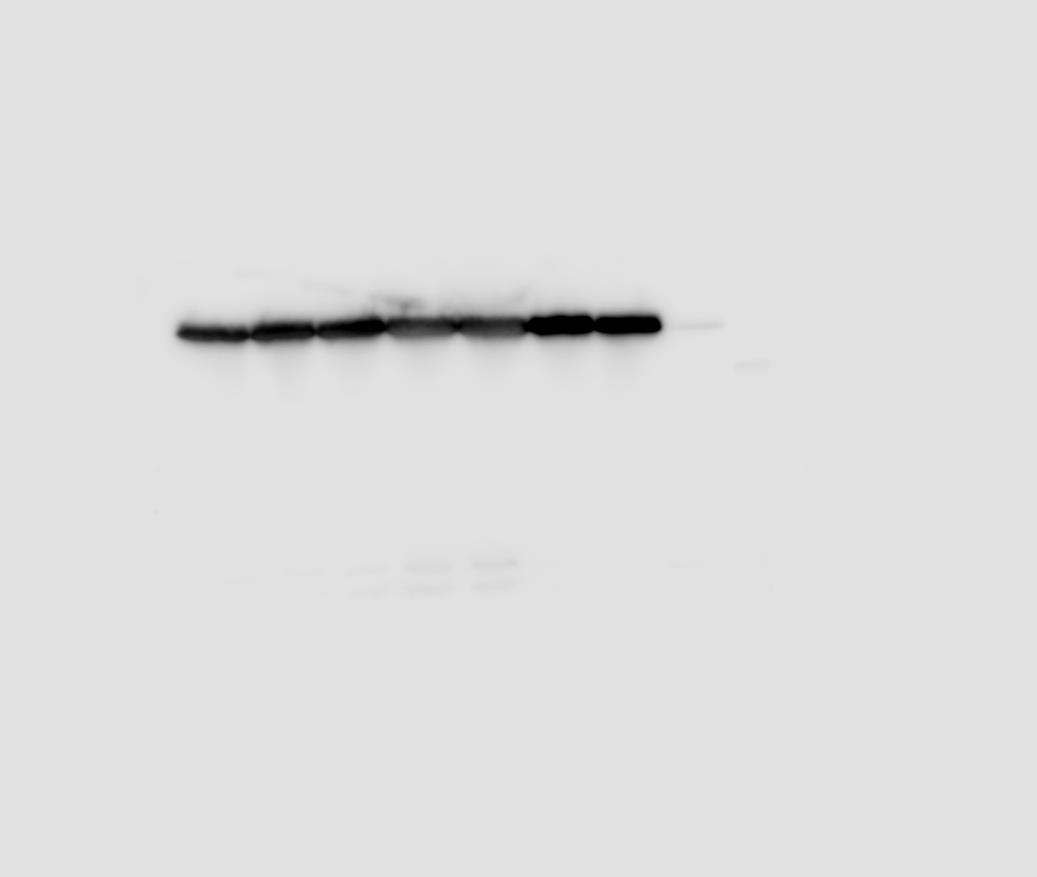

Supplement: Figure 4—source data 3. [file elife-87386-fig4-data3.zip › Figure 4-source data 3/Slc1 WB.png]

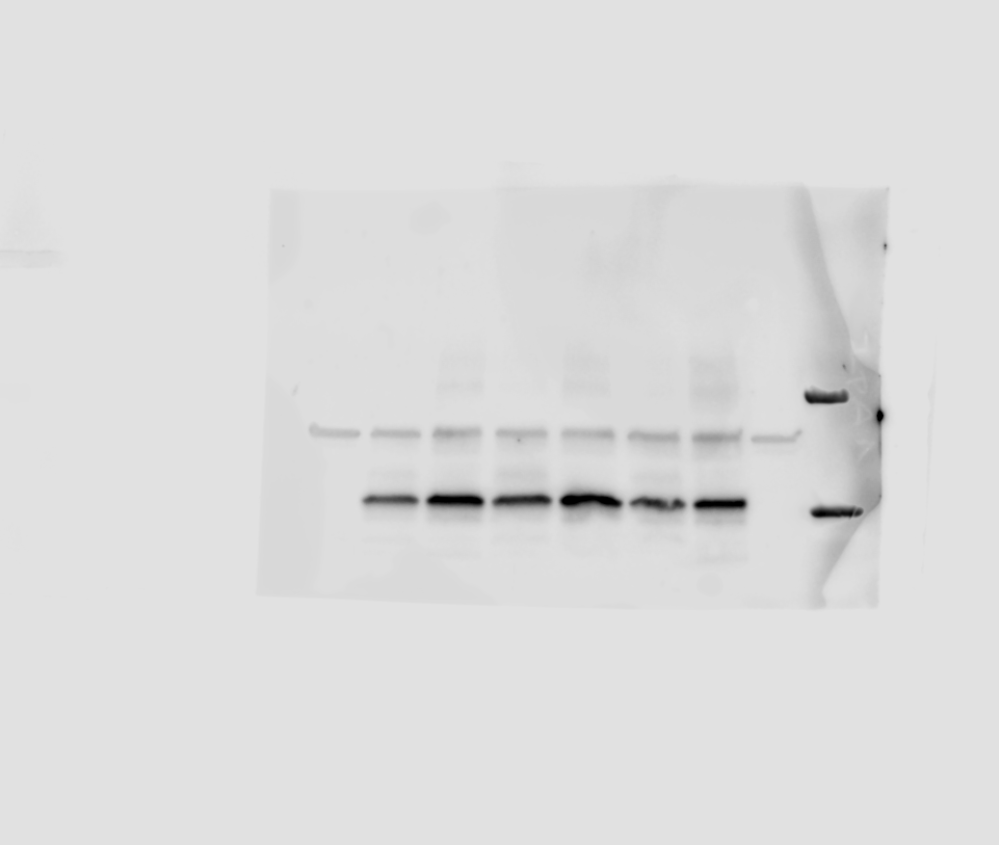

Supplement: Figure 4—source data 3. [file elife-87386-fig4-data3.zip › Figure 4-source data 3/IPAM WB.png]

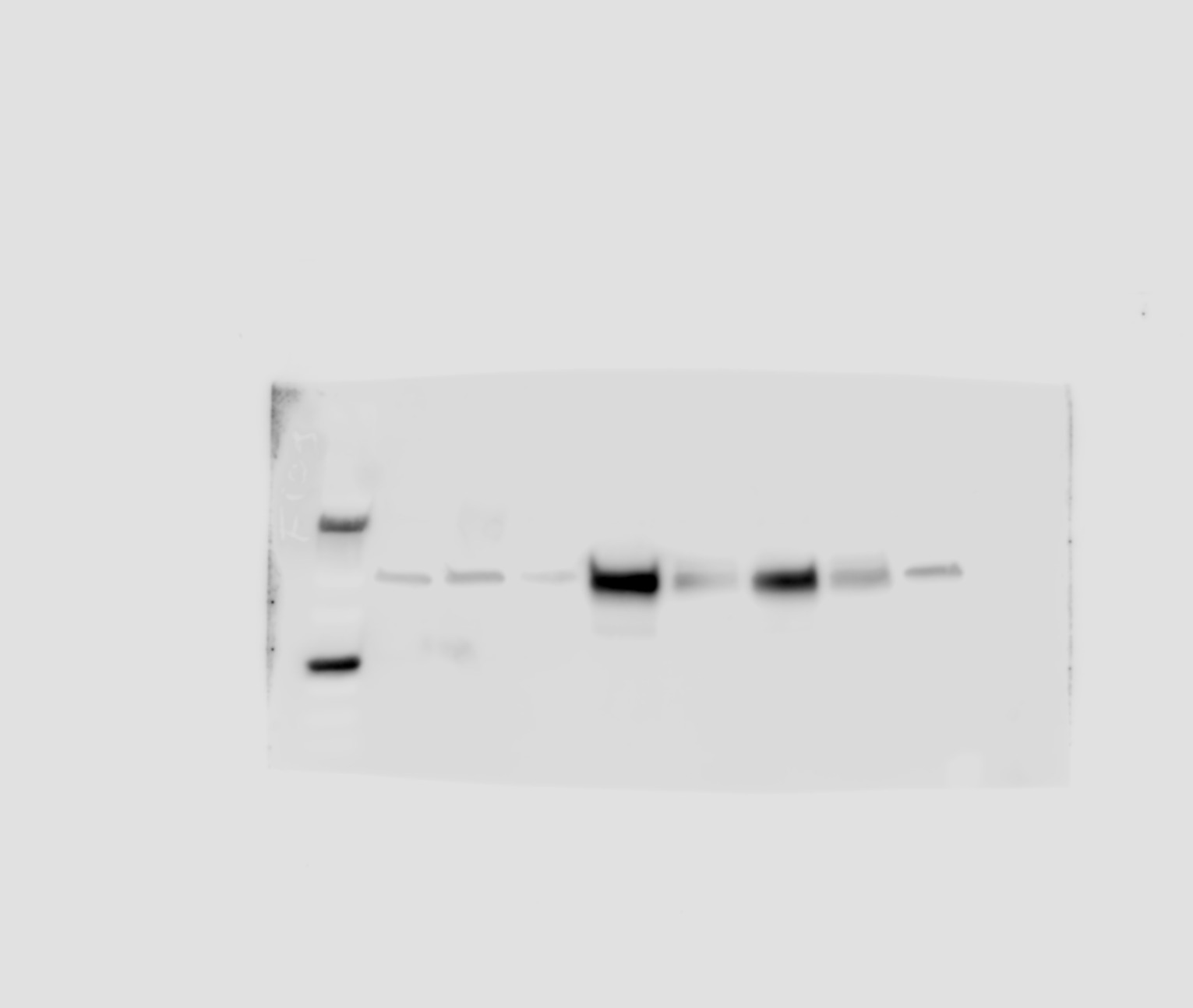

Supplement: Figure 4—source data 3. [file elife-87386-fig4-data3.zip › Figure 4-source data 3/Flag WB.tif]

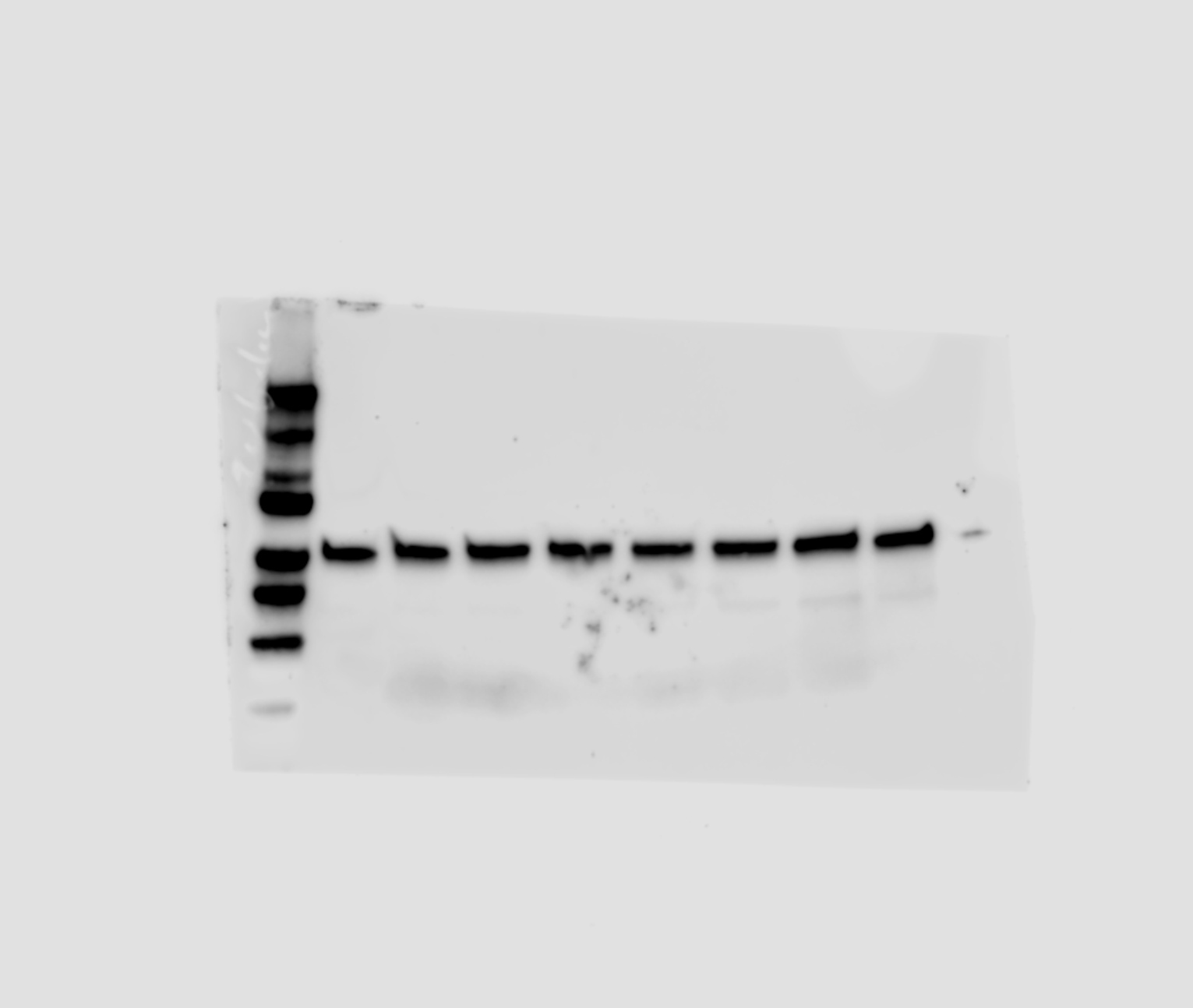

Supplement: Figure 4—source data 3. [file elife-87386-fig4-data3.zip › Figure 4-source data 3/aTubulin WB.tif]

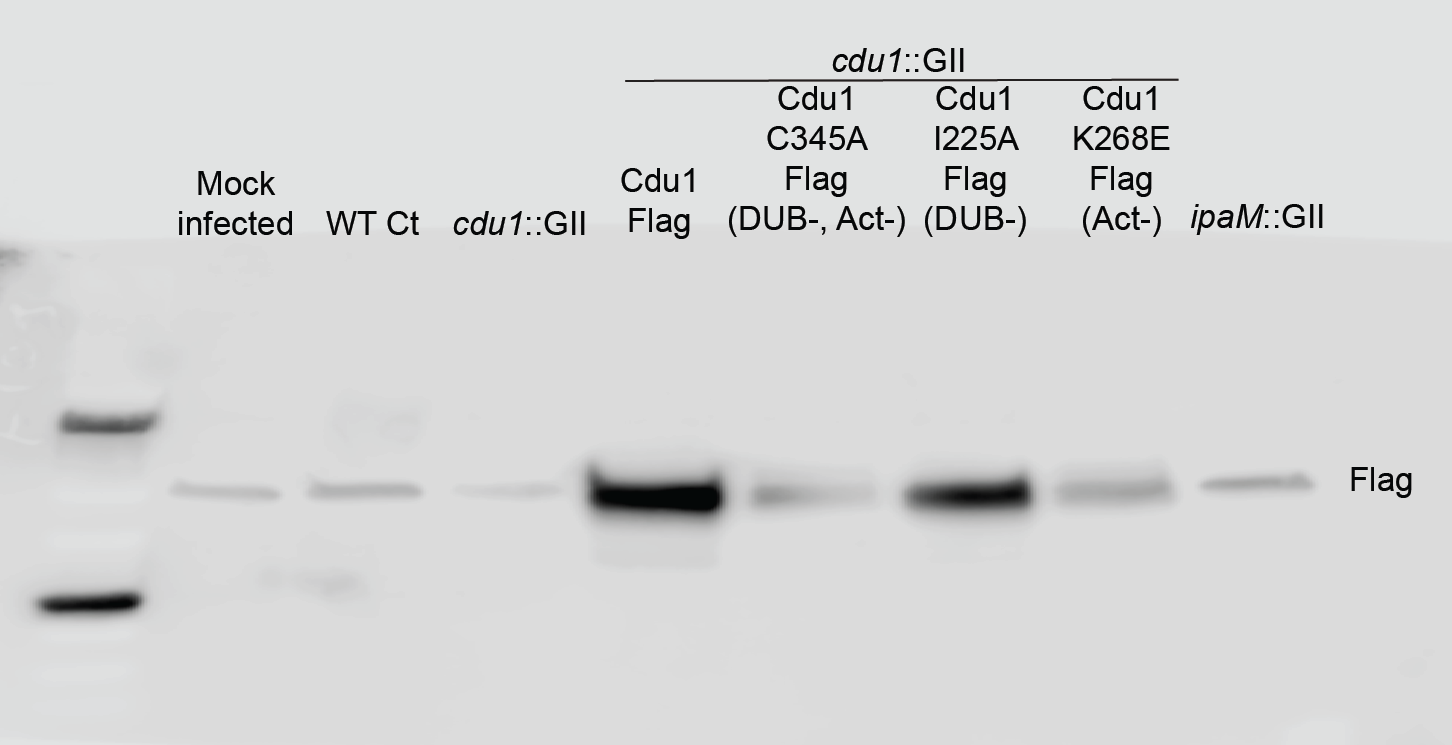

Supplement: Figure 4—source data 4. [file elife-87386-fig4-data4.zip › Figure 4-source data 4/Flag WB.png]

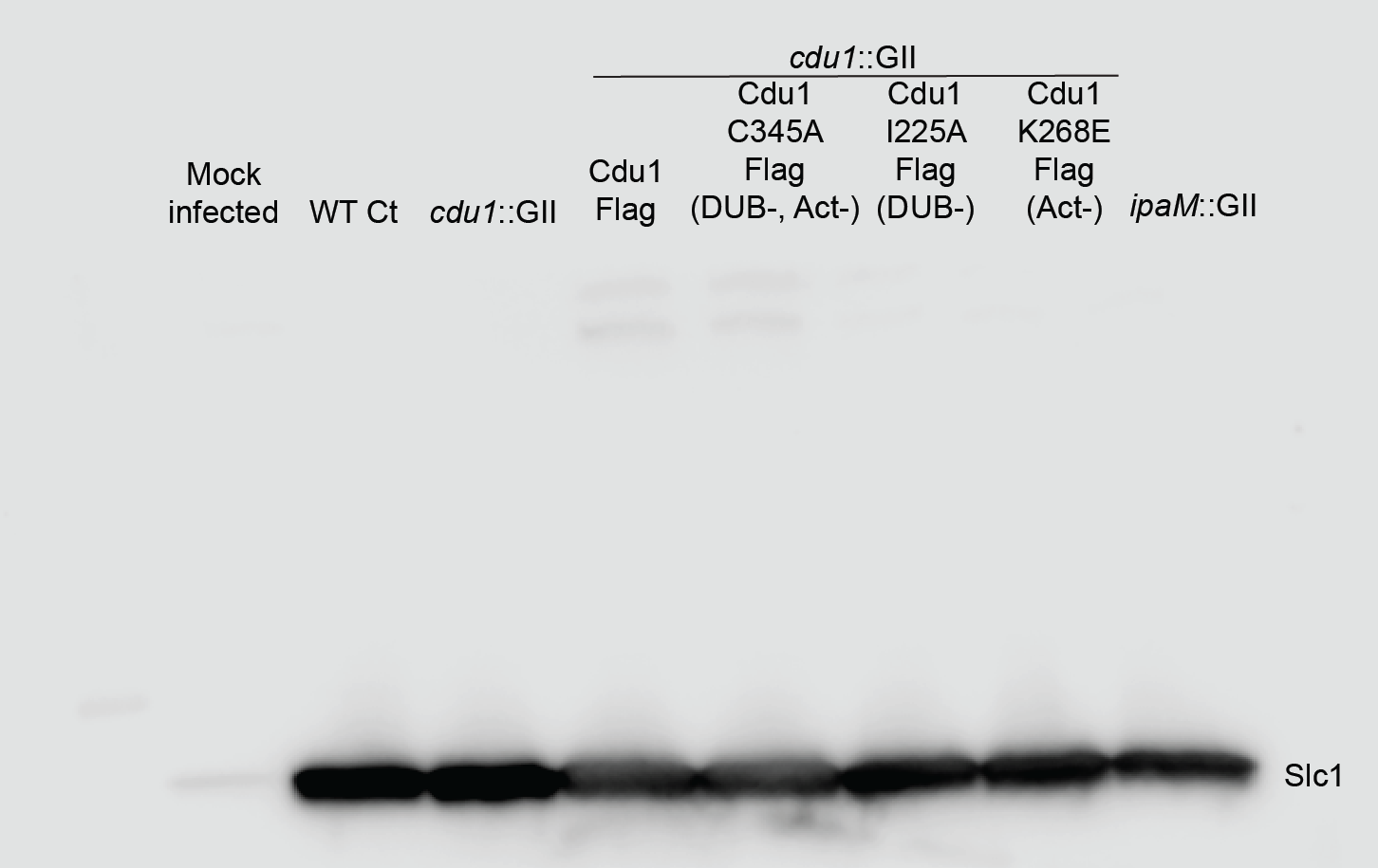

Supplement: Figure 4—source data 4. [file elife-87386-fig4-data4.zip › Figure 4-source data 4/Slc1 WB_Annotated.png]

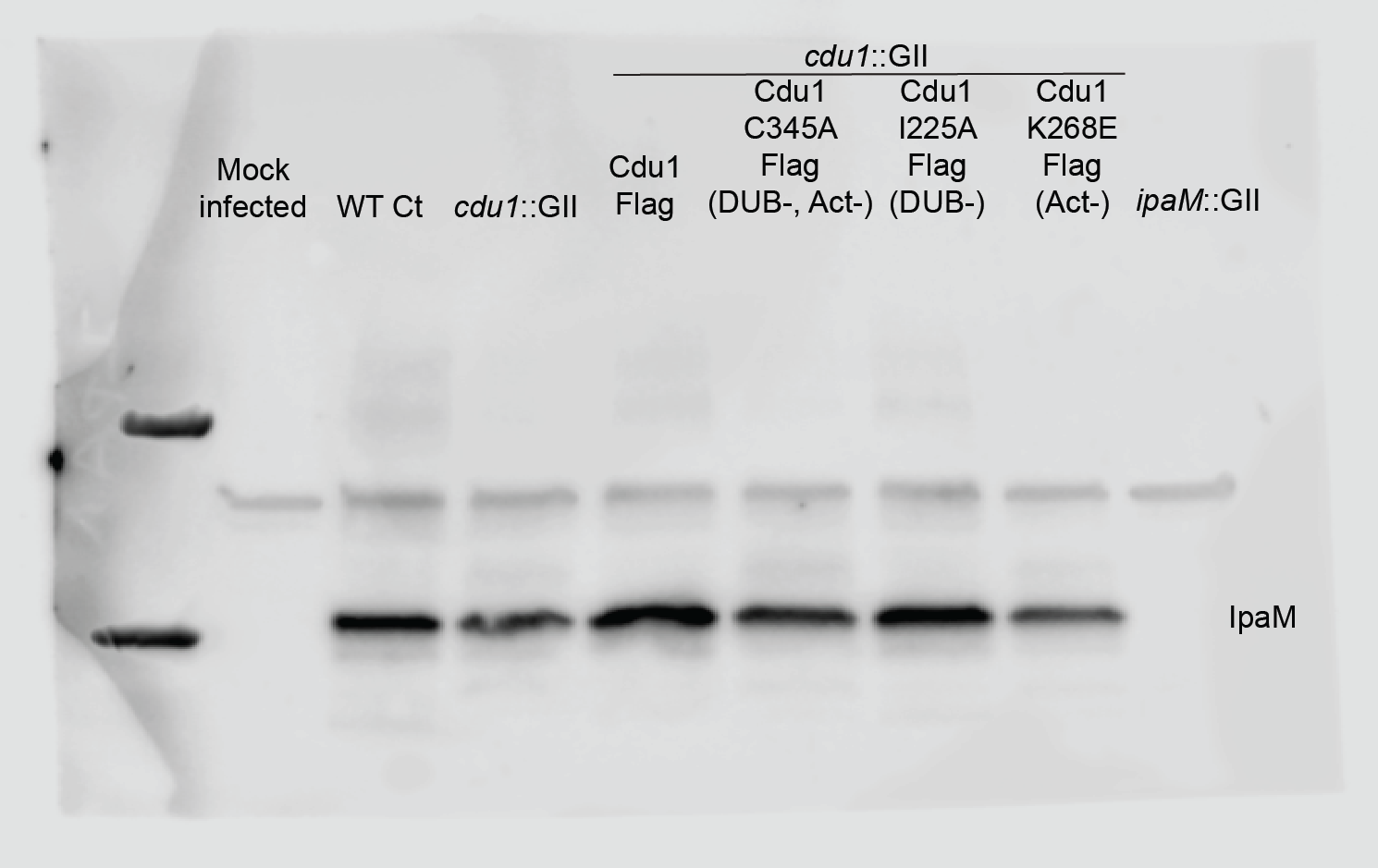

Supplement: Figure 4—source data 4. [file elife-87386-fig4-data4.zip › Figure 4-source data 4/IPAM WB_Annotated.png]

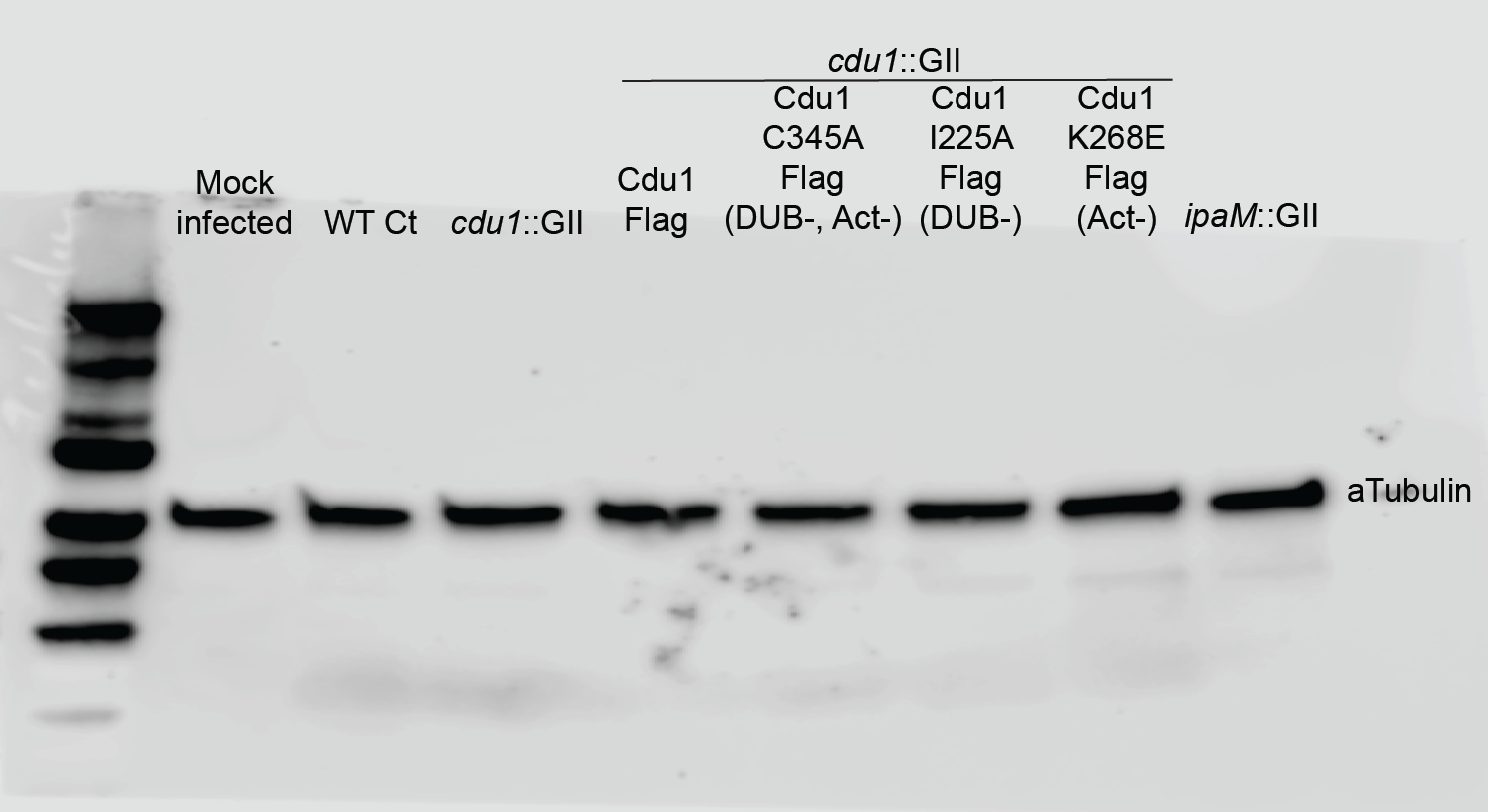

Supplement: Figure 4—source data 4. [file elife-87386-fig4-data4.zip › Figure 4-source data 4/aTubulin WB_Annotated.png]

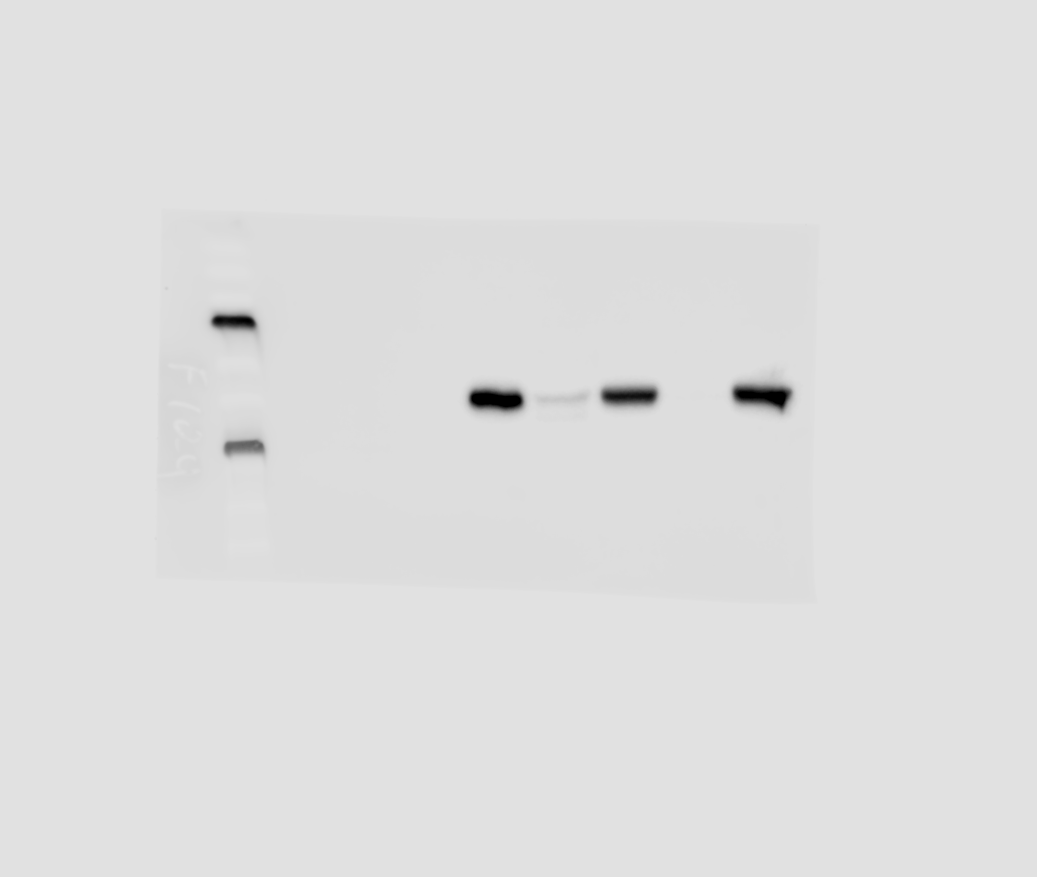

Supplement: Figure 4—source data 5. [file elife-87386-fig4-data5.zip › Figure 4-source data 5/Flag WB.png]

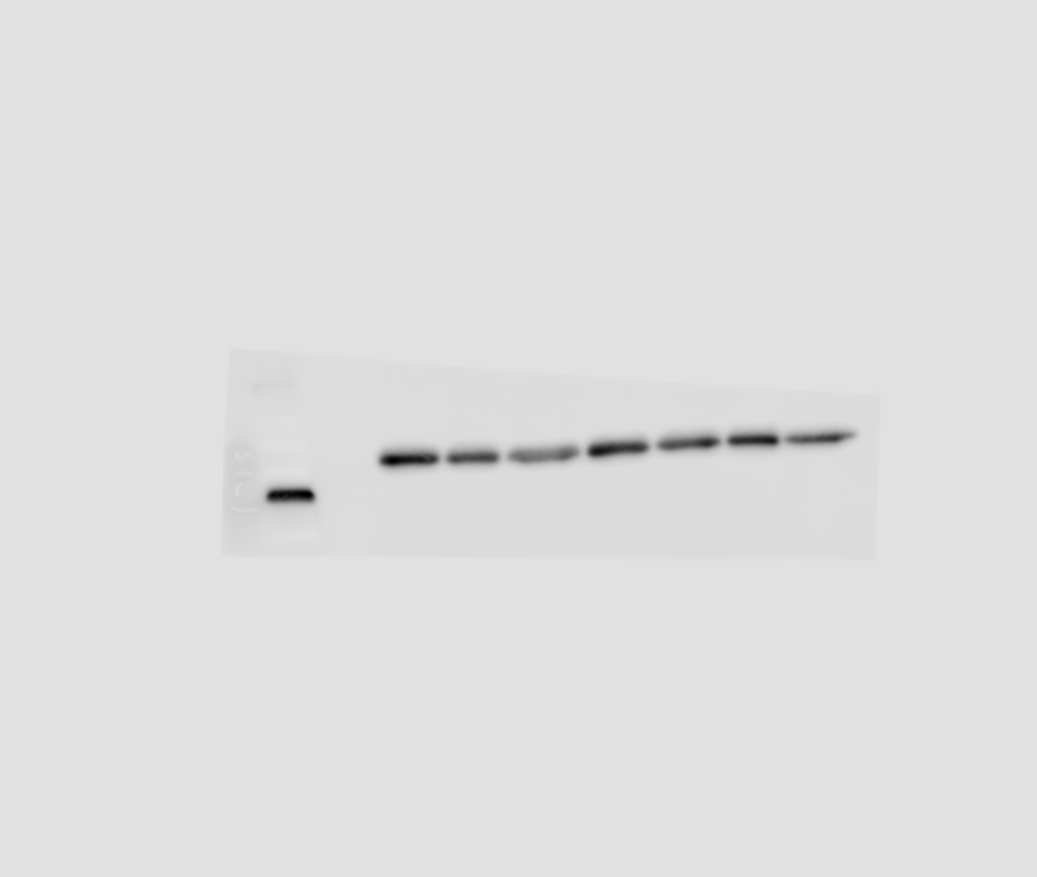

Supplement: Figure 4—source data 5. [file elife-87386-fig4-data5.zip › Figure 4-source data 5/Slc1 WB.png]

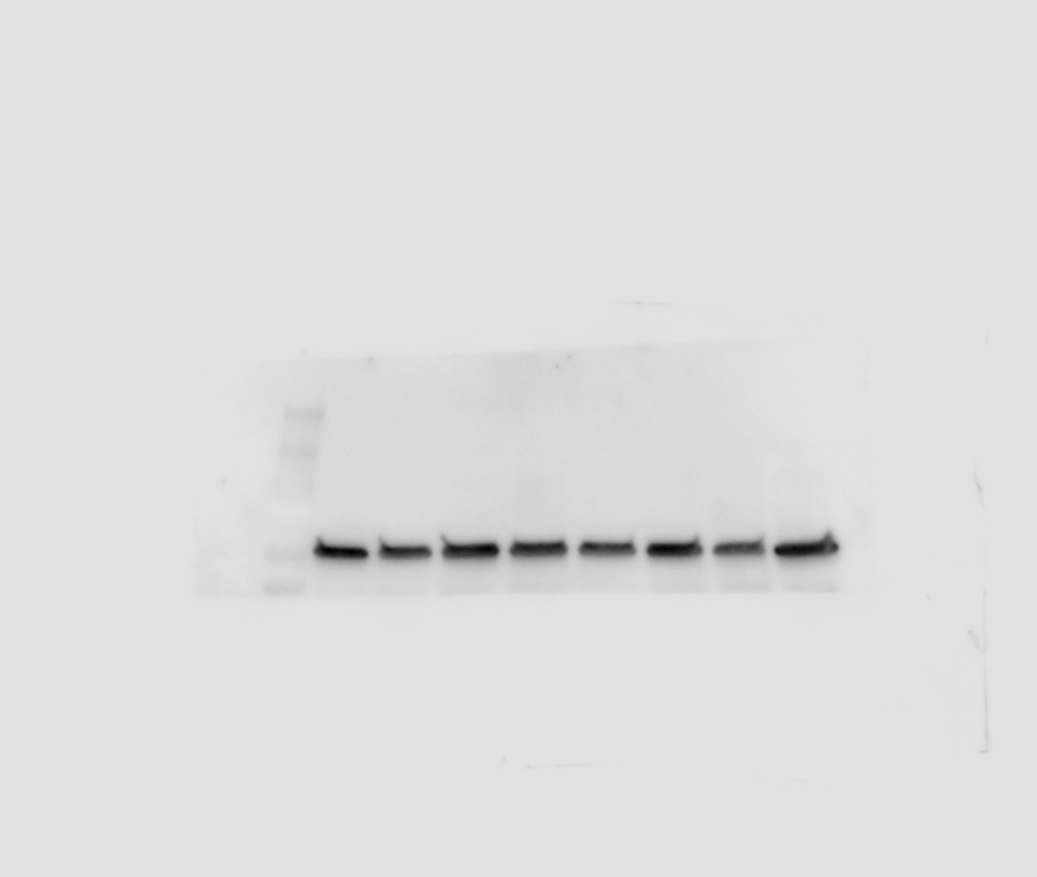

Supplement: Figure 4—source data 5. [file elife-87386-fig4-data5.zip › Figure 4-source data 5/Tubulin WB.png]

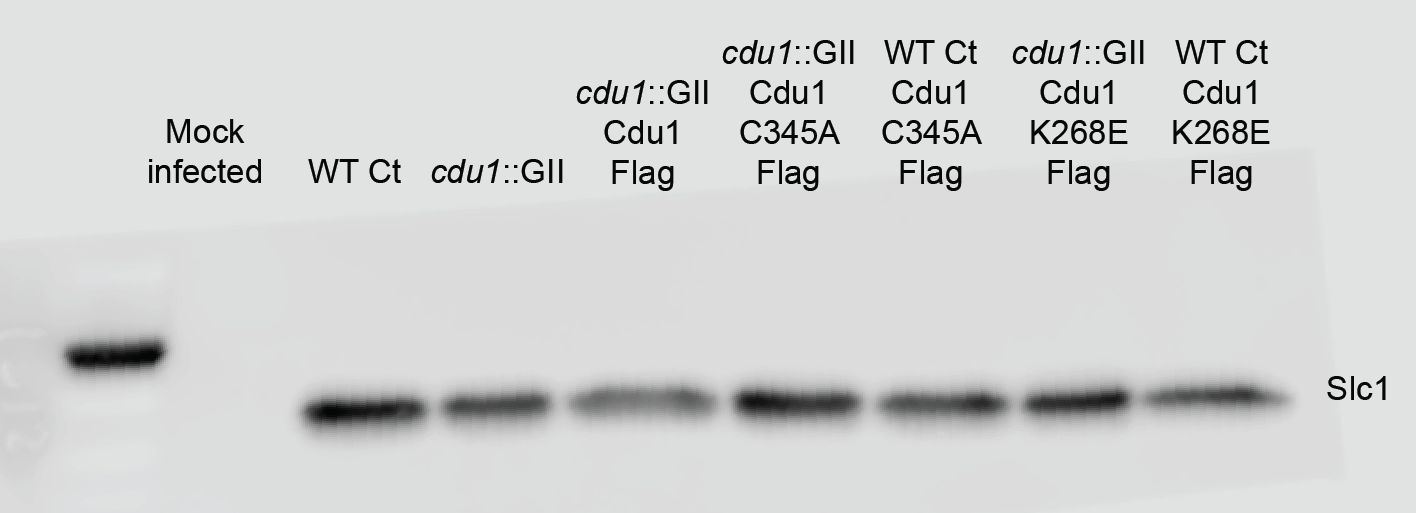

Supplement: Figure 4—source data 6. [file elife-87386-fig4-data6.zip › Figure 4-source data 6/Slc1 WB_Annotated.png]

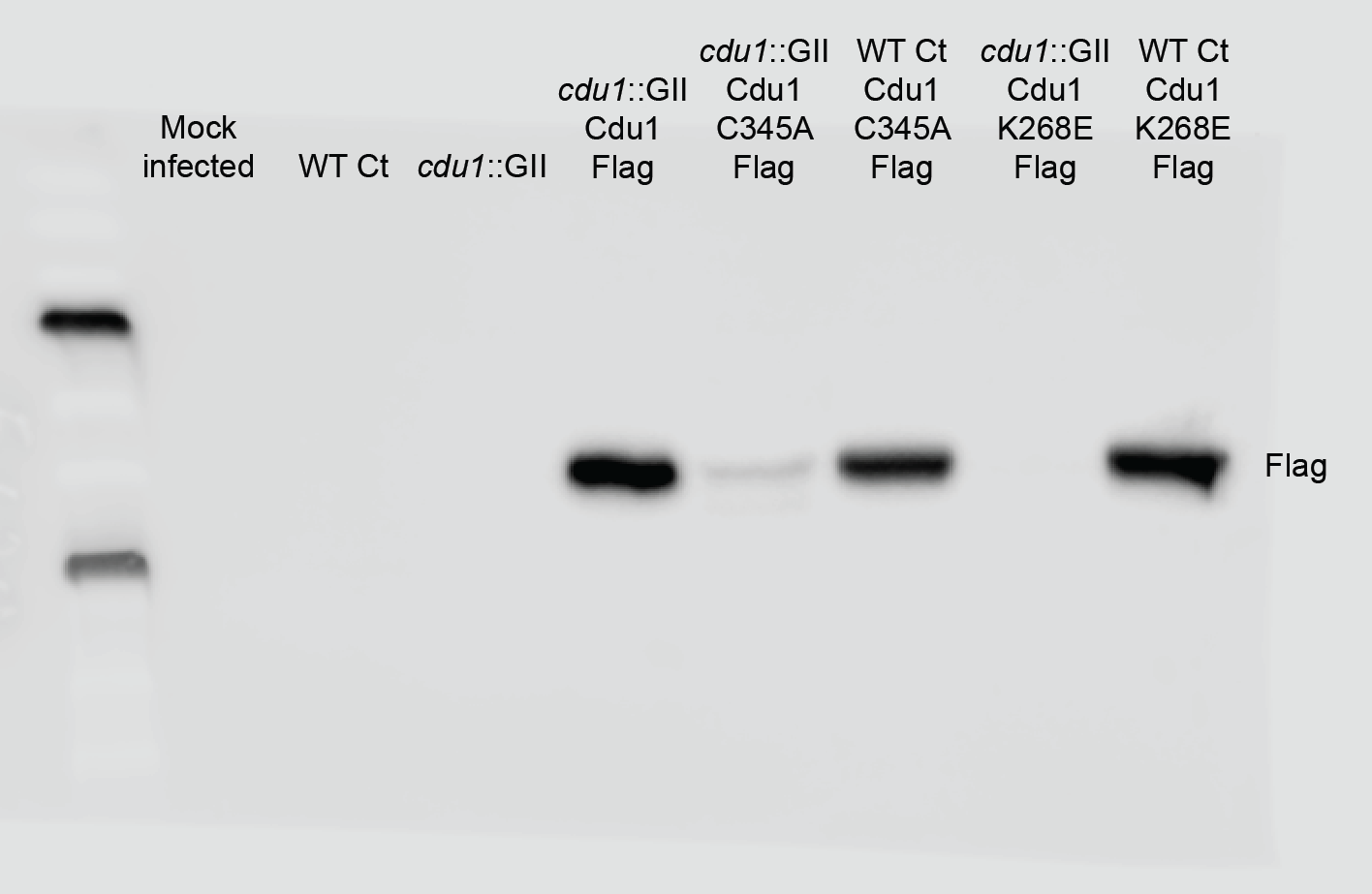

Supplement: Figure 4—source data 6. [file elife-87386-fig4-data6.zip › Figure 4-source data 6/Flag WB_Annotated.png]

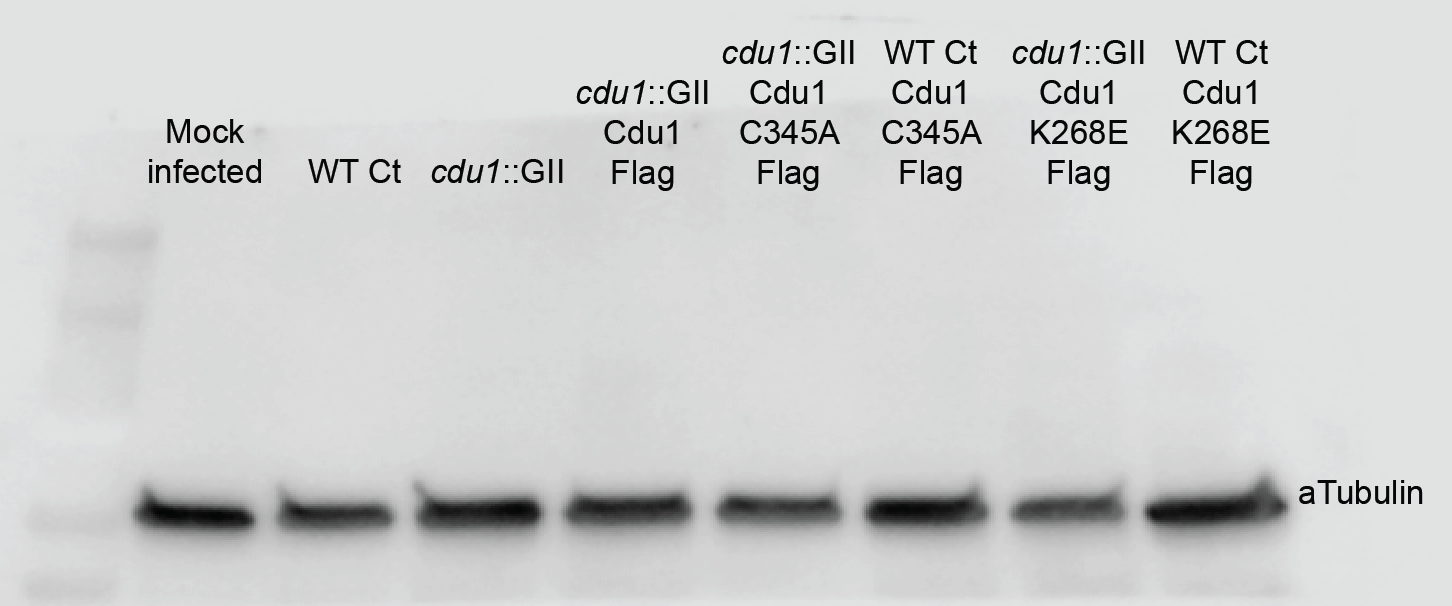

Supplement: Figure 4—source data 6. [file elife-87386-fig4-data6.zip › Figure 4-source data 6/Tubulin WB.png]

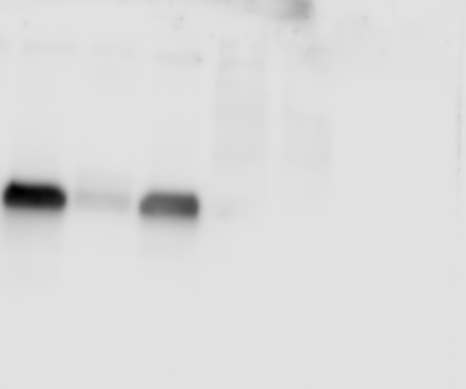

Supplement: Figure 4—source data 7. [file elife-87386-fig4-data7.zip › Figure 4-source data 7/Input_Flag WB.png]

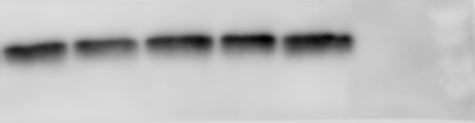

Supplement: Figure 4—source data 7. [file elife-87386-fig4-data7.zip › Figure 4-source data 7/Input_Slc1 WB.png]

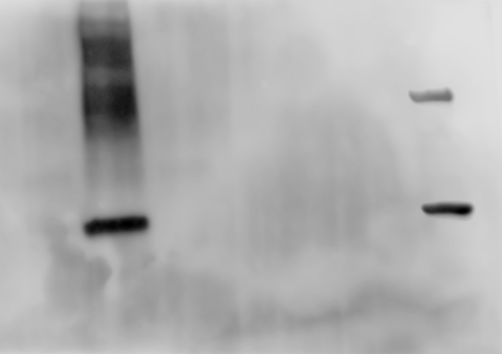

Supplement: Figure 4—source data 7. [file elife-87386-fig4-data7.zip › Figure 4-source data 7/Flag IP_k48 pUb WB.png]

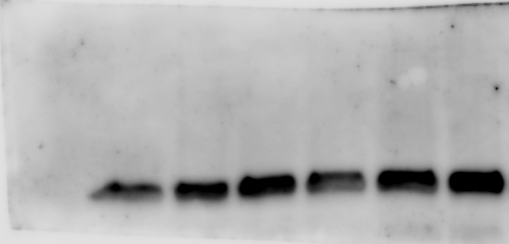

Supplement: Figure 4—source data 7. [file elife-87386-fig4-data7.zip › Figure 4-source data 7/Input_a-Tubulin WB.png]

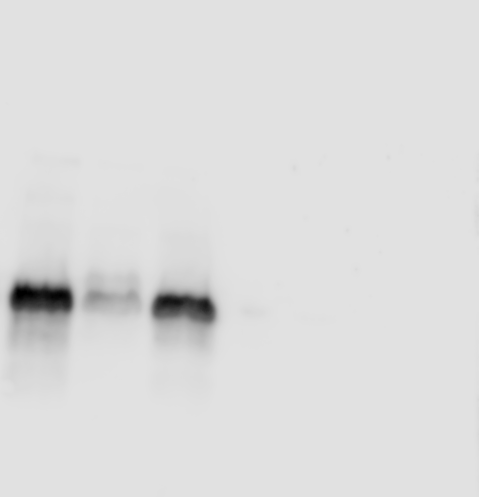

Supplement: Figure 4—source data 7. [file elife-87386-fig4-data7.zip › Figure 4-source data 7/Flag IP_Flag WB.png]

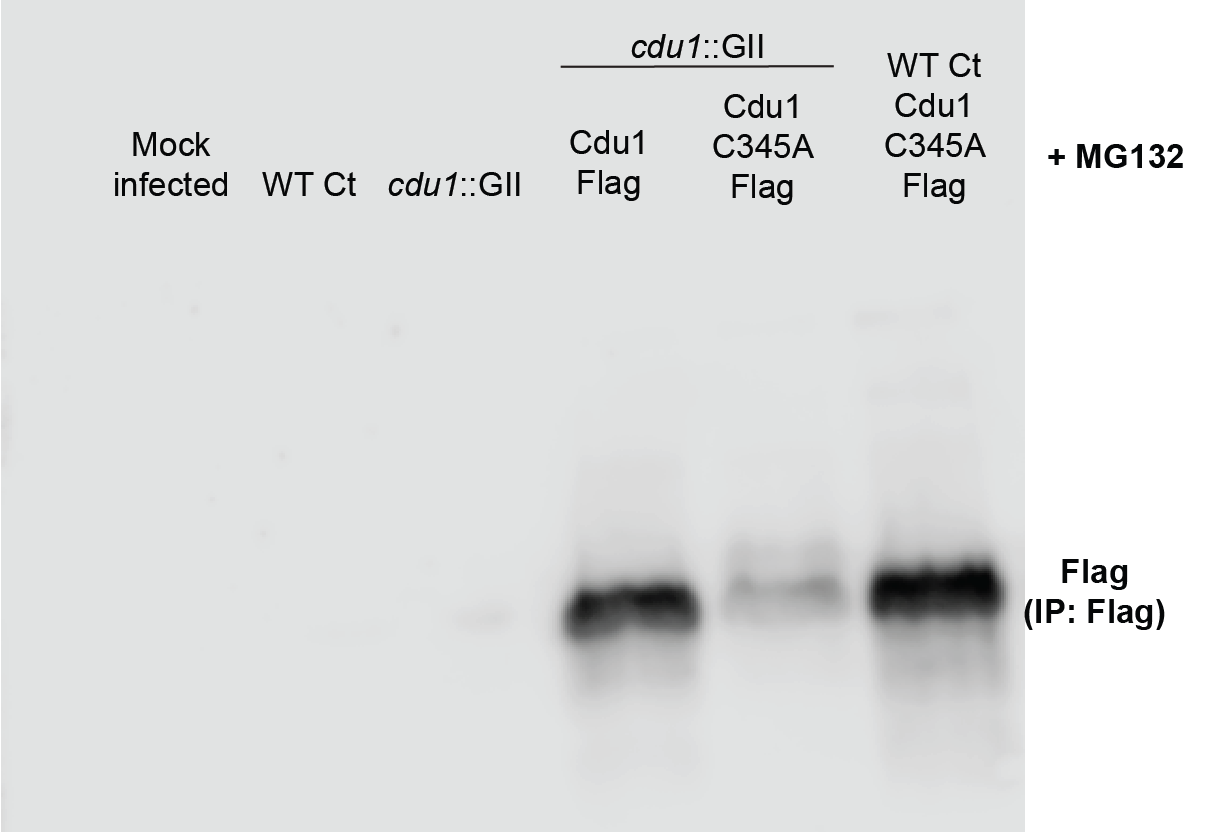

Supplement: Figure 4—source data 8. [file elife-87386-fig4-data8.zip › Figure 4-source data 8/Flag IP_Flag WB_Annotated.png]

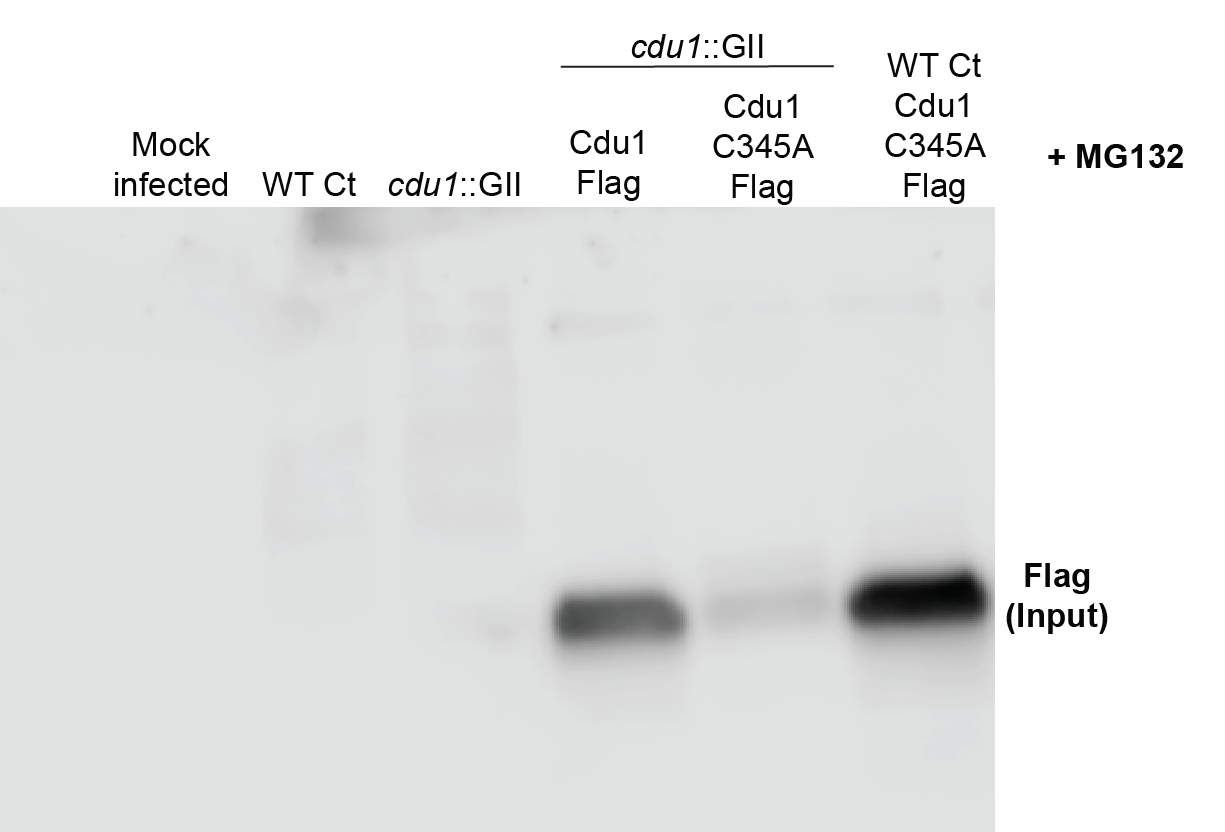

Supplement: Figure 4—source data 8. [file elife-87386-fig4-data8.zip › Figure 4-source data 8/Input_Flag WB_Annotated.png]

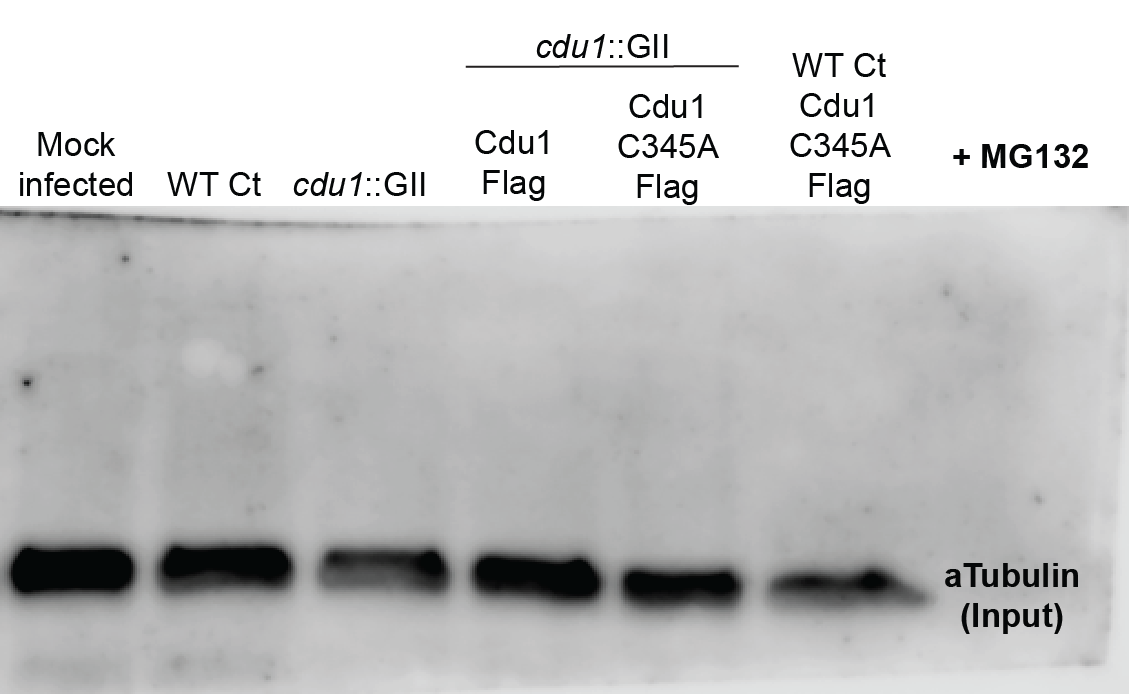

Supplement: Figure 4—source data 8. [file elife-87386-fig4-data8.zip › Figure 4-source data 8/Input_a-Tubulin WB_Annotated.png]

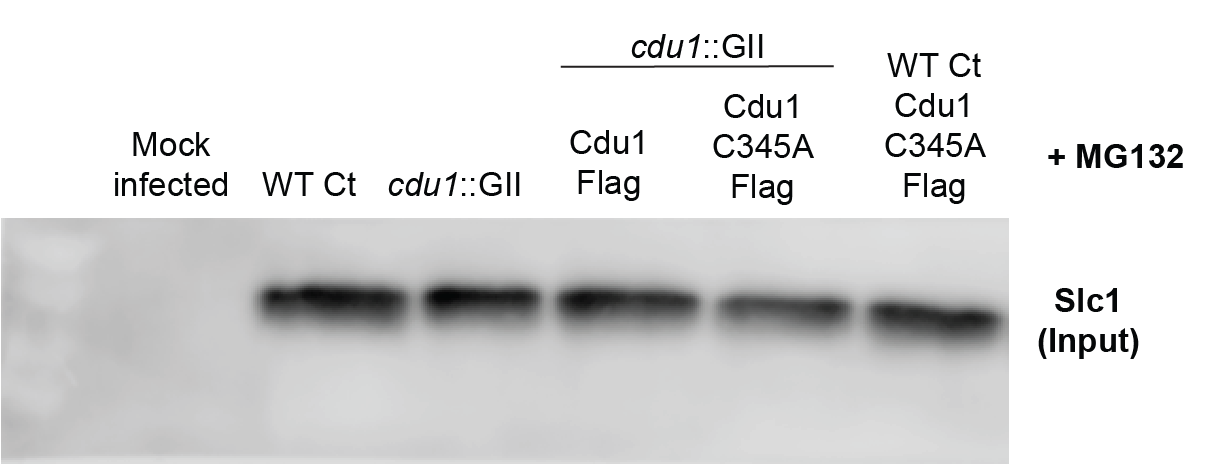

Supplement: Figure 4—source data 8. [file elife-87386-fig4-data8.zip › Figure 4-source data 8/Input_Slc1 WB_Annotated.png]

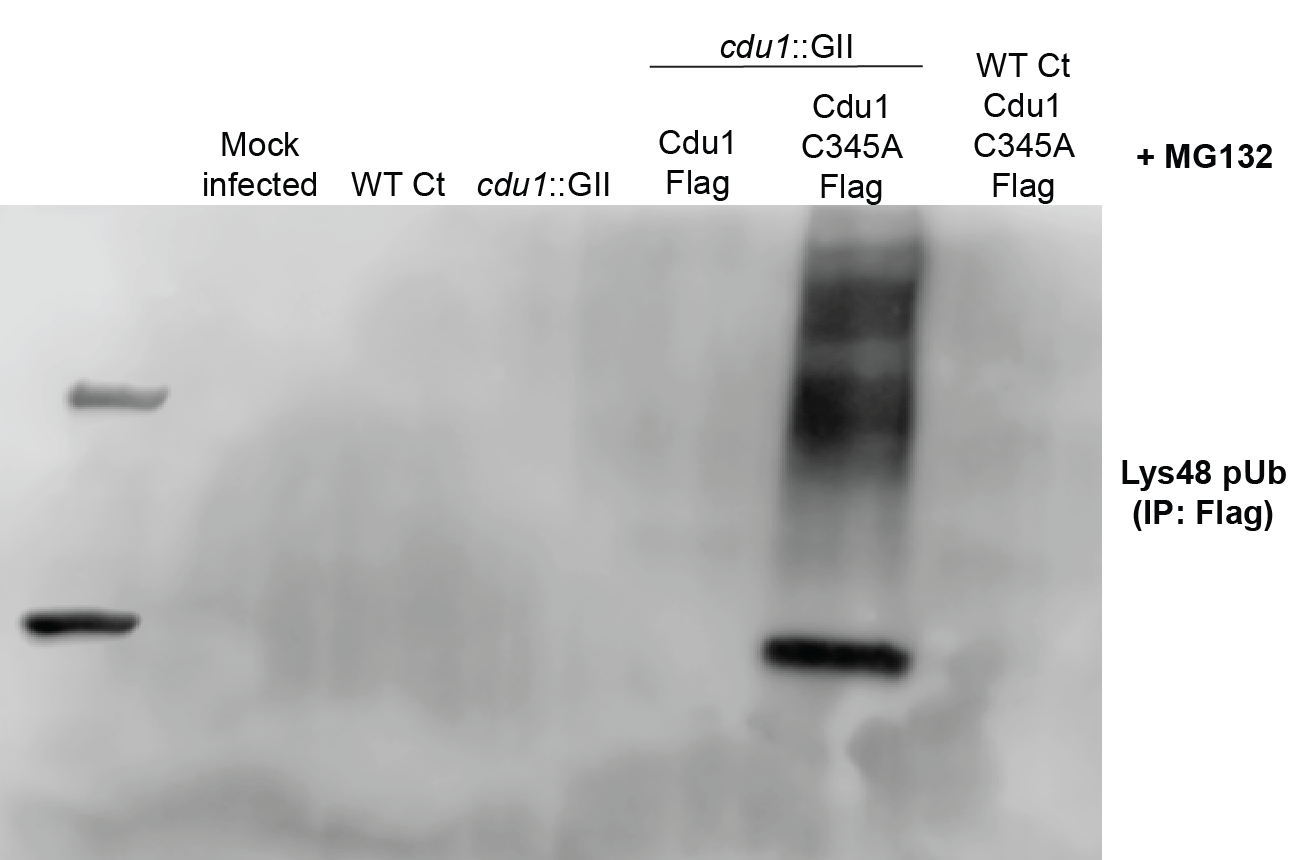

Supplement: Figure 4—source data 8. [file elife-87386-fig4-data8.zip › Figure 4-source data 8/Flag IP_k48 pUb WB_Annotated.png]

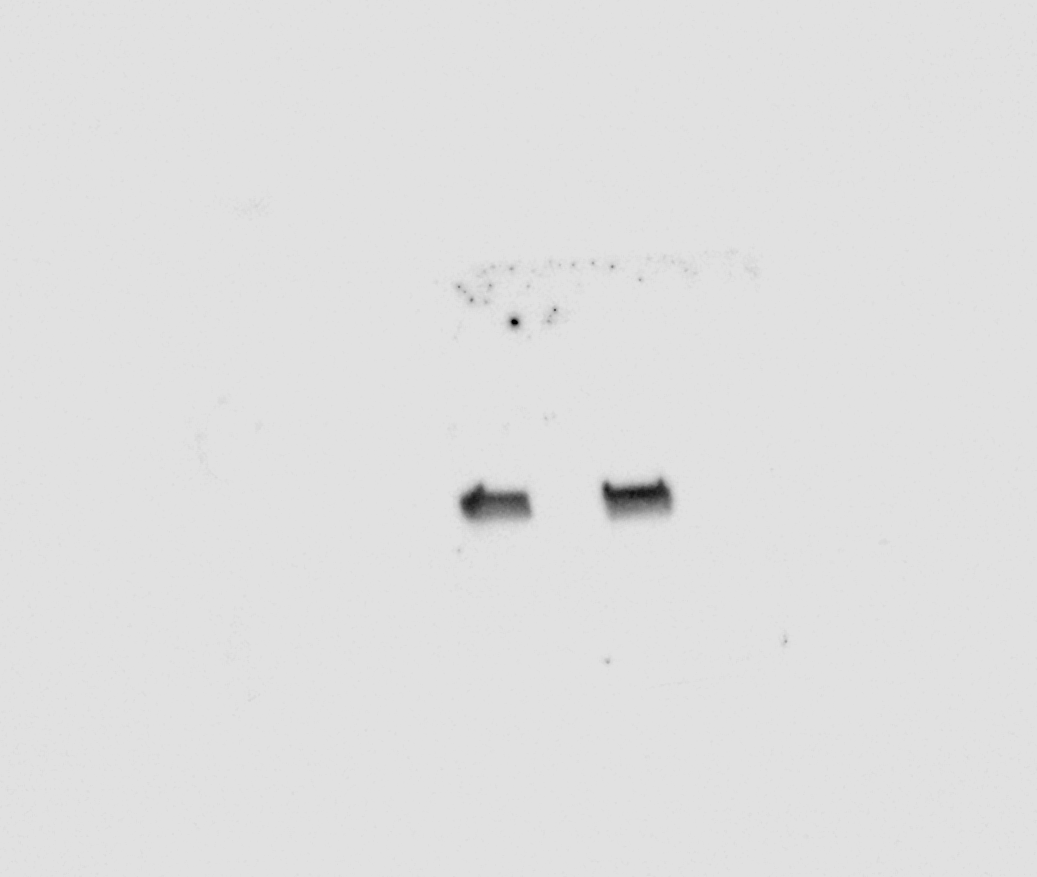

Supplement: Figure 4—source data 9. [file elife-87386-fig4-data9.zip › Figure 4-source data 9/AcK IP_InaC WB.png]

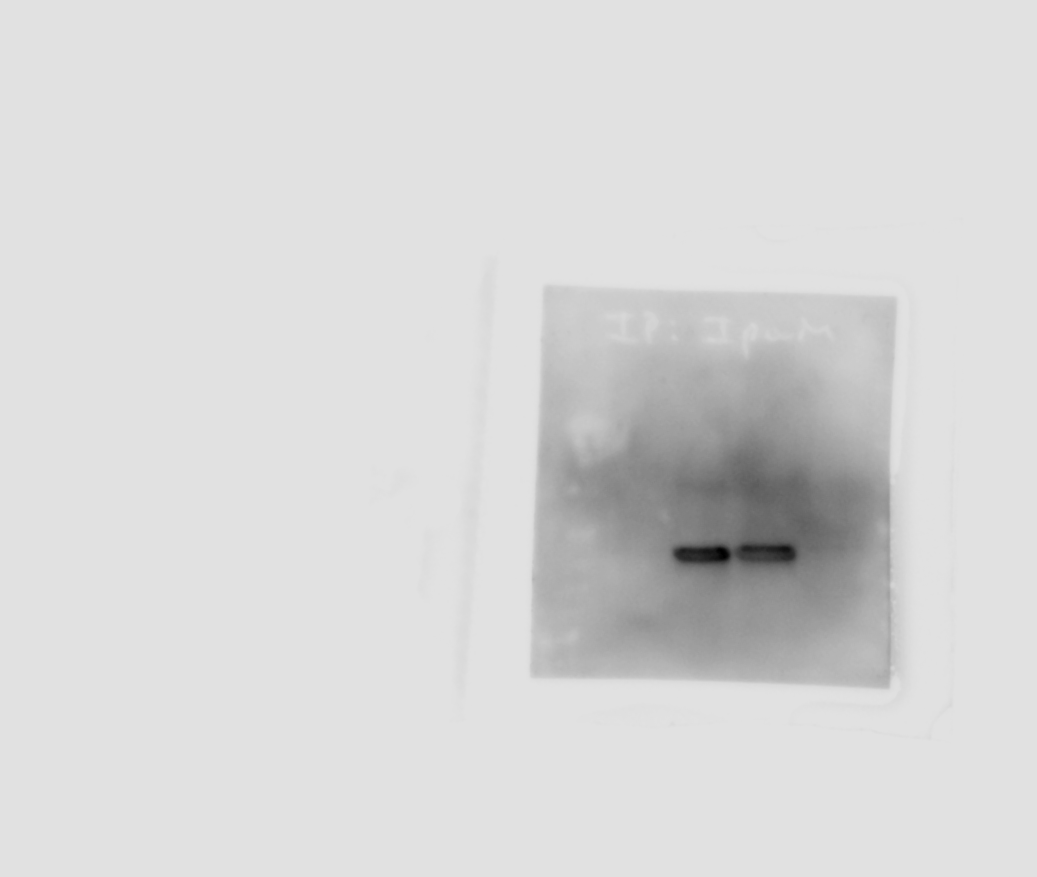

Supplement: Figure 4—source data 9. [file elife-87386-fig4-data9.zip › Figure 4-source data 9/Ac-K IP_IpaM WB.png]

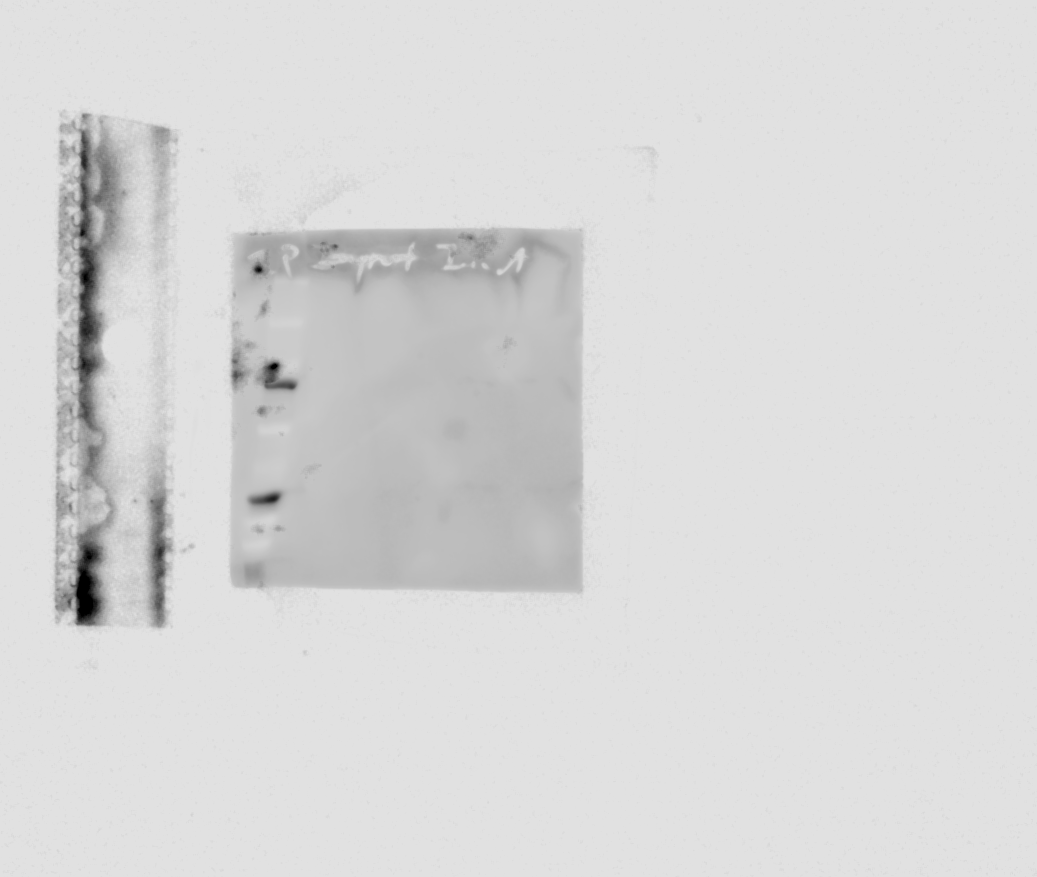

Supplement: Figure 4—source data 9. [file elife-87386-fig4-data9.zip › Figure 4-source data 9/Ac-K IP_IncA WB.png]

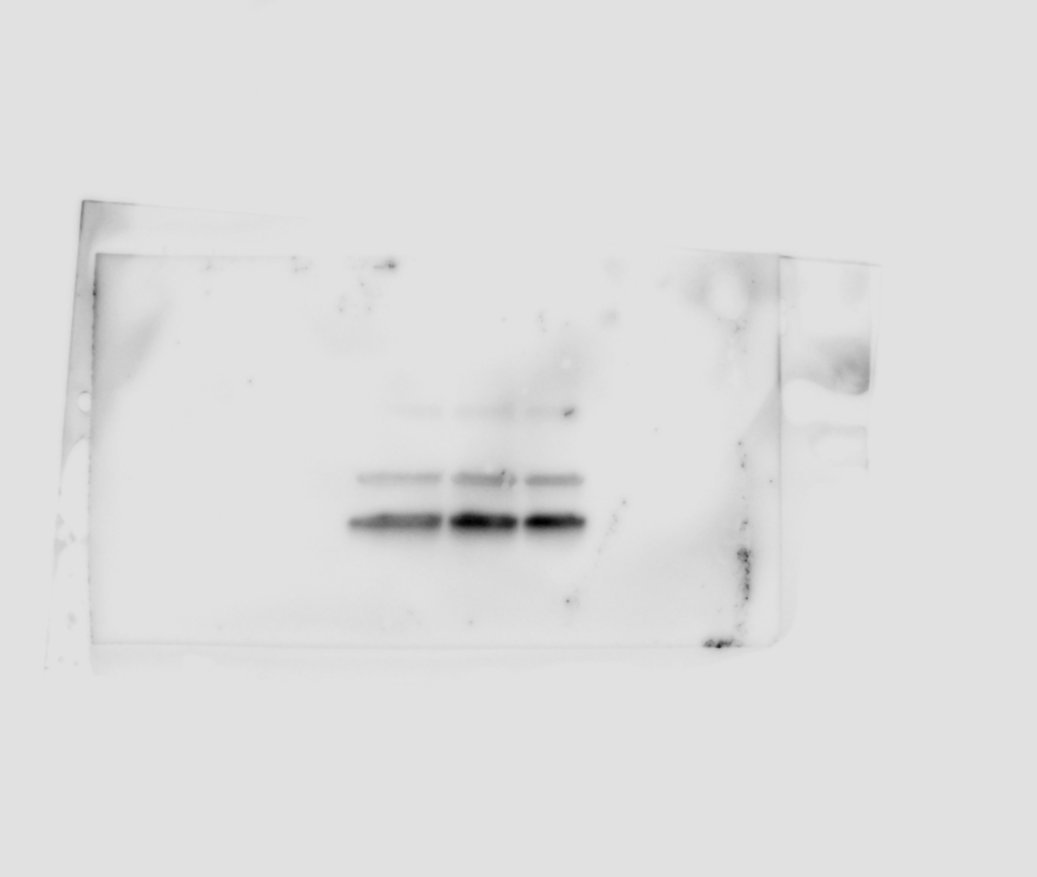

Supplement: Figure 4—source data 9. [file elife-87386-fig4-data9.zip › Figure 4-source data 9/Input_IncA WB.png]

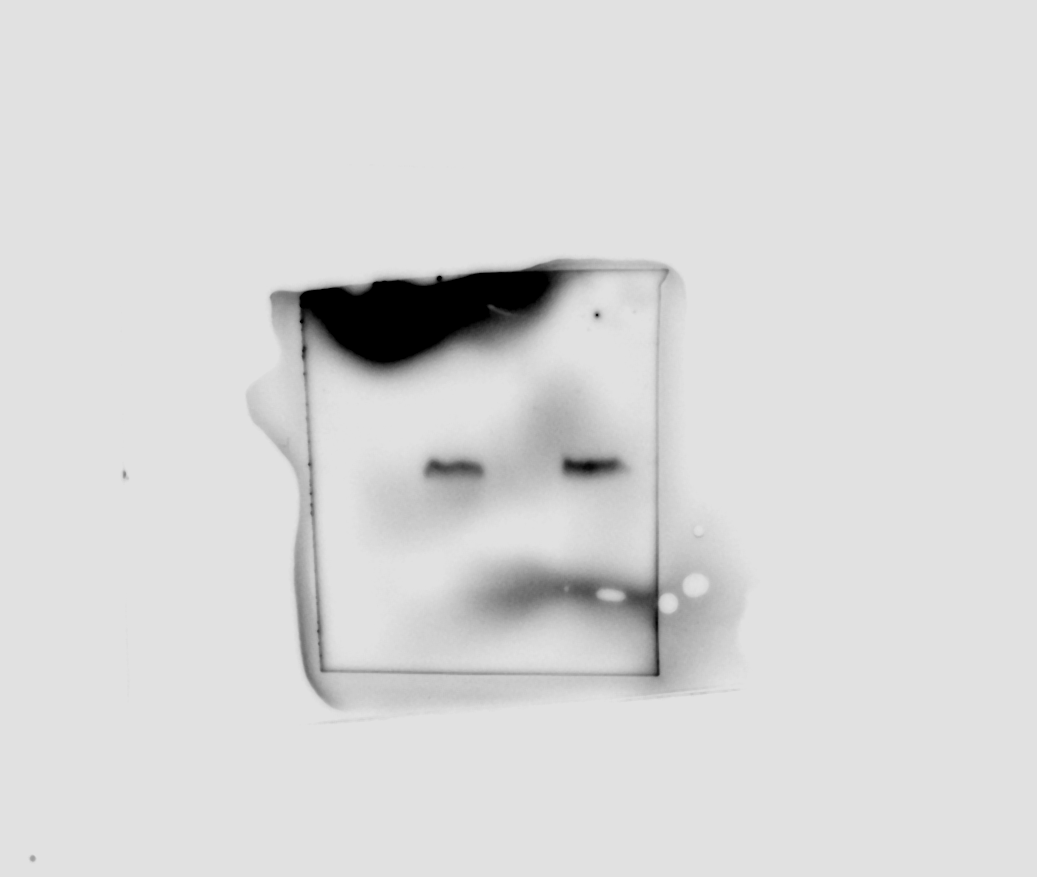

Supplement: Figure 4—source data 9. [file elife-87386-fig4-data9.zip › Figure 4-source data 9/Input_InaC WB.png]

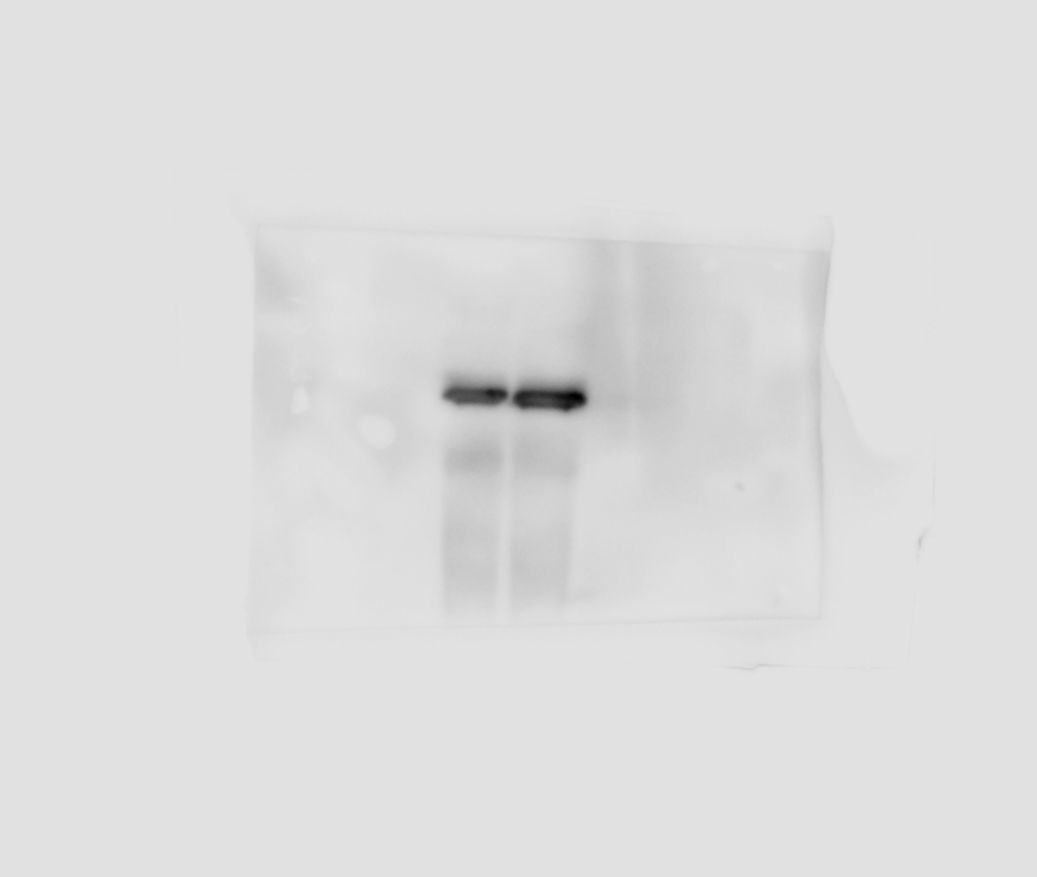

Supplement: Figure 4—source data 9. [file elife-87386-fig4-data9.zip › Figure 4-source data 9/Input_IpaM WB.png]

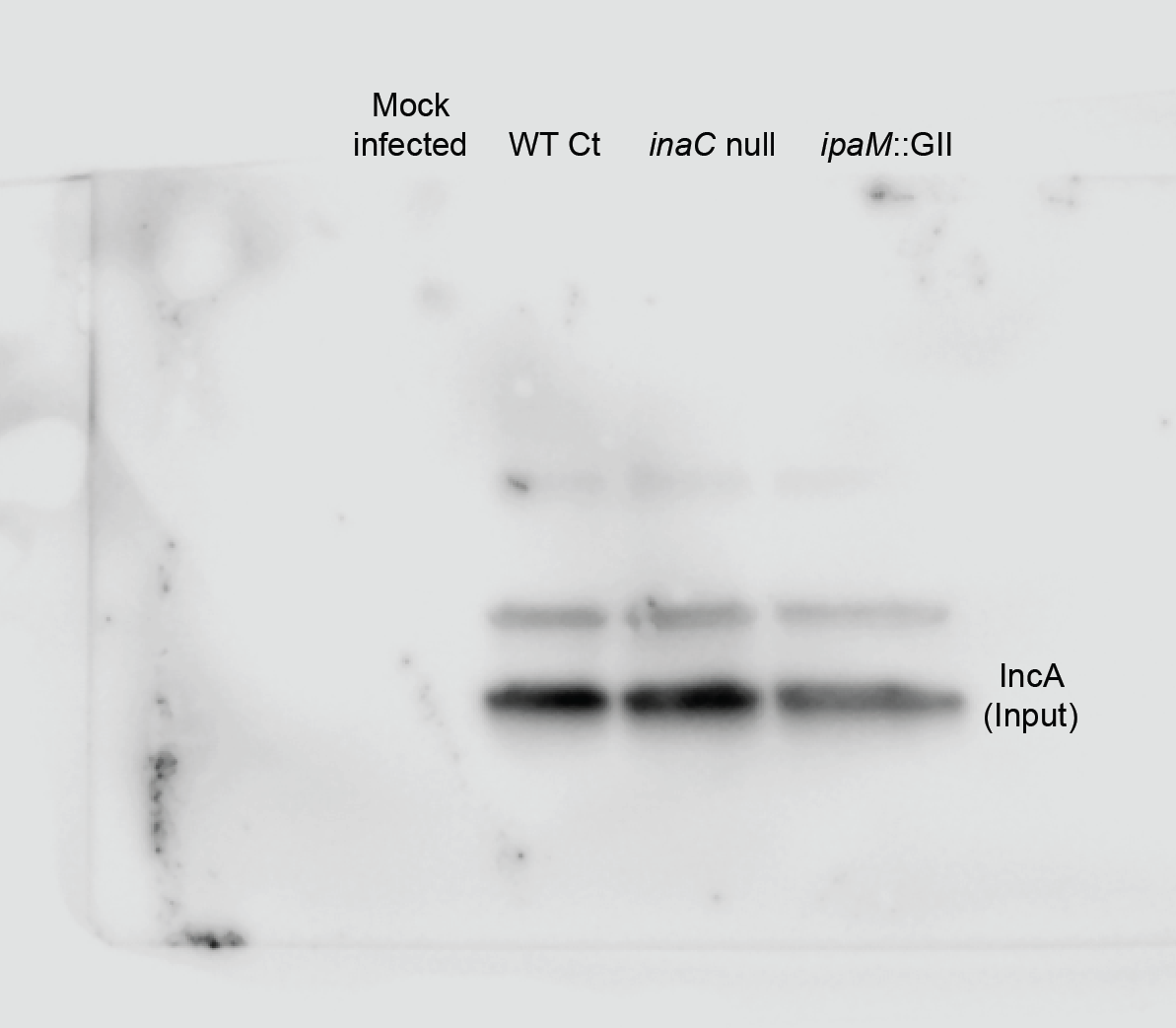

Supplement: Figure 4—source data 10. [file elife-87386-fig4-data10.zip › Figure 4-source data 10/Input_IncA WB_Annotated.png]

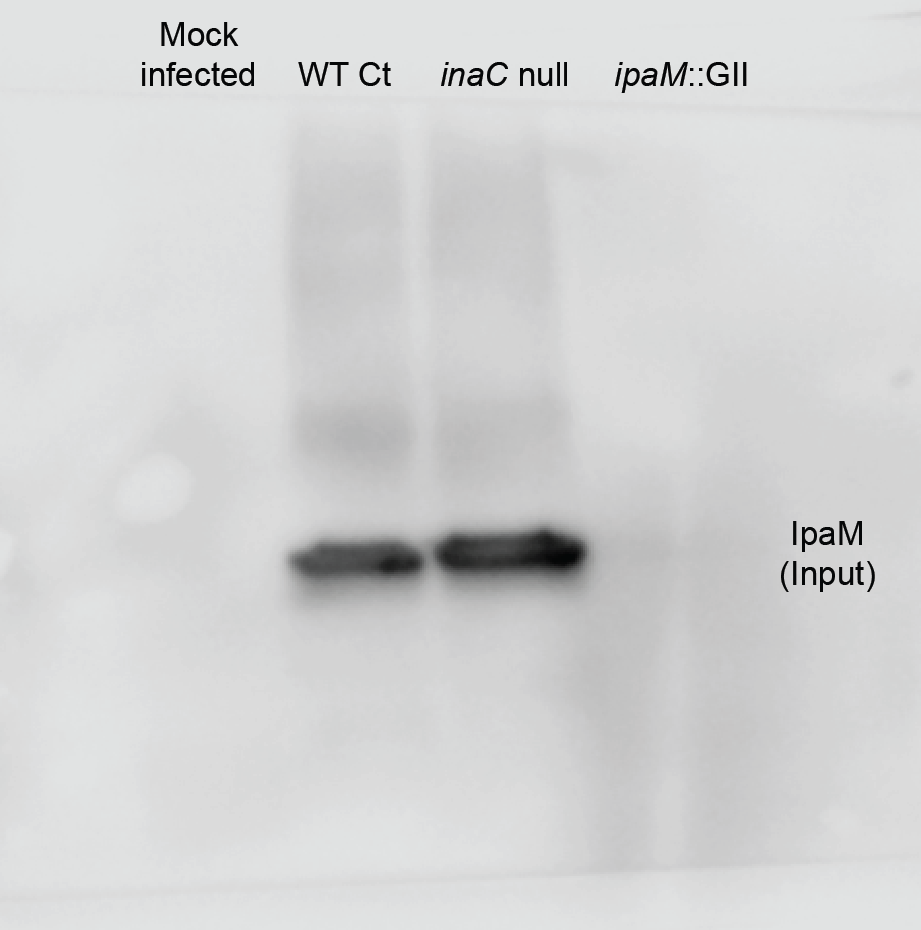

Supplement: Figure 4—source data 10. [file elife-87386-fig4-data10.zip › Figure 4-source data 10/Input_IpaM WB_Annotated.png]

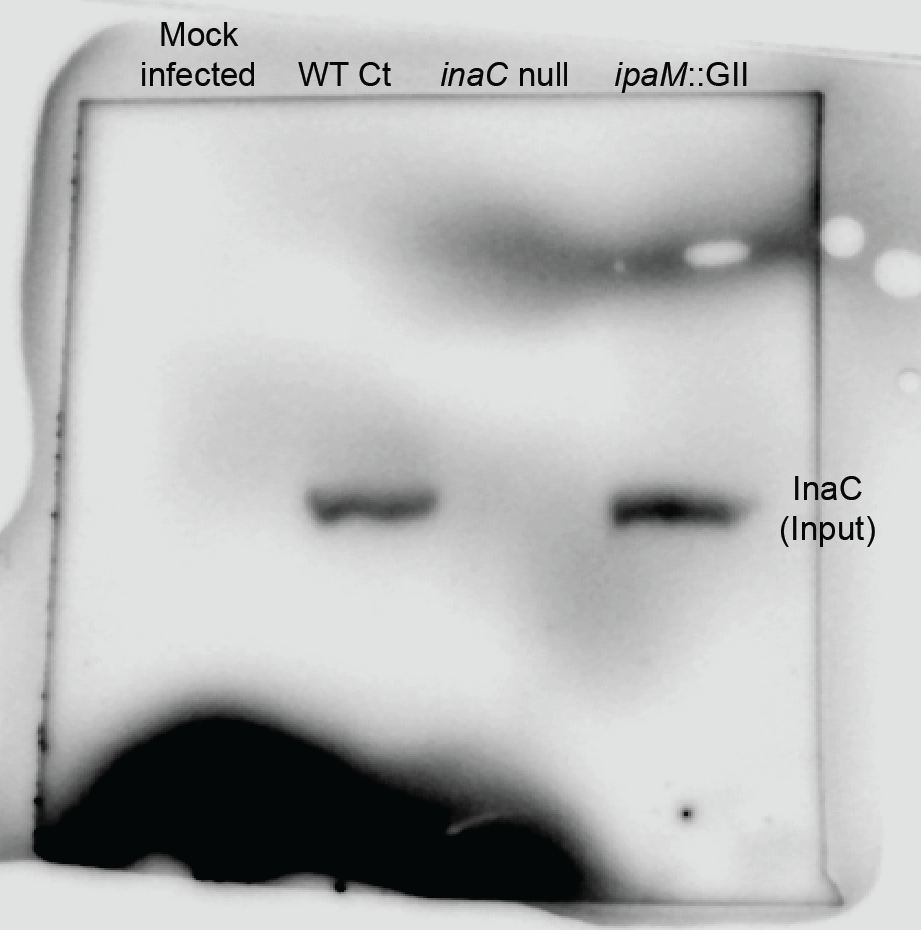

Supplement: Figure 4—source data 10. [file elife-87386-fig4-data10.zip › Figure 4-source data 10/Input_InaC WB_Annotated.png]

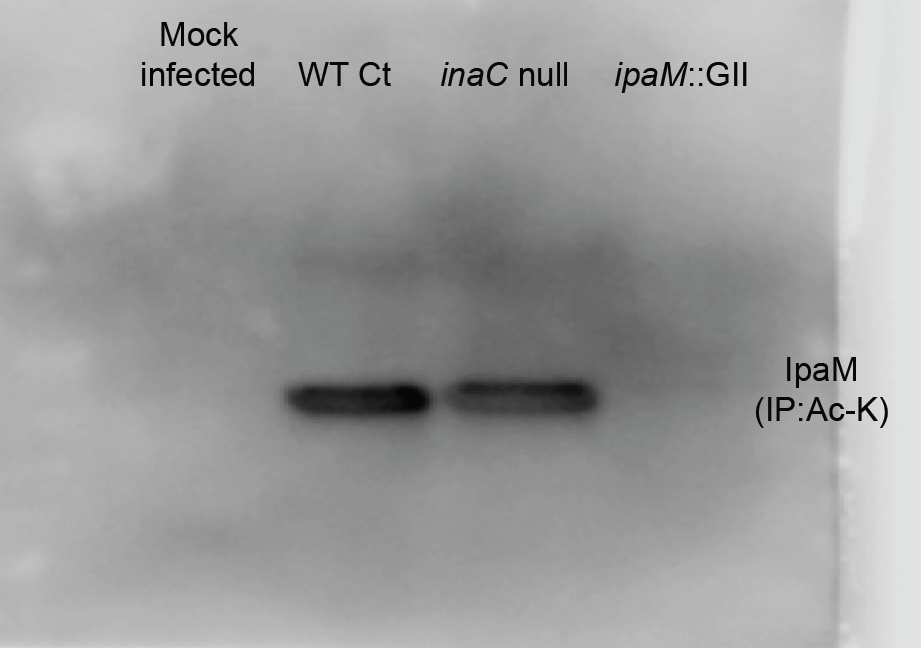

Supplement: Figure 4—source data 10. [file elife-87386-fig4-data10.zip › Figure 4-source data 10/Ac-K IP_IpaM WB_Annotated.png]
