# Supplementary figures and images for: The acetylase activity of Cdu1 regulates bacterial exit from infected cells by protecting Chlamydia effectors from degradation (part 2 of 2)
Source: eLife. 2024 Feb 15;12:RP87386. doi: 10.7554/eLife.87386 (PMC10942603; doi:10.7554/eLife.87386)

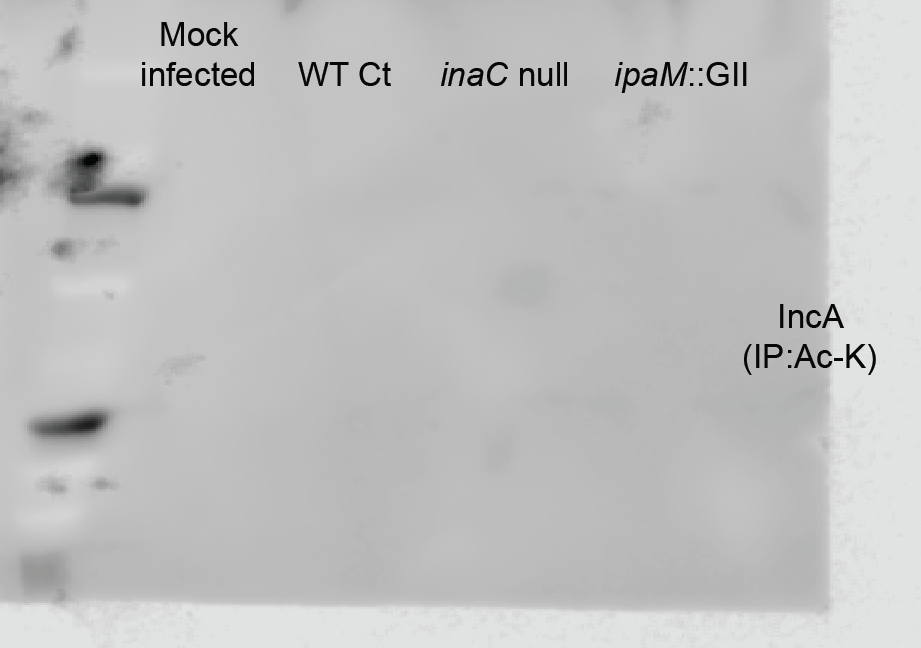

Supplement: Figure 4—source data 10. [file elife-87386-fig4-data10.zip › Figure 4-source data 10/Ac-K IP_IncA WB_Annotated.png]

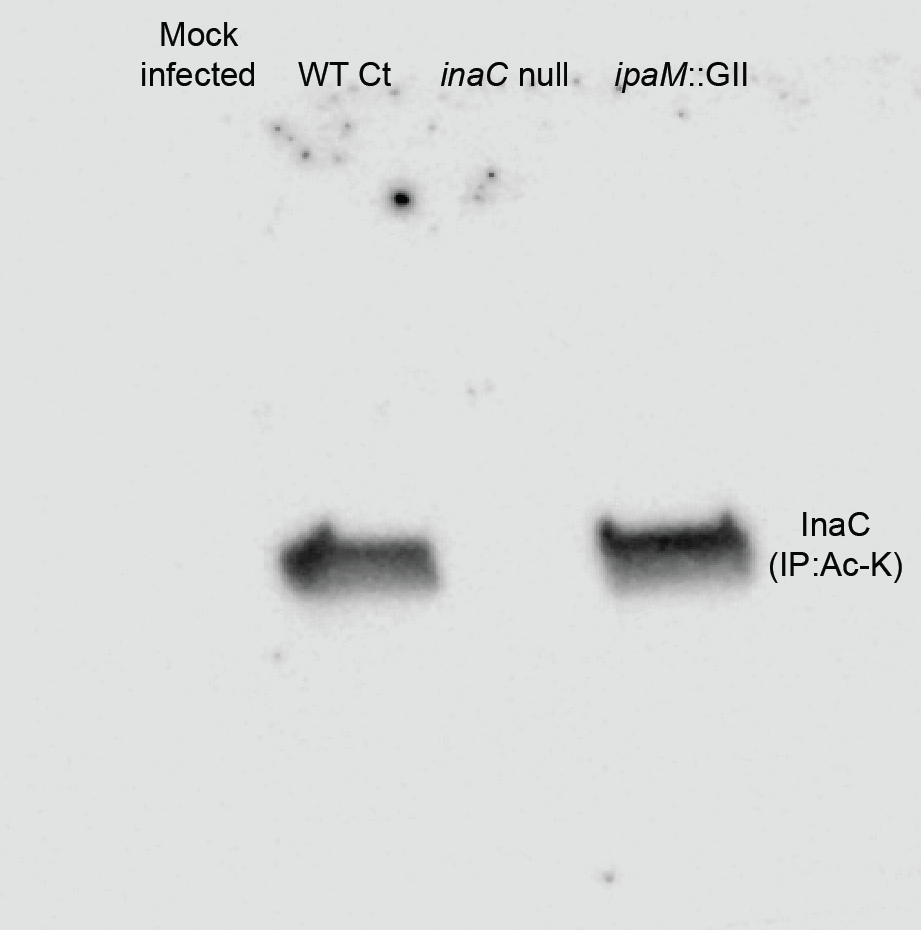

Supplement: Figure 4—source data 10. [file elife-87386-fig4-data10.zip › Figure 4-source data 10/AcK IP_InaC WB_Annotated.png]

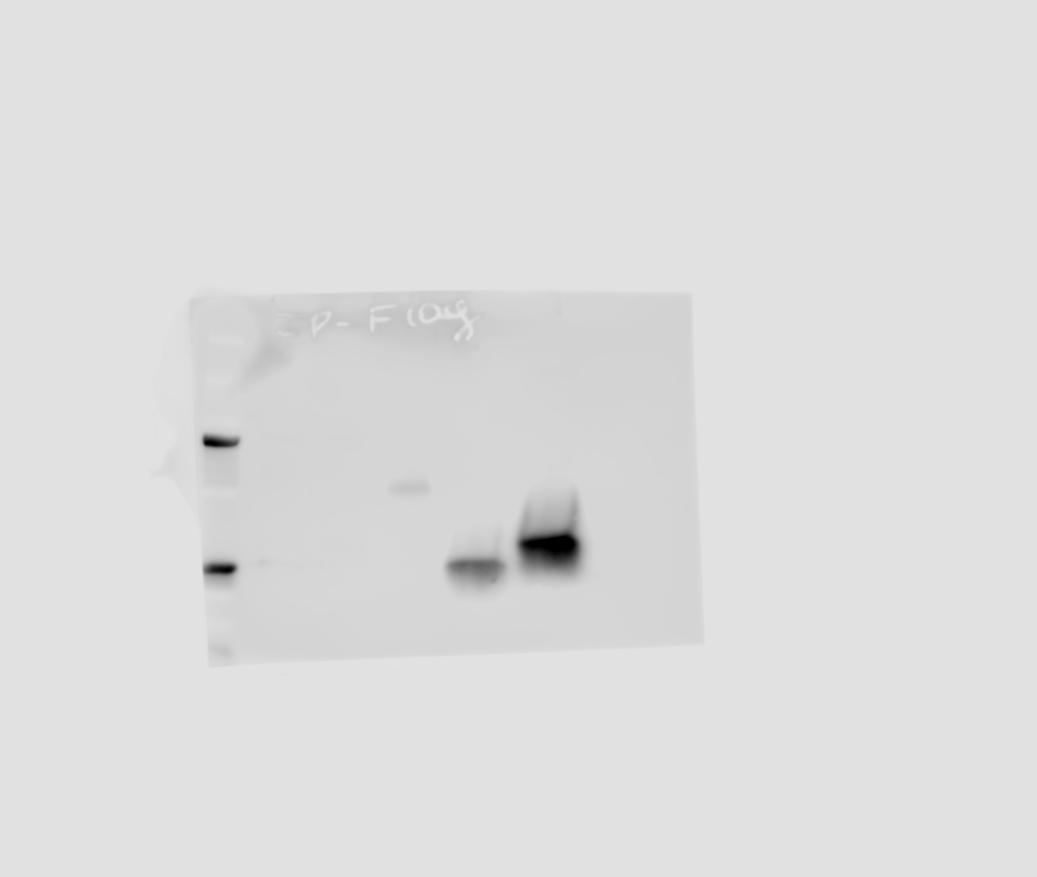

Supplement: Figure 4—source data 11. [file elife-87386-fig4-data11.zip › Figure 4-source data 11/AcK IP_Flag WB.png]

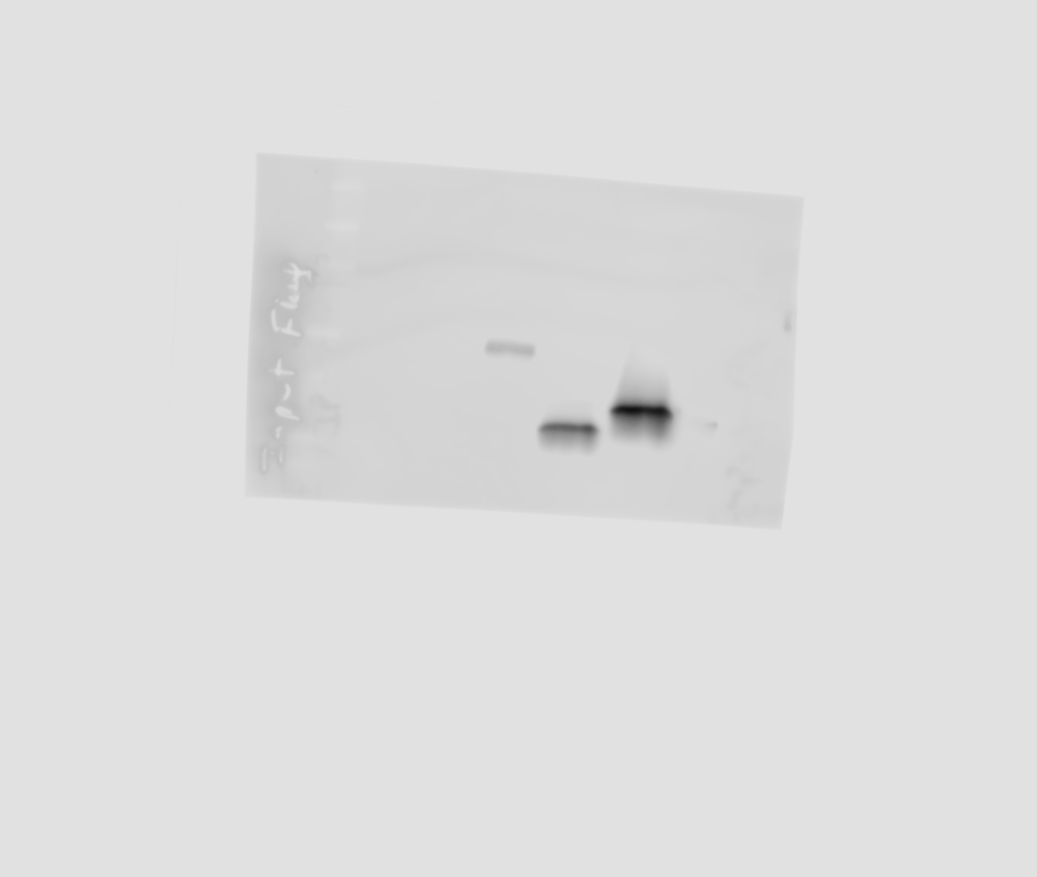

Supplement: Figure 4—source data 11. [file elife-87386-fig4-data11.zip › Figure 4-source data 11/Input_Flag WB.png]

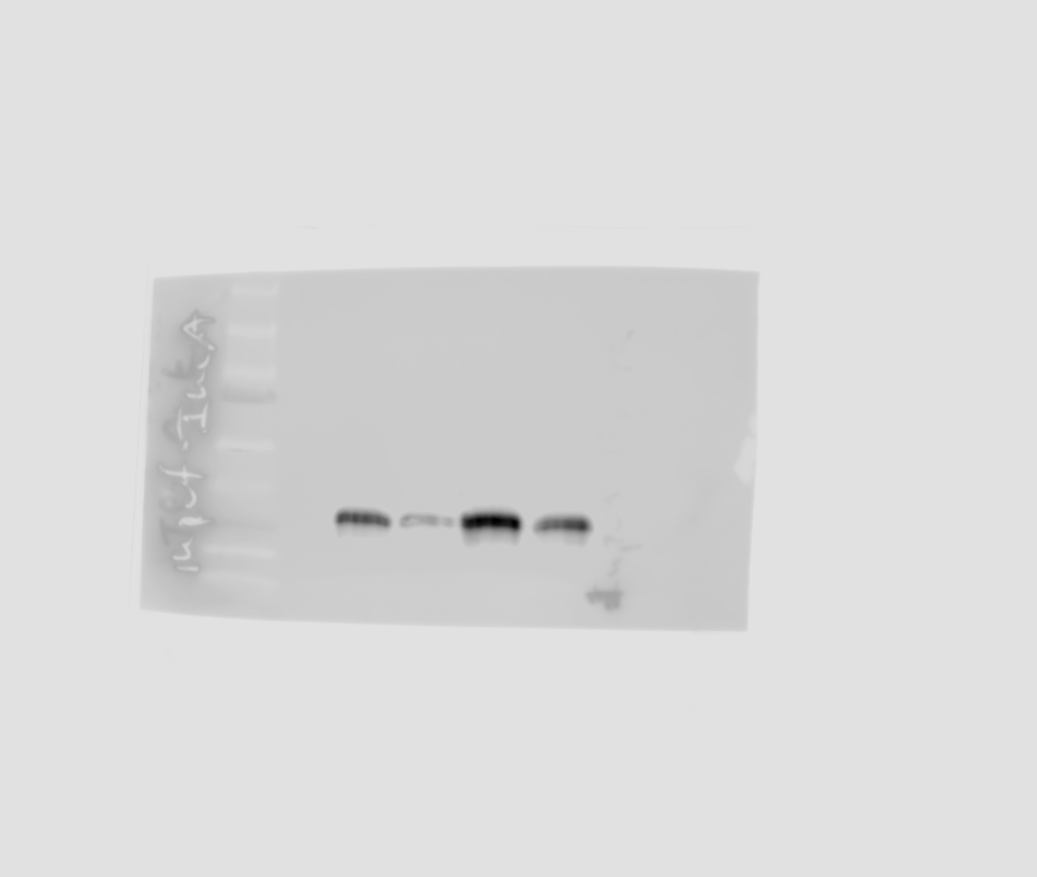

Supplement: Figure 4—source data 11. [file elife-87386-fig4-data11.zip › Figure 4-source data 11/Input_IncA WB.png]

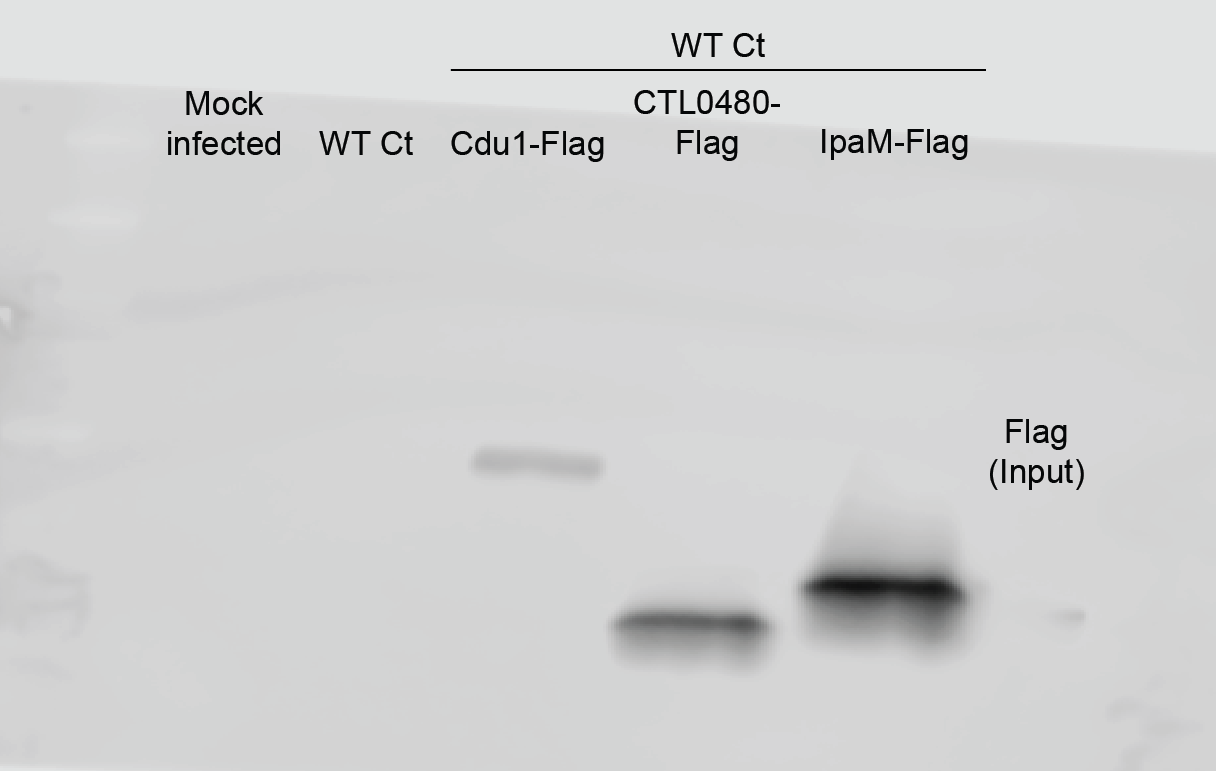

Supplement: Figure 4—source data 12. [file elife-87386-fig4-data12.zip › Figure 4-source data 12/Input_Flag WB_Annotated.png]

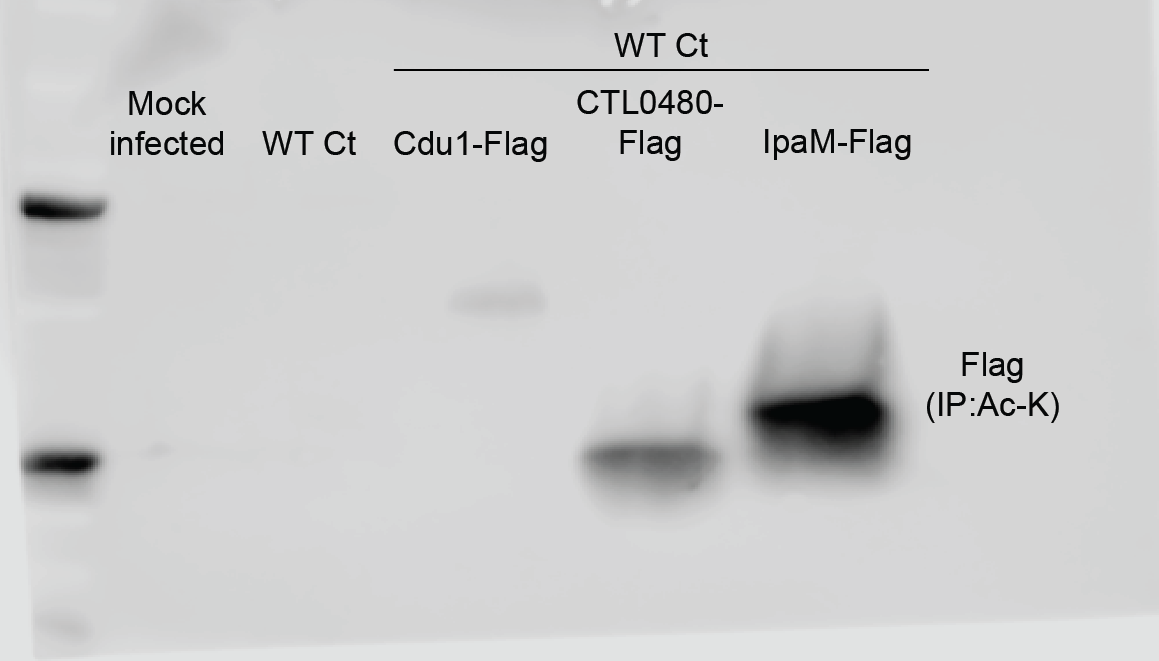

Supplement: Figure 4—source data 12. [file elife-87386-fig4-data12.zip › Figure 4-source data 12/AcK IP_Flag WB_Annotated.png]

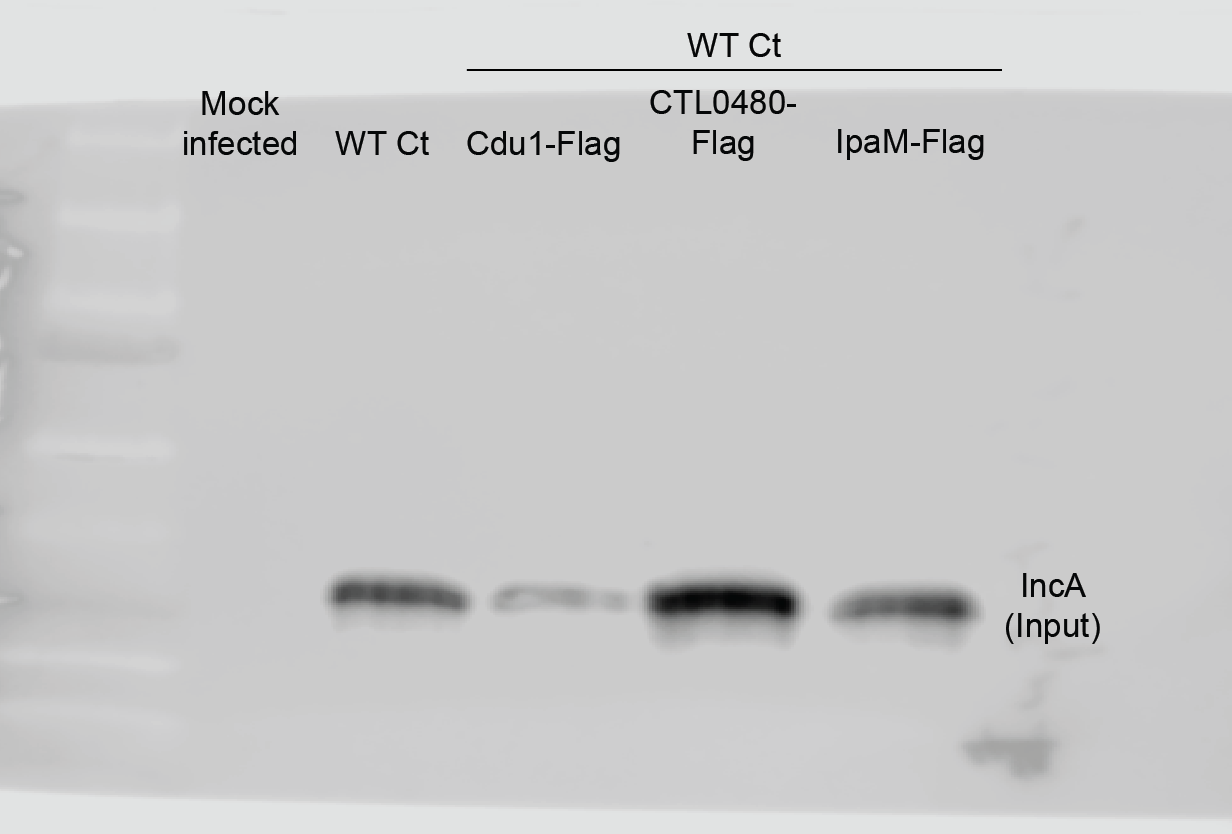

Supplement: Figure 4—source data 12. [file elife-87386-fig4-data12.zip › Figure 4-source data 12/Input_IncA WB_Annotated.png]

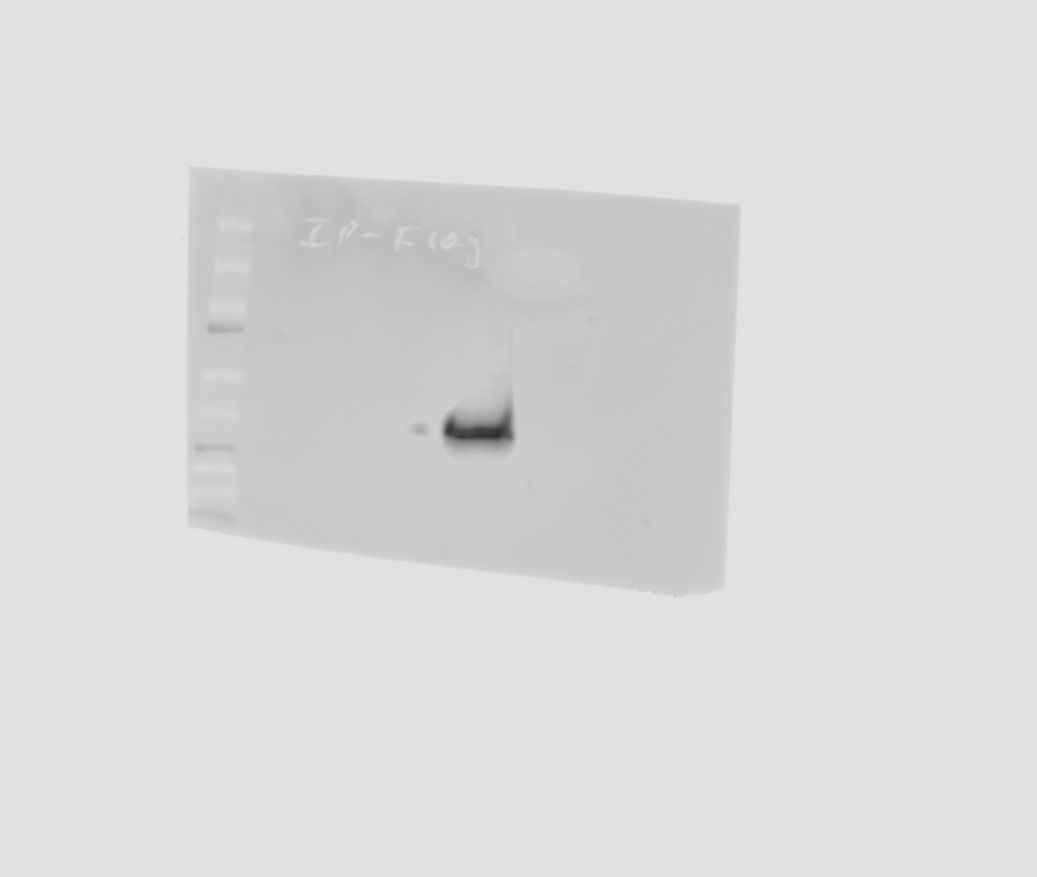

Supplement: Figure 4—source data 13. [file elife-87386-fig4-data13.zip › Figure 4-source data 13/AcK IP_Flag WB.png]

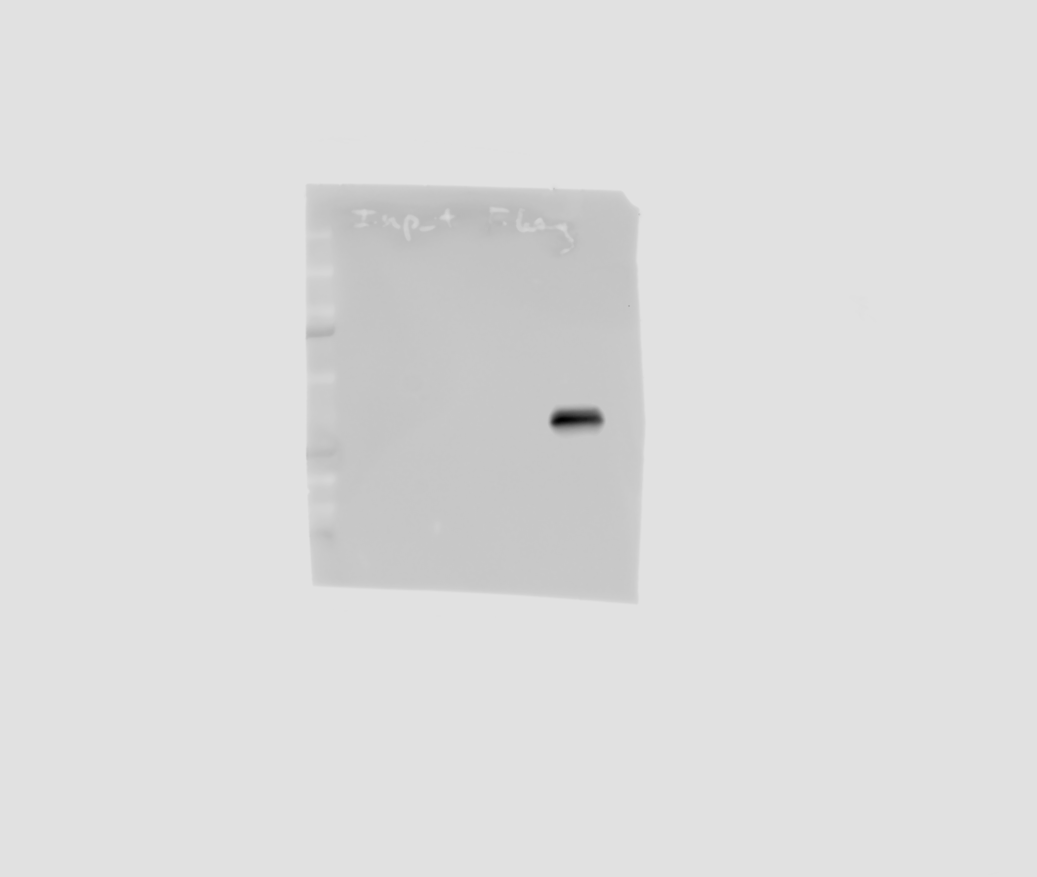

Supplement: Figure 4—source data 13. [file elife-87386-fig4-data13.zip › Figure 4-source data 13/Input_Flag WB.png]

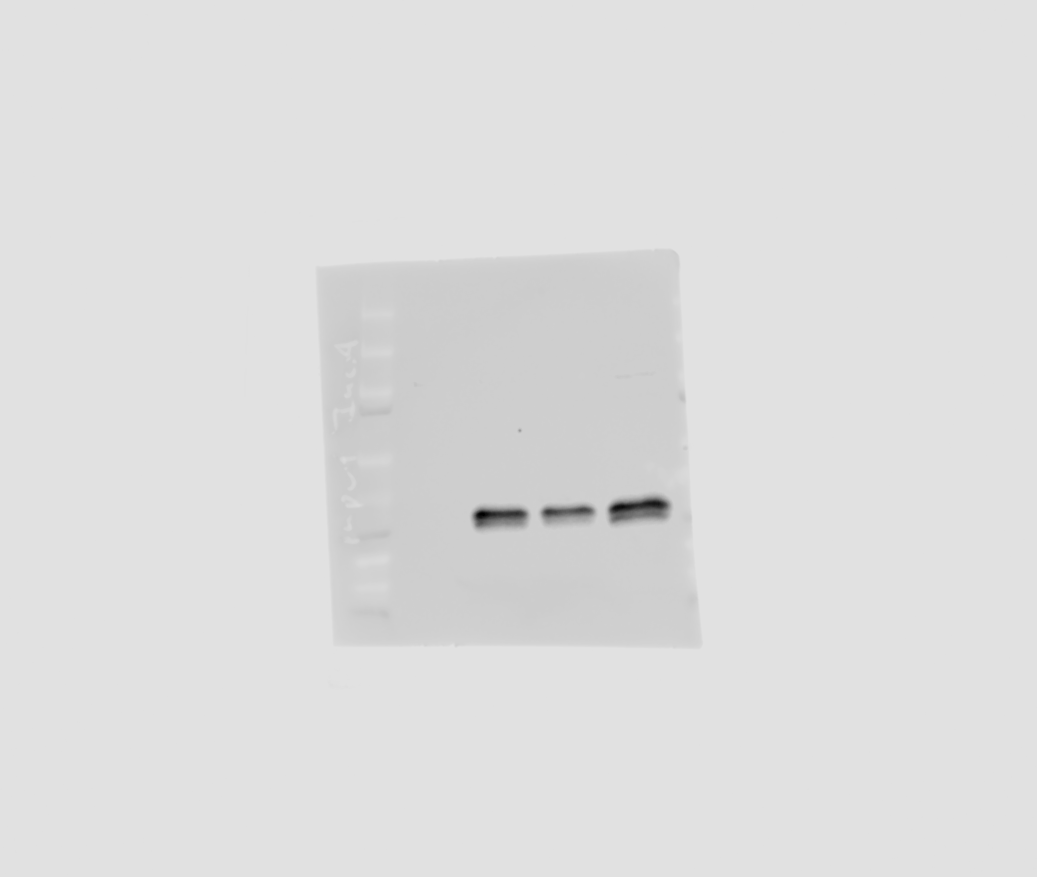

Supplement: Figure 4—source data 13. [file elife-87386-fig4-data13.zip › Figure 4-source data 13/Input_IncA WB.png]

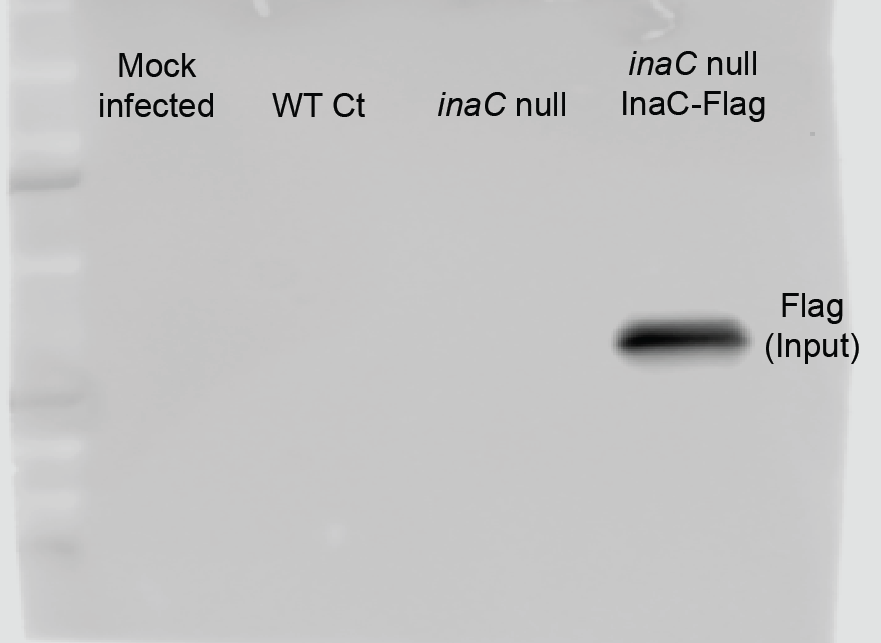

Supplement: Figure 4—source data 14. [file elife-87386-fig4-data14.zip › Figure 4-source data 14/Input_Flag WB_Annotated.png]

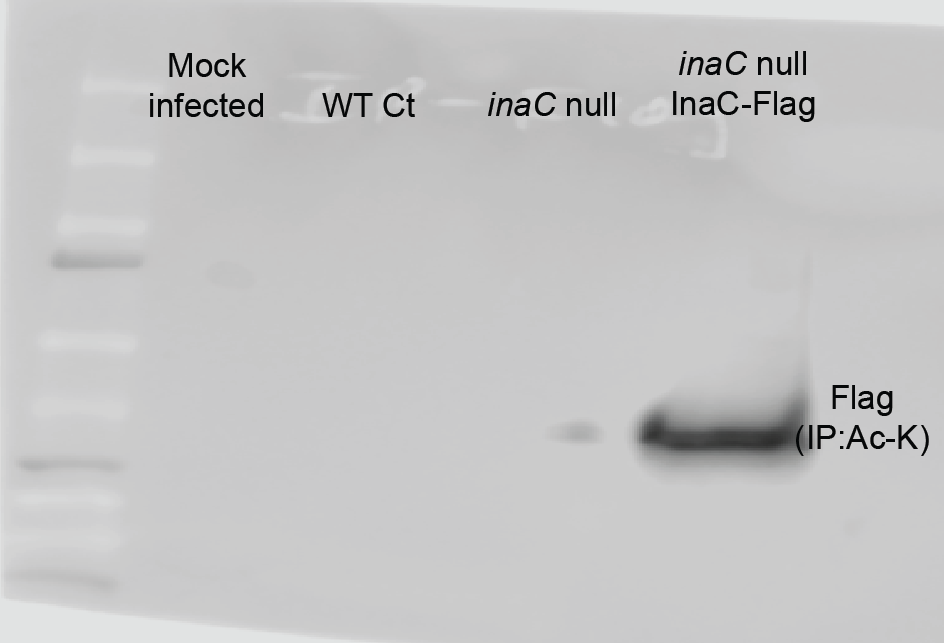

Supplement: Figure 4—source data 14. [file elife-87386-fig4-data14.zip › Figure 4-source data 14/AcK IP_Flag WB_Annotated.png]

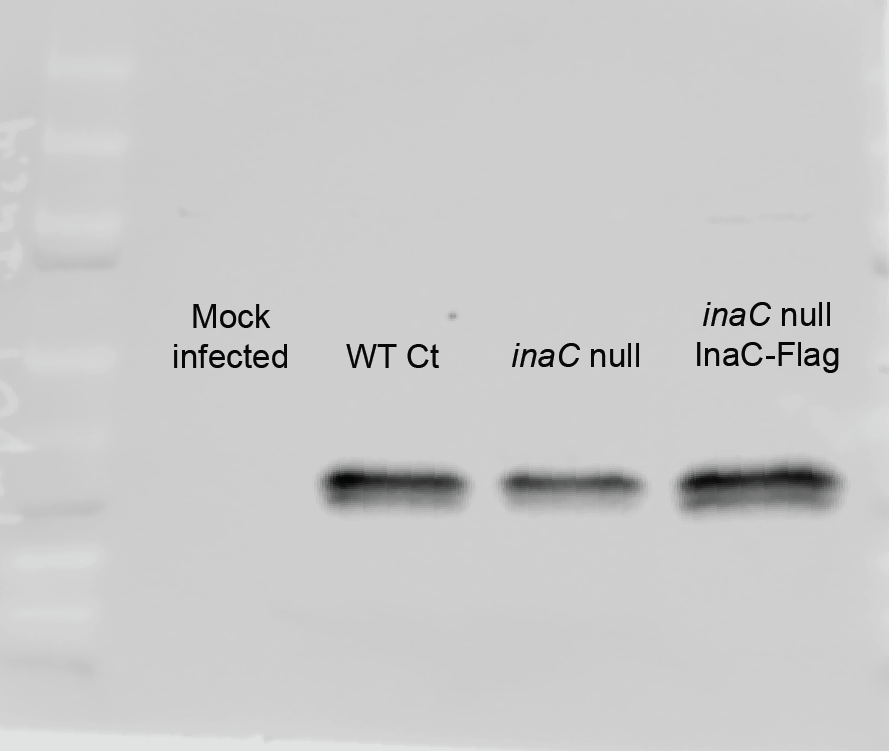

Supplement: Figure 4—source data 14. [file elife-87386-fig4-data14.zip › Figure 4-source data 14/Input_IncA WB_Annotated.png]
